# Rapid and repeated evolution of increased competitive ability in a global invader

**DOI:** 10.1038/s41467-026-77819-z

**Published:** 2026-09-17

**Authors:** Dávid U. Nagy, Ragan M. Callaway, S. Luke Flory, Christopher D. Barratt, Walter Durka, Ylva Lekberg, Marilia S. Lucas, Stefan G. Michalski, Renske E. Onstein, Robert W. Pal, Julian Selke, Arpad E. Thoma, Mohammad M. Al-Gharaibeh, Ali A. Al-Namazi, Guadalupe Andraca-Gómez, Pål Axel Olsson, Shukherdorj Baasanmunkh, Sirwan Babaei, Fernando Bastida, Jonathan A. Bennett, Caio Brunharo, Darron A. Cullen, Ryan C. Donnelly, Alyson Ennis, David J. Ensing, Özkan Eren, Fatih Fazlioglu, Rita Filep, Michael Flessner, Lauren J. Frazee, Luís González, Zigmantas Gudžinskas, C. Matt Guilliams, Avni Hajdari, Erin R. Haramoto, Sean R. Haughian, J. Mason Heberling, Isabell Hensen, Emily M. Holden, Kateřina Jandová, Amit Jhala, Xin Jia, Li Jun, Matthew A. Kaproth, Hesham Kassem, Abdelmajid Khabbach, Damase P. Khasa, Matthew H. Koski, Kevin Kožić, Nikos Krigas, Greta La Bella, Jesse R. Lasky, Benjamin Lee, Elizabeth Leger, Joanna M. Lemly, Mohamed Libiad, Paula Lorenzo, Vanessa Lozano, Jacob E. Lucero, Trobjon Makhkamov, Elizabete Marchante, Helia Marchante, Valerie Marshall, Chandra E. Moffat, Abigail J. Moore, Sándor Nagy, Anush Nersesyan, Andriy Novikov, Miki Okada, Adrian Oprea, Rafael Otfinowski, Astghik Papikyan, Christopher A. Proctor, Satu Ramula, Emily S. J. Rauschert, Bryan Reatini, Christian Rixen, Scott Rush, Gemma Rutten, Amir Sadeghpour, Ioulietta Samartza, Simona Sarmati, J. F. Scheepens, Manzoor A. Shah, Min Sheng, Peter Sikkema, Cathy Silliman, Mandy L. Slate, Samuel B. St. Clair, Claudia Stein, Klara Stevermer, Satoshi N. Suzuki, Yigitali Tashpulatov, Tomonori Tsunoda, Orzimat Turginov, Tatyana Vakhlamova, Nicole M. van Dam, Viktoria Wagner, Lydia Westberg, Sa Xiao, Zhijie Zhang, Christoph Rosche

**Affiliations:** 1 Plant Evolutionary Ecology, Faculty of Biological Sciences, Goethe University Frankfurt, Frankfurt am Main, Germany; 2 Institute of Biology/Geobotany and Botanical Garden, Martin Luther University Halle-Wittenberg, Halle, Germany; 3 Division of Biological Sciences, University of Montana, Missoula, MT USA; 4 Agronomy Department, University of Florida, Gainesville, FL USA; 5 Invasion Science Institute, University of Florida, Gainesville, FL USA; 6 German Centre for Integrative Biodiversity Research (iDiv) Halle-Jena-Leipzig, Leipzig, Germany; 7 University of Leipzig, Ritterstrasse 26, Leipzig, Germany; 8 Animal Breeding and Genomics, Wageningen University & Research, Droevendaalsesteeg 1, Wageningen, The Netherlands; 9 Department of Community Ecology, Helmholtz Centre for Environmental Research–UFZ, Halle (Saale), Germany; 10 MPG Ranch Missoula, Florence, MT USA; 11 Department of Ecosystem and Conservation Sciences, W.A. Franke College of Forestry and Conservation, University of Montana, Missoula, MT USA; 12 Naturalis Biodiversity Center, Darwinweg 2, Leiden, The Netherlands; 13 Institute of Biology Leiden (IBL), Leiden University, Sylviusweg 72, Leiden, The Netherlands; 14 Department of Biological Sciences, Montana Technological University, Butte, MT USA; 15 Faculty of Informatics and Data Science, University of Regensburg, Regensburg, Germany; 16 Department of Plant Production, Jordan University of Science and Technology, Irbid, Jordan; 17 Department of Integrative Agriculture, United Arab Emirates University, Al Ain, United Arab Emirates; 18 National Center for Vegetation Cover Development and Combating Desertification (NCVC), 6336 Northern Ring Branch Road, Al Nafal, 3372, Riyadh, Saudi Arabia; 19 Morooj National Foundation, Riyadh, Saudi Arabia; 20 Instituto de Biología, Universidad Nacional Autónoma de México, Ciudad Universitaria, Ciudad de México, México; 21 Biodiversity, Department of Biology, Lund University, Lund, Sweden; 22 Department of Biology and Chemistry, Changwon National University, Changwon, South Korea; 23 Department of Plant Production and Genetics, University of Kurdistan, Sanandaj, Iran; 24 School of Agricultural Sciences, College of Agriculture, Life, and Physical Sciences, Southern Illinois University, Carbondale, IL USA; 25 Departamento CIencias Agroforestales, Universidad de Huelva, Huelva, Spain; 26 Department of Plant Sciences, University of Saskatchewan, Saskatoon, SK Canada; 27 Department of Plant Science, Pennsylvania State University, University Park, PA USA; 28 School of Environmental and Life Sciences, University of Hull, Hull, UK; 29 Division of Biology, Kansas State University, Manhattan, KS USA; 30 Department of Ecology and Evolutionary Biology, University of Colorado Boulder, Boulder, CO USA; 31 Summerland Research and Development Centre, Agriculture and Agri-Food Canada, Summerland, BC Canada; 32 Department of Biology, Faculty of Science, Aydın Adnan Menderes University, Aydın, Türkiye; 33 Plant Ecology, University of Bayreuth, Universitätsstraße 30, Bayreuth, Germany; 34 Department of Pharmacognosy, Faculty of Pharmacy, University of Pécs, Pécs, Hungary; 35 School of Plant and Environmental Sciences, Virginia Tech, Blacksburg, VA USA; 36 Cencora / PharmaLex, Kirschberg, NJ USA; 37 CISPAC. Universidade de Vigo. Department of Plant Biology and Soil Sciences, Faculty of Biology, Vigo, Spain; 38 State Scientific Research Institute Nature Research Centre, Laboratory of Flora and Geobotany, Vilnius, Lithuania; 39 Department of Conservation and Research, Santa Barbara Botanic Garden, Santa Barbara, California, US; 40 Department of Biology, Faculty of Mathematical and Natural Sciences, University of Prishtina, Eqrem Çabej St., 51, Prishtina, Republic of Kosovo; 41 Plant and Soil Sciences Department, University of Kentucky, Lexington, KY USA; 42 Herbarium of the Nova Scotia Museum, 1747 Summer Street, Halifax, NS Canada; 43 Section of Botany, Carnegie Museum of Natural History, Pittsburgh, PA USA; 44 Alberta Biodiversity Monitoring Institute, University of Alberta, Edmonton, Alberta Canada; 45 Institute for Environmental Studies, Faculty of Science, Charles University, Prague, Czech Republic; 46 Department of Agronomy and Horticulture, University of Nebraska, Lincoln, USA; 47 Yanchi Research Station, School of Soil and Water Conservation, Beijing Forestry University, Beijing, China; 48 State Key Laboratory of Desert and Oasis Ecology, Xinjiang Institute of Ecology and Geography, Chinese Academy of Sciences, Urumqi, China; 49 Akesu National Station of Observation and Research for Oasis Agro-ecosystem, Akesu, Xinjiang China; 50 University of Chinese Academy of Sciences, Beijing, China; 51 Department of Biological Sciences, Minnesota State University Mankato, Mankato, MN USA; 52 Alexander von Humboldt (AvH) Research Fellow, Cairo, Egypt; 53 Biotechnology, Environment, Agri-Food and Health Laboratory, Faculty of Sciences, Sidi Mohamed Ben Abdellah University, Dhar El Mahraz, Fez Morocco; 54 Institute of Integrative and Systems Biology and Centre for Forest Research, Université Laval, Québec, QC Canada; 55 Department of Biological Sciences, Clemson University, Clemson, SC USA; 56 Institute of Plant Breeding and Genetic Resources, Hellenic Agricultural Organization Demeter (ELGO-Dimitra), Thessaloniki, Thermi Greece; 57 Department of Science, University of Roma Tre, Viale Guglielmo Marconi 446, Roma, Italy; 58 Department of Biology, Pennsylvania State University, State College, PA, USA; 59 Department of Biological Sciences, East Tennessee State University, 1276 Gilbreath Dr., Johnson City, TN USA; 60 University of Michigan Institute for Global Change Biology, 440 Church Street, Ann Arbor, MI USA; 61 University of Nevada, Reno, 1664 N. Virginia St., Reno, NV USA; 62 Colorado Natural Heritage Program, Colorado State University, Fort Collins, CO USA; 63 Ecology, Systematics, and Biodiversity Conservation Team (ESCB) URL-CNRST N_18, FS, Abdelmalek Essaadi University, M’Hannech II, Tetouan, Morocco; 64 Centre for Functional Ecology, Associate Laboratory TERRA, Department of Life Sciences, University of Coimbra, Coimbra, Portugal; 65 Department of Agricultural Sciences, University of Sassari, Viale Italia 39/A, Sassari, Italy; 66 Department of Rangeland, Wildlife and Fisheries Management, Texas A&M University, College Station, TX USA; 67 Department of Forestry and Landscape Design, Tashkent State Agrarian University, 2 A., Universitet Str., Kibray District, Tashkent, Uzbekistan; 68 Department of Botany and Genetics, National University of Uzbekistan, 4 University Str., Tashkent, Uzbekistan; 69 Research Centre for Natural Resources, Environment and Society (CERNAS), Polytechnic Institute of Coimbra, Coimbra Agriculture School, Bencanta, Coimbra, Portugal; 70 University of Oklahoma. Oklahoma Biological Survey and School of Biological Sciences, 770 Van Vleet Oval, Norman, OK USA; 71 József A. utca 47, Rácalmás, Hungary; 72 A. Takhtajan Institute of Botany, Acharyan 1, Yerevan, Armenia; 73 Nature Heritage NGO, Luxemburg 9-38, Yerevan, Armenia; 74 State Museum of Natural History of the NAS of Ukraine, Teatralna str. 18, Lviv, Ukraine; 75 Department of Molecular and Cell Biology, University of California, Berkeley, Berkeley, CA USA; 76 University “Alexandru Ioan Cuza”, Botanical Garden, 7-9, Dumbrava Rosie Street, Iasi, Romania; 77 Department of Biology, University of Winnipeg, 515 Portage Avenue, Winnipeg, MB Canada; 78 Department of Biology, University of Turku, Turku, Finland; 79 Cleveland State University, Cleveland, OH USA; 80 United States Forest Service, Roseburg, OR USA; 81 WSL-Institute for Snow and Avalanche Research SLF, Davos, Switzerland; 82 Climate Change, Extremes and Natural Hazards in Alpine Regions Research Centre CERC, Davos Dorf, Switzerland; 83 Dept of Wildlife, Fisheries and Aquaculture; Mississippi State University, Mississippi State, MS USA; 84 Institute of Plant Sciences and Oeschger Centre for Climate Change Research, University of Bern, Altenbergrain 21, Bern, Switzerland; 85 Department of Botany, University of Kashmir, Srinagar, Jammu & Kashmir India; 86 College of Forestry, Northwest A&F University, Yangling, Shaanxi China; 87 Department of Plant Agriculture, University of Guelph Ridgetown Campus, Ridgetown, ON Canada; 88 Ohio State University, Department of Evolution, Ecology, and Organismal Biology, 318 W 12th Av, Columbus, OH USA; 89 Department of Plant and Wildlife Sciences, Brigham Young University, Provo, UT USA; 90 Department of Biology and Environmental Sciences, Auburn University at Montgomery, 7061 Senator’s Drive, Montgomery, AL USA; 91 Nakagawa Experimental Forest, Hokkaido University, Otoineppu, Japan; 92 Departments Medicinal Plants and Food Technology, Samarkand Agroinnovations and Research University, Amir Temur 7, Dahbet Samarkand, Uzbekistan; 93 Department of Bioscience and Biotechnology, Fukui Prefectural University, 4-1-1 Matsuoka-Kenjojima, Eiheiji-Town, Fukui, Japan; 94 Institute of Botany, Academy of Sciences of the Republic of Uzbekistan, Tashkent, Uzbekistan; 95 Department of Botany and Genetics, Faculty of Biology and Ecology, National University of Uzbekistan named after Mirzo Ulugbek, Tashkent, Uzbekistan; 96 Department of Agrotechnology, Toraighyrov University, Lomov str. 64, Pavlodar, Kazakhstan; 97 Leibniz Institute for Horticultural Sciences (IGZ), Grossbeeren, Germany; 98 Friedrich Schiller University Jena, Jena, Germany; 99 Department of Biological Sciences, University of Alberta, Edmonton, Alberta Canada; 100 Department of Ecology and Evolutionary Biology, University of Kansas, Lawrence, KS USA; 101 State Key Laboratory of Herbage Improvement and Grassland Agro-Ecosystems, College of Ecology, Lanzhou University, Lanzhou, Gansu China; 102 State Key Laboratory for Vegetation Structure, Function and Construction (VegLab), Institute of Ecology, College of Urban and Environmental Science, Peking University, Beijing, China

**Keywords:** Invasive species, Evolutionary ecology, Plant evolution, Plant ecology

## Abstract

Rapid adaptive evolution can increase the competitive ability of invasive species in their non-native ranges. However, whether this increase is a general response and what drives it remain uncertain because the evidence is largely based on studies with limited sampling, inadequate consideration of population co-ancestry, and oversimplified estimates of competitive ability. We conduct a large-scale glasshouse experiment testing the effects of competition and drought on 100 native and 165 non-native populations of *Erigeron canadensis*, all genotyped to account for co-ancestry. Plants from non-native populations are significantly more competitive against other species than the conspecifics from native populations under both mesic and dry conditions. Genetic clustering indicates that the rapid evolution of competitive ability occurs independently in two out of four clusters in the non-native range. This advantage is present only during interspecific interactions and is absent during intraspecific competition. Repeated evolution of increased competitive ability suggests that adaptation following introduction can reshape species interactions and promote invasion success, even under future drought conditions, highlighting the importance of rapid evolution in determining the ecological impacts of invasive plants.

## Abstract (web summary)

Rapid evolution may shape the success of invasive species. This study shows that the invasive plant species *Erigeron canadensis* repeatedly evolved increased competitive ability after introduction, and that this competitive ability remains strong under drought conditions.

## Introduction

A central phenomenon of plant invasions is that invaders can have stronger competitive impacts in their non-native than in their native ranges^1–3^. This pattern is frequently attributed to rapid adaptive evolution when facing novel biotic interactions in the introduced range^4–7^, which can result in increased size and competitive ability in non-native populations relative to native ones^8^. Although, the specific mechanisms underlying such evolutionary changes can be diverse^9–11^, empirical support for increased competitive ability comes from multiple comparative common garden studies^12,13^. However, it remains unclear how widespread these evolutionary shifts are, as many previous studies have relied on limited sampling across ranges and simplified measurements of competitive ability.

Differences in competitive ability between ranges may result from unbalanced sampling across varying environments, including the comparison of non-native populations from highly competitive sites to native populations from less competitive areas^14,15^. Apparent differences between ranges may also originate from neutral, stochastic migration rather than adaptive selection^16^. If, by chance, a highly competitive genetic cluster is overrepresented in the non-native range, common garden experiments may erroneously suggest rapid evolution. However, broad, comparable sampling within ranges and accounting for population co-ancestry are rare in studies of rapid evolution, and despite increasing evidence that they are crucial^8,17^, we are not aware of any such study on the rapid evolution of competitive ability.

Competitive ability is not a single trait but a complex of traits that depends on the environmental context. It is influenced by species identity^18^, indirect effects of surrounding competitors^19,20^, neighbour density^21,22^, and whether the competitor is a conspecific or a heterospecific^23^. Yet studies measuring competition, especially in the context of rapid evolution, have often relied on pairwise comparisons with a single competitor species^24^. Moreover, competition is frequently modified by abiotic conditions: it often intensifies under low environmental stress and may weaken or even shift towards facilitation under high stress^25,26^, although the effects of resource limitation vary among systems and may strengthen some components of competition while weakening others^27,28^. This context dependency is particularly relevant as altered precipitation regimes may reshape species interactions and coexistence in plant communities^29,30^.

Additionally, it is important to consider that competition has two major components: the response to competition (tolerance to neighbours) and the effect of competition (suppression of neighbours). Competition-driven selection for tolerance to neighbours is much stronger than selection for the ability to suppress them, because strong suppressors indirectly benefit other individuals in communities, thus creating potentially detrimental indirect feedbacks^31,32^. Assessing competitive ability can therefore benefit from multi-species settings that incorporate both intra- and interspecific competition in different abiotic contexts^23,33^.

**Fig. 1:**
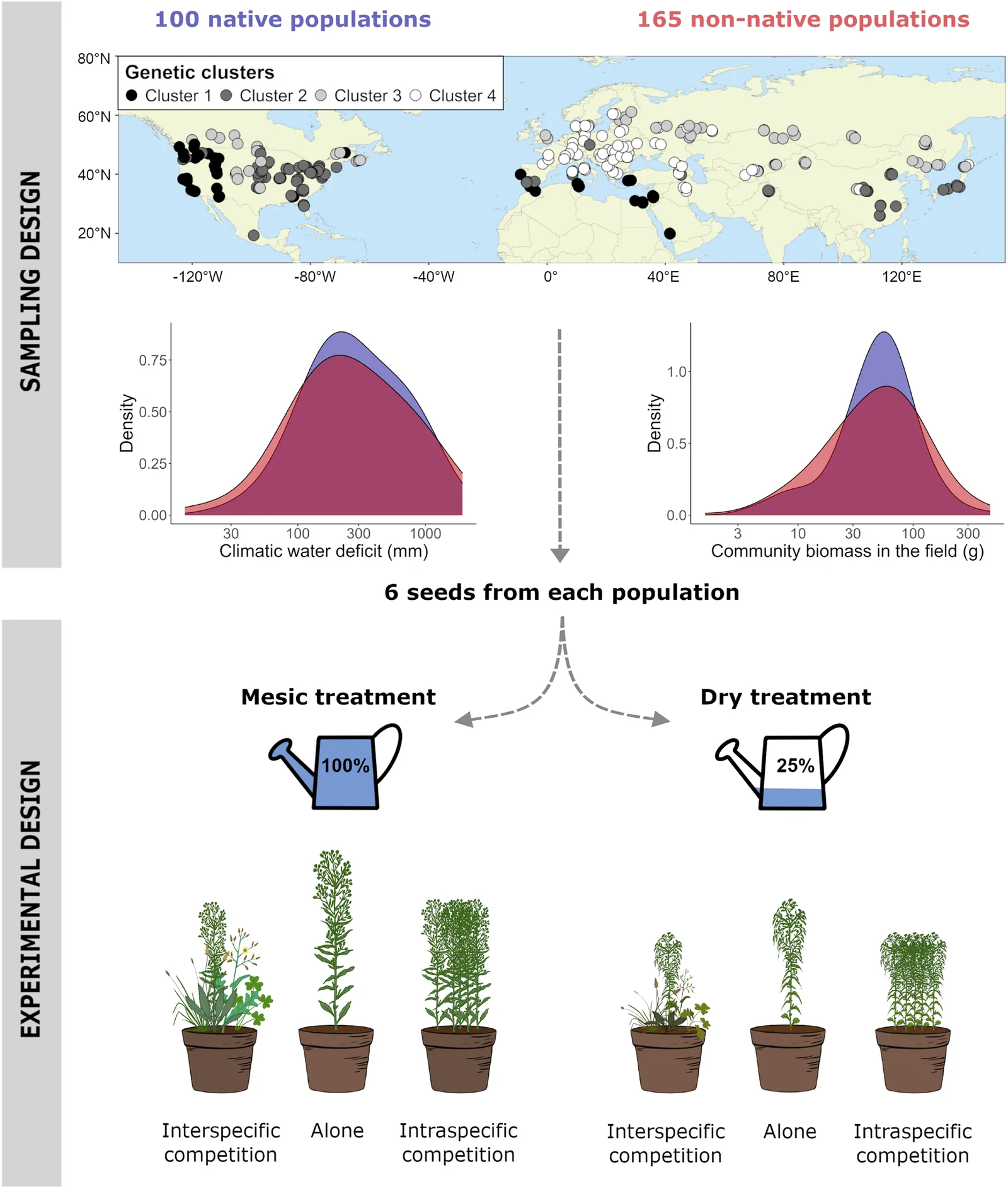
Conceptual overview of the sampling and experimental design to study rapid evolution of competitive ability in *Erigeron canadensis*. Sampling design: Geographical distribution of the sampled native (North America) and non-native (Africa and Eurasia) populations and their genetic cluster affiliation. Genetic cluster affiliation was determined using a Bayesian clustering analysis based on 4,601 neutral SNPs from ddRADseq data, which identified four optimal clusters (*K* = 4)^17^. Each population was assigned to the cluster with the highest posterior probability. Sampling covered broad and comparable environmental gradients between ranges, described by climatic water deficit (a proxy for aridity) and field community biomass (a proxy for local competition). Neither variable differed significantly between ranges when analysed using two-sample *t* tests (N = 285; climatic water deficit: t = –0.42; *P* = 0.676, and community biomass: t = 1.25; *P* = 0.108). Details of the sampled populations are provided in Supplementary Data 1. Experimental design: To quantify competitive ability under contrasting biotic and abiotic conditions, we evaluated competition and drought effects in a glasshouse experiment using a multi-species design. *Erigeron* individuals grew either alone, in intraspecific competition, with four conspecifics from the same population, or in interspecific competition, with four co-occurring species native to Eurasia and non-native to North America (*Plantago lanceolata*, *Bromus tectorum*, *Lactuca serriola*, and *Trifolium repens*). Drought was applied at two levels: pots under mesic conditions received water to maintain field capacity (estimated via gravimetric loss), while drought-treated pots received 25% of that volume. Drought significantly affected soil moisture and *Erigeron* physiology (see Supplementary Fig. 1). We estimated each population’s competitive ability by combining intrinsic growth rate (from Ancillary Experiment 1 in Supplementary Note 1) and the competition coefficients for inter- and intraspecific tolerance (see “Methods”). Similarly, we assessed *Erigeron*’s inter- and intraspecific competitive suppression on its neighbours (Supplementary Fig. 2). Source data are provided as a Source Data file.

Here, we show that increased competitive ability is associated with repeated evolutionary changes within the non-native range of a global plant invader. We conducted a glasshouse experiment with 100 native and 165 non-native populations of *Erigeron canadensis* L. (synonym: *Conyza canadensis*, Canadian horseweed, hereafter *Erigeron*), sampled across broad and comparable environmental gradients in both ranges (Fig. 1). *Erigeron* is a suitable model for studying phenotypic variation and its drivers due to its high adaptability to diverse conditions, including wide aridity gradients^34,35^. To account for population co-ancestry, we incorporated population genomic data (ddRADseq). We quantified competitive ability under mesic and dry conditions, examined variation between native and non-native ranges and among genetic clusters, and compared responses under intra- and interspecific competition.

## Results And Discussion

Our study involved 5048 pots in the main experiment (3 competition × 2 drought treatments; Fig. 1) and six ancillary experiments (Supplementary Note 1). Aboveground vegetative biomass was measured after 12 weeks, corresponding to the main vegetative growth phase of *Erigeron*^34,35^. Competition reduced *Erigeron* biomass by 63.4% (χ²_(2)_ = 3068.6; *P* < 0.001) and drought decreased biomass by 51.7% (χ²_(1)_ = 1461.9; *P* < 0.001; Supplementary Fig. 3a). Survival remained high throughout the experiment (96.8% overall) and was not significantly affected by competition, but declined by 3.2% under drought (χ²_(1)_ = 6.69; *P* = 0.009; Supplementary Fig. 3b).

### Non-native populations showed increased competitive ability in both dry and mesic conditions

**Fig. 2:**
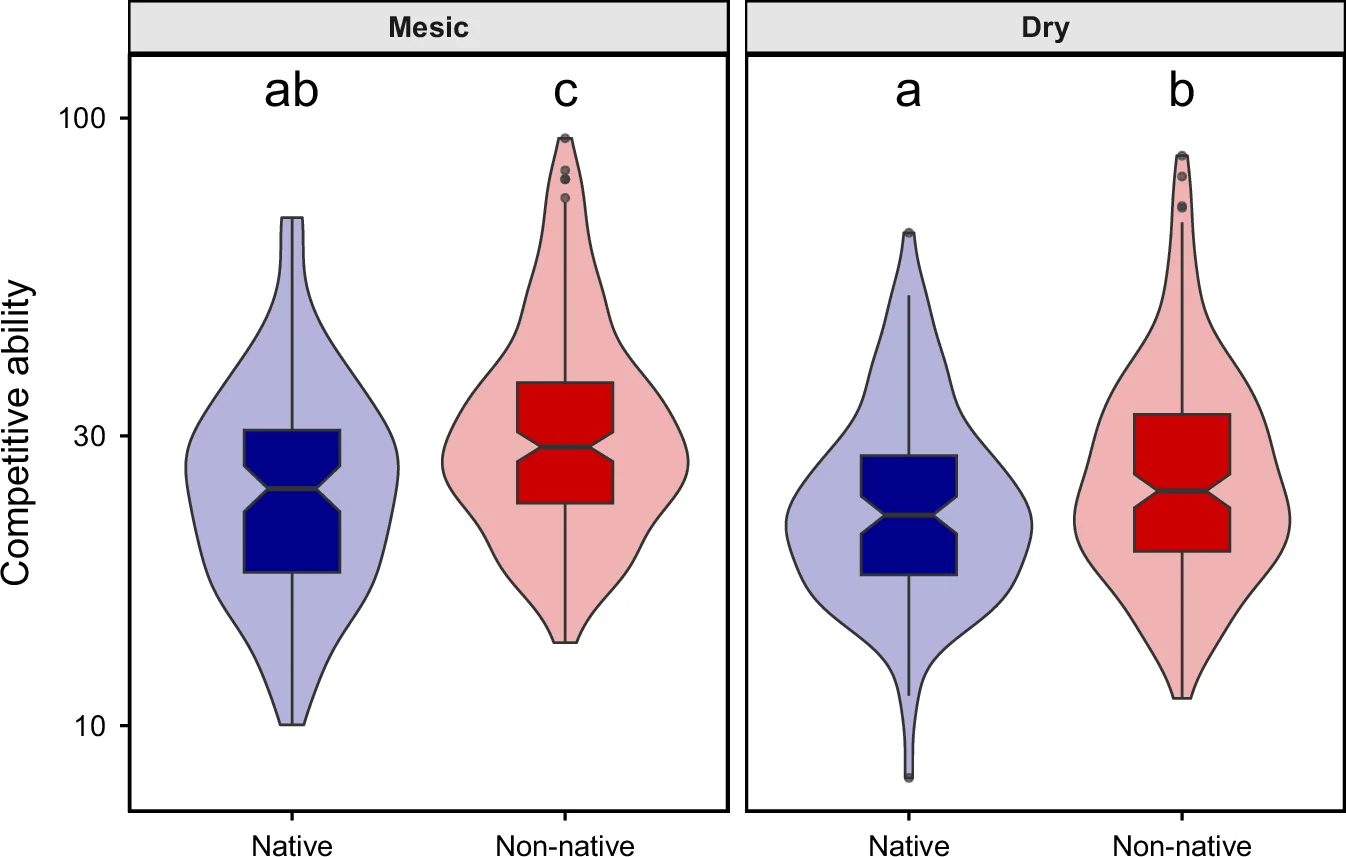
Competitive ability of *Erigeron canadensis* sourced from non-native (red; *n* = 165) and native ranges (blue; *n* = 100) in mesic and dry treatments. Competitive ability was calculated as the intrinsic growth rate divided by the geometric mean of intraspecific and interspecific competitive tolerance. Across both watering treatments, non-native populations showed significantly greater competitive ability than native populations (χ²_(1)_ = 13.89, *P* < 0.001). Drought stress reduced the competitive ability of *Erigeron* (χ²_(1)_ = 16.66; *P* < 0.001), but non-native populations showed higher competitive ability than conspecifics from the native range in both treatments. Models included climatic water deficit and field community biomass as covariates and accounted for population co-ancestry using a pedigree mixed-effects model (Supplementary Table 1). Lowercase letters indicate Tukey-adjusted pairwise comparisons. Competitive ability was calculated from population mean values separately for the mesic and dry treatments, with each population constituting one independent biological replicate. Violin plots show the smoothed data distribution. Boxplots show the median (centre line), interquartile range (box), whiskers extending to the most extreme values within 1.5 × the interquartile range, and outliers plotted individually. Source data are provided as a Source Data file.

Competitive ability was calculated following Hart et al.^36^, integrating competitive tolerance and intrinsic growth rate of the focal *Erigeron*, with aboveground biomass used as a performance proxy. An ancillary experiment showed a strong positive relationship between biomass at harvest (between 0.3 and 3 g) and fecundity (between 100 and 500 capitula), indicating that higher competitive ability also reflects increased reproductive output (Supplementary Fig. 4; Ancillary Experiment 2 in Supplementary Note 1). Across both watering treatments combined, *Erigeron* populations sourced from the non-native range had 19.2% higher competitive ability than conspecifics from the native range (χ^2^_(1)_ = 13.89; *P* < 0.001; Fig. 2). This effect size is consistent with those reported in studies linking invasion to enhanced competitive performance (reviewed by Callaway et al. ^37^).

Multiple mechanisms may explain this pattern. One explanation is the EICA hypothesis, which proposes that escape from specialist natural enemies may favour the reallocation of resources from defence to growth and reproduction, thereby increasing competitive ability^10,37^. However, Abhilasha and Joshi^38^ reported that *Erigeron* plants from non-native populations were larger without evidence for reduced herbivore defences. Similarly, Sheng et al. ^39^ found no evidence of increased susceptibility for belowground fungal pathogens. Both studies suggest that processes other than EICA might contribute to the rapid evolution of increased competitive ability. Alternatively, competitive advantages may arise from increased investment in allelopathy by invasive populations^40^. There is evidence for allelopathic effects exerted by *Erigeron*^41^, but it is unclear whether allelopathic effects differ between native and non-native populations^38^. Other research suggests that novel selection pressures from different abiotic conditions^42,43^ or unique competitive or mutualistic interactions^2^ may lead to increased competitive ability in non-native ranges. For example, Sheng et al^44^. reported that non-native populations were associated with mycorrhizal fungal taxa that may be more beneficial, potentially enhancing growth and competitive performance.

Across native and non-native populations combined, drought reduced competitive ability by 12.9% compared with mesic conditions (χ²_(1)_ = 16.66; *P* < 0.001; Fig. 2). This result aligns with the stress-gradient hypothesis^26^, which states that increasing environmental stress can decrease competitive intensity or frequency^45,46^. Drought also reshapes competitive hierarchies through species-specific responses to drought^47^. These dynamics are particularly relevant during invasions, when stressors may influence both competitive processes and community assembly^48^. However, the competitive abilities of our populations under mesic and dry conditions were positively correlated with one another (Supplementary Fig. 5), although this relationship showed considerable variability among populations. Importantly, there was no significant interaction between range (native vs. non-native) and drought treatment (mesic vs. dry) (χ²_(1)_ = 2.69; *P* = 0.10). In other words, plants from the non-native range consistently exhibited higher competitive ability than conspecifics from the native range, regardless of drought stress. These results provide no evidence for a trade-off between stress tolerance and competitive ability^49^. Instead, our study suggests that directional selection in the non-native range may favour genotypes with strong competitive ability independently of traits related to stress tolerance, as also observed in other invasive species such as *Centaurea stoebe*^50^. Because drought does not appear to be a primary driver of the rapid evolution of competitiveness, our findings support the view that shifts in biotic interactions can exert a stronger influence on natural selection than changes in abiotic conditions^51,52^.

### The role of population co-ancestry in variation in competitive ability

**Fig. 3:**
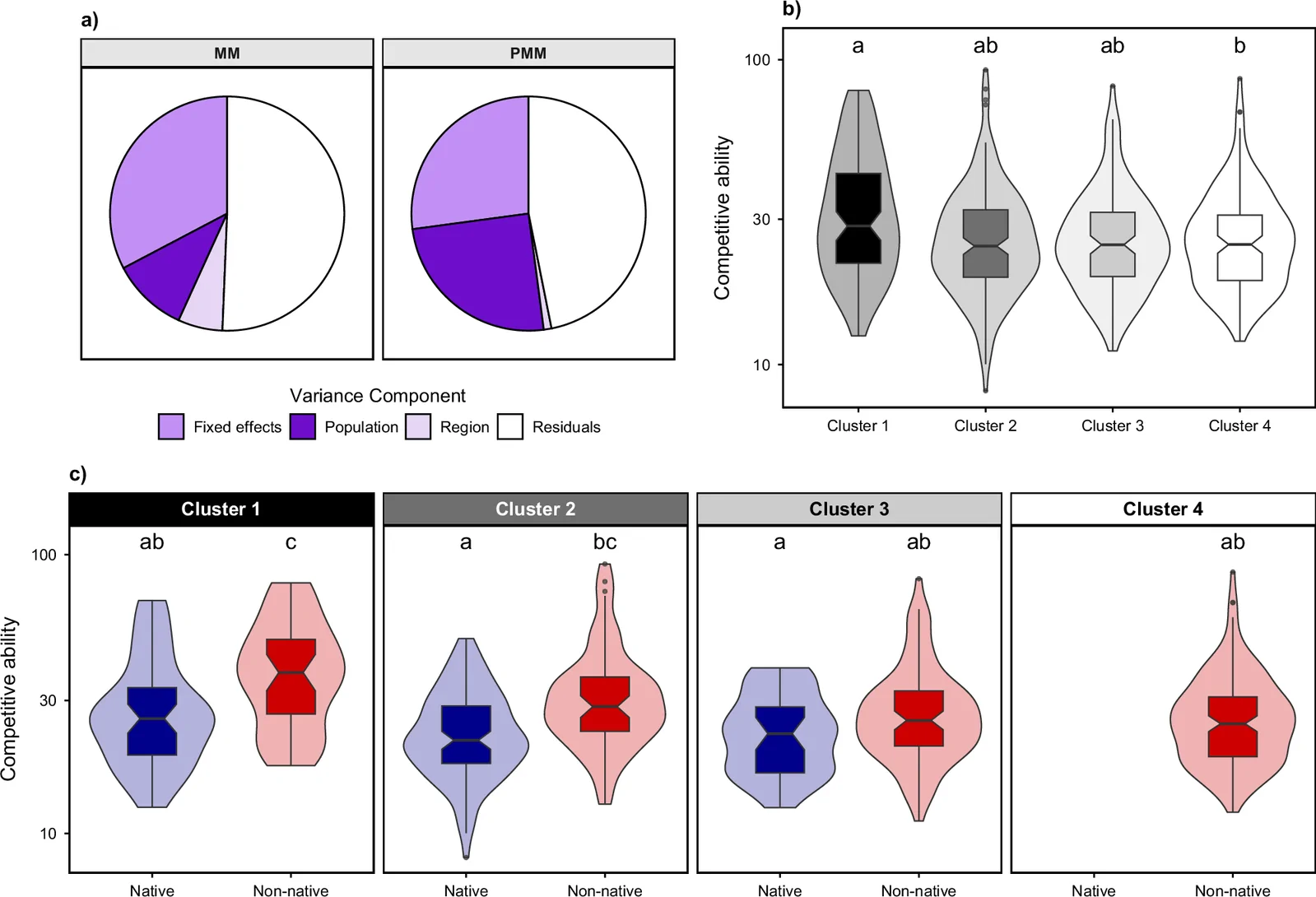
Effect of population co-ancestry on competitive ability in *Erigeron canadensis*. Panels a–c present complementary analyses linking genetic structure to variation in competitive ability. **a** Pie charts showing the proportion of variance explained by different components of a linear mixed-effects model (MM) and its corresponding pedigree mixed-effects model (PMM). The slices represent unexplained variance (residuals), variance explained by the random effects of population and region, and the variance explained by all fixed effects combined (range, drought treatment, climatic water deficit, and field community biomass; Supplementary Table 1). Accounting for population co-ancestry in the PMM increased the variance explained by the population random effect from 10.5% to 25.0%. **b** Competitive ability differed significantly among the four genetic clusters (*n* = 265; χ²_(3)_ = 18.05; *P* < 0.001). Additional analyses treating cluster assignment probabilities as continuous variables produced similar patterns (Supplementary Fig. 9). **c** Competitive ability differed significantly between native (blue) and non-native (red) populations in Cluster 1 (black; *n* = 65; t = 4.122; *P* < 0.001) and Cluster 2 (dark grey; *n* = 45; t = 4.778; *P* < 0.001), but not in Cluster 3 (light grey; *n* = 71; t = 2.104; *P* = 0.334), as indicated by Tukey-adjusted pairwise comparisons. Cluster 4 (white; *n* = 84) occurred exclusively in the non-native range and was therefore not included in range comparisons. The response of competitive ability to drought across the four clusters is shown in Supplementary Fig. 8. Model estimates for the linear mixed-effects models in panels (**b** and **c**) are presented in Supplementary Table 2. In panels **b** and **c**, competitive ability was calculated from population mean values separately for the mesic and dry treatments; each population constituted one independent biological replicate. Violin plots show the smoothed data distribution. Boxplots show the median (centre line), interquartile range (box), whiskers extending to the most extreme values within 1.5 × the interquartile range, and outliers plotted individually. Source data are provided as a Source Data file.

To account for population co-ancestry, which is only occasionally incorporated into eco-evolutionary studies, we used pedigree mixed-effects models (PMMs^53^). We constructed a co-ancestry matrix, based on 4601 neutral SNPs from ddRADseq data and incorporated it as a random effect in our PMMs. Including this matrix more than doubled the variance explained by population, from 10.5% in regular mixed-effects models (MMs) to 25.0% in PMMs (Fig. 3a), and reduced the residual variance by 7.5%. The PMMs also had a lower AIC than the MMs (495.4 vs. 508.6). The improved model fit in PMMs indicates that co-ancestry captures additional biologically meaningful variation, consistent with earlier work on phenotypic variation in *Erigeron*^35^. When models were run separately for native and non-native populations, the inclusion of the co-ancestry matrix increased the variance explained by the population term more in the non-native (27.5%) than in the native range (18.2%). This finding suggests that in non-native ranges, recent founder effects and admixture can strongly affect population demography, making co-ancestry a more powerful predictor of trait variation than in the native range, where historical population structure dominates^54^.

To further disentangle the effects of selection from purely demographic processes, we performed a Bayesian clustering analysis based on the same SNP dataset and asked whether differences in competitive ability aligned with ancestry-defined groups (Fig. 1 and Supplementary Fig. 6 and 7). Genetic cluster affiliation had a significant effect on competitive ability (χ²_(3)_ = 18.05; *P* < 0.001; Fig. 3b and Supplementary Table 2), confirming that intraspecific variation in competitive ability can be strongly associated with the neutral genetic relatedness of populations^53,55^. Furthermore, increased competitive ability in the non-native range was observed in two independent genetic clusters: Cluster 1, where non-native populations from Mediterranean regions had 37.1% greater competitive ability than native populations from western North America, and Cluster 2, where eastern Asian populations had 34.2% greater competitive ability than eastern North American populations (Fig. 3c). These patterns suggest that increased competitive ability may evolve within genetic clusters rather than solely reflecting the overrepresentation of a single competitive lineage^56^. This finding is also consistent with the view that evolutionary changes associated with invasiveness can arise repeatedly in independent genetic clusters^57,58^. Given the predominantly selfing mating system of *Erigeron* and the largely non-overlapping geographic distributions of Clusters 1 and 2, extensive gene flow between these clusters is unlikely, supporting their evolutionary independence.

In Cluster 3, we found no evidence of increased competitive ability in non-native populations. These populations occur near the northern edge of the species’ distribution, and adaptation to harsher climatic conditions (e.g., shorter growing seasons) may play a stronger role than competition in shaping their performance. Cluster 4 showed comparatively low competitive ability and occurred as a dominant genetic cluster only in the non-native range; therefore, evolutionary changes within this cluster cannot be directly assessed. Importantly, non-native populations belonging to this less competitive cluster were included in the overall comparison between native and non-native ranges (Fig. 2). Nevertheless, non-native populations still exhibited significantly greater competitive ability than native populations overall, making the estimated range effect conservative. The distribution of Cluster 4 may reflect the complex invasion history of *Erigeron*, including multiple introductions with subsequent population admixture and rapid post-introduction evolution^59,60^. Such genetic divergence after introduction may have been facilitated by its annual life cycle and the long invasion history, as *Erigeron* has been established in Europe since the 17th century^61^.

When comparing the clusters between the two watering conditions, Cluster 1 showed significantly higher competitive ability under mesic than dry conditions, whereas the other clusters exhibited similar but non-significant trends (Supplementary Fig. 8). This pattern may suggest that populations belonging to Cluster 1 increase competitive performance primarily under favourable conditions rather than maintaining consistently high competitiveness across environments^62^.

### Interspecific rather than intraspecific interactions drive the evolution of competitive suppression and tolerance

To identify the drivers of increased competitive ability, we tested how the competitive tolerance and competitive suppression of *Erigeron* differed between interspecific and intraspecific competition, and how these differences interacted with range and drought treatment (for model details, see Supplementary Table 3). Both traits were quantified as competition coefficients: competitive tolerance describes the biomass response of the focal *Erigeron* to competition from neighbouring plants (see Fig. 1), whereas competitive suppression describes *Erigeron*’s effect on the biomass of the competitor community (see Supplementary Fig. 2).

**Fig. 4:**
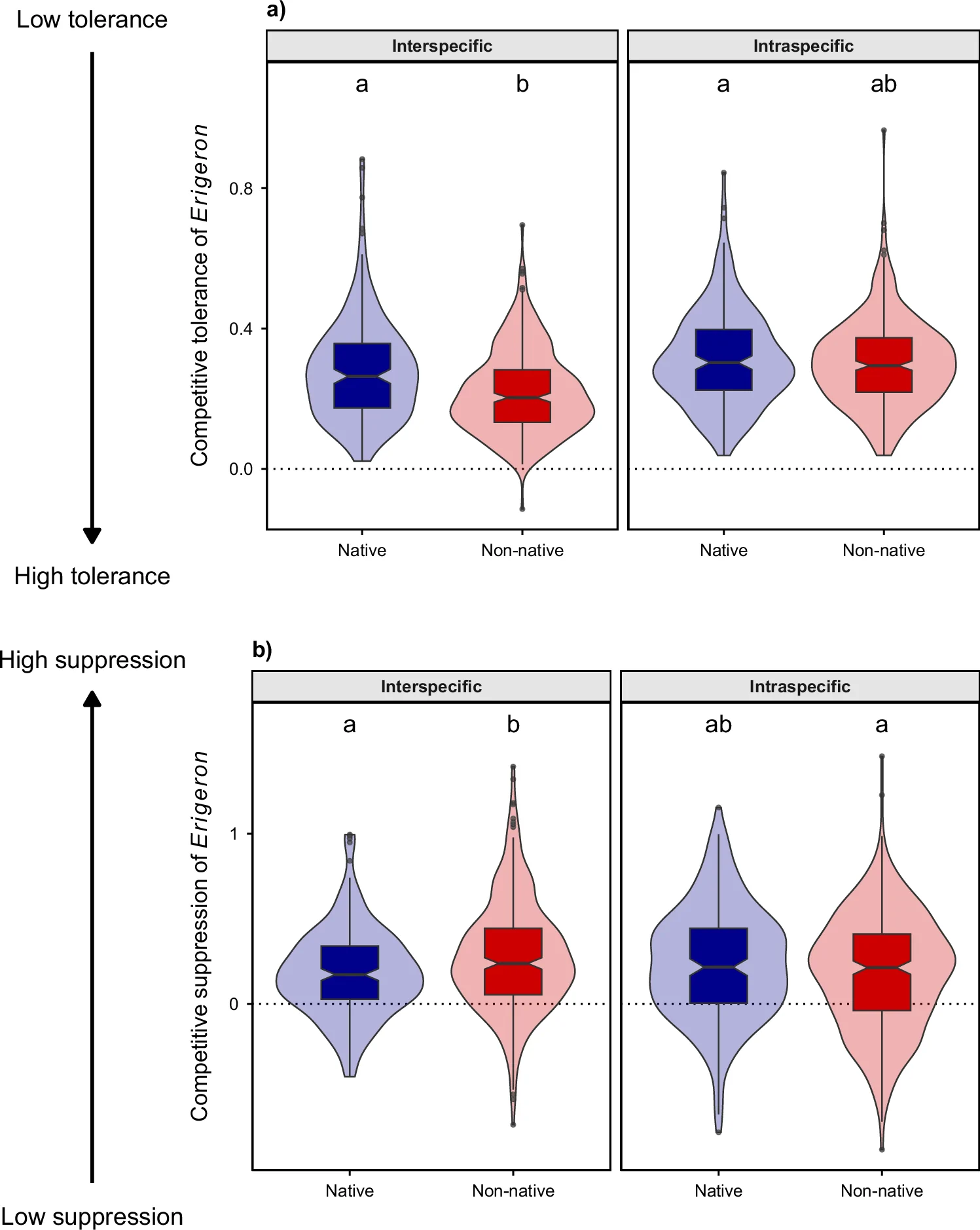
Increased competitive ability of non-native *Erigeron canadensis* is explained by interspecific rather than intraspecific competitive tolerance and suppression. **a** Competitive tolerance of *Erigeron* under interspecific and intraspecific competition. A significant interaction between range and competition type was detected (χ²_(1)_ = 9.21, *P* = 0.002). Non-native populations (*n* = 165) showed significantly higher tolerance than native populations (*n* = 100) under interspecific competition, but no such difference was found for intraspecific competition. **b** Competitive suppression of neighbouring plants by *Erigeron* under interspecific and intraspecific competition. A significant interaction between range and competition type was detected (χ²_(1) =_ 13.80, *P* < 0.001). Non-native populations (*n* = 165) exerted a significantly stronger competitive suppression on interspecific communities than native populations (*n* = 100), with no such difference for intraspecific communities. In both panels, horizontal dotted lines at 0 indicate the threshold between competition (y > 0) and facilitation (y < 0). Arrows visualise the direction of change: an increase in competitive tolerance reflects a stronger effect experienced by *Erigeron* (higher values = lower tolerance), whereas an increase in competitive suppression reflects a greater effect on competitor communities (higher values = higher suppression). The models for both panels also included the interaction between the drought treatment and competition type and accounted for population co-ancestry using a pedigree mixed-effects model (Supplementary Table 3; Supplementary Fig. 10). Competitive tolerance and suppression were positively correlated under both interspecific and intraspecific competition (Supplementary Fig. 11), indicating that populations with high competitive tolerance also tended to exhibit high competitive suppression. Lowercase letters indicate Tukey-adjusted pairwise comparisons. In both panels, competitive tolerance and suppression were calculated from population mean values separately for the mesic and dry treatments; each population constituted one independent biological replicate. Violin plots show the smoothed data distribution. Boxplots show the median (centre line), interquartile range (box), whiskers extending to the most extreme values within 1.5 × the interquartile range, and outliers plotted individually. Source data are provided as a Source Data file.

Overall, intraspecific competition reduced *Erigeron* biomass more strongly than interspecific competition. At the same time, *Erigeron* exerted weaker suppressive effects on conspecifics than on heterospecific competitors. This pattern is consistent with reviews showing that intraspecific competition is typically stronger because of greater niche overlap, whereas interspecific competition is often alleviated by niche differentiation^23^. The most notable pattern emerged when we examined interactions with range. We found significant interactions between range and competition type for both competitive tolerance (χ²_(1)_ = 9.21; *P* = 0.002; Fig. 4a) and suppression (χ²_(1)_ = 13.80; *P* < 0.001; Fig. 4b). Under interspecific competition, non-native *Erigeron* populations showed 28.4% greater tolerance and 38.0% greater suppression compared with native populations. In contrast, no such differences were found between ranges under intraspecific competition. These findings suggest that evolutionary shifts in competitive ability are primarily shaped by interspecific rather than intraspecific interactions. The similarity in intraspecific competition between native and non-native ranges likely reflects comparable selection pressures, as *Erigeron* competes with conspecifics in both ranges. In contrast, interspecific competition likely involves markedly different species compositions and interaction strengths in the two ranges, leading to divergent selection pressures^2,63^.

Species identity influences competitive outcomes^64,65^, and we only tested the effects of four different competitor species. However, our multispecies design captured a broader range of interactions than pairwise approaches and more closely reflects natural community contexts^20^. Still, glasshouse conditions simplify natural environments^66^, and future studies should test whether these patterns persist in the field and how neighbour identity shapes competitive mechanisms.

The parallel patterns in competitive tolerance and suppression contrast with previous studies reporting divergent selective pressures on these facets of competition^20,32,67^. However, the increased competitive suppression of non-native populations aligns with findings that invasive species often allocate more resources to traits that suppress neighbours^37,68,69^. Such genotypes may also be highly tolerant when traits like rapid growth or large size confer advantages in both suppressing neighbours and withstanding competition. In fact, populations with the highest growth rates tended to be both the most tolerant and the most effective competitors, likely because they dominated their neighbours before those neighbours could suppress them^70^. The absence of a trade-off between tolerance and suppression may therefore reflect evolution towards general-purpose or high-performance genotypes^71^.

### Ecological and evolutionary implications

Our study suggests that rapid adaptive evolution contributes to increased competitive ability in non-native ranges. A common limitation of previous research has been the underestimation of within-range variation and the omission of population co-ancestry^8,14,15^. We found that accounting for co-ancestry explained a substantial proportion of variation in competitive ability, highlighting the value of incorporating population co-ancestry into future studies of rapid evolution in invasive species^35^. Genetic cluster analyses revealed that the evolution of increased competitive ability has arisen multiple times within the non-native range, supporting the view that this phenomenon may be widespread among invasive species^72,73^. Interspecific rather than intraspecific interactions drive increased competitive ability in non-native *Erigeron*, emphasising the essential role of altered biotic interactions in promoting rapid evolution^51,52^.

Increased competitive ability may have facilitated the expansion of *Erigeron* across its large non-native range and may help explain why invaders suppress native diversity much more in their non-native ranges^2,3,12^. Because we found no evidence for a trade-off between competitive ability and drought tolerance, invasive populations may maintain these competitive advantages under future drought scenarios^34,74,75^. *Erigeron* is known for its exceptional capacity for rapid evolution^35,44,76^, but other invaders may also increase their invasiveness over time, particularly where native communities lack co-evolutionary history with the invader^2^. Because *Erigeron* is an annual species with a short generation time, rapid evolutionary responses may be more readily detectable than in longer-lived species^35^. Nevertheless, the evolutionary mechanisms underlying adaptation are not unique to annuals, and similar processes are expected to occur in perennial or clonally reproducing invaders over longer timescales. Further studies with multifactorial experimental designs are needed to clarify the role of rapid evolution in shaping invasion dynamics and its impact on global biodiversity^8^.

## Methods

### Study species

*Erigeron canadensis* is an annual herb belonging to the Asteraceae family. The species is native to North and Central America but was introduced to Europe in the 17th century, from where it rapidly expanded across temperate Eurasia and became common in Central Europe by the mid-18th century^61^. Its spread is likely linked to multiple unintentional introductions followed by rapid range expansion. Today, *Erigeron* has a nearly global distribution, particularly throughout the Northern Hemisphere, where it occurs across a wide range of environmental conditions. As a disturbance-adapted species, it often colonises open, ruderal habitats^77^. However, in several parts of its introduced range, it also colonises more competitive environments and is considered invasive due to its rapid spread and impacts on native plant communities^2^.

*Erigeron* exhibits several characteristics that make it suitable for studying rapid evolution in invasive species^8,34,35^. The annual life cycle of *Erigeron* may facilitate rapid evolutionary responses^78^. It was the first eudicot weed reported to evolve glyphosate resistance^79^, which has occurred multiple times and independently in different locations^76^. The species is predominantly self-pollinating, which is often thought to reduce the effectiveness of selection, but selection can still be strong in selfing species, especially for strongly selected alleles^80,81^. Selfing can rapidly expose new mutations in homozygous form, allowing beneficial recessive alleles to experience immediate and strong selection^35^. Occasional outcrossing and repeated introductions may also help maintain genetic variation in the invasive range, facilitating evolutionary responses to novel environments^82^. Moreover, rapid evolutionary change is not necessarily dependent on outcrossing, as large genomic effects may also arise from mechanisms such as transposable element activity or structural variation^83^.

Plant material was collected from publicly accessible sites in accordance with applicable local regulations. No collection permits were required for the sites from which *Erigeron* was sampled. Seed material was transferred following the applicable import requirements. At the time of import, no import permit was required for the *Erigeron* seed material used in this study.

### Sampled populations

We collected seeds from 108 native and 176 non-native *Erigeron* populations from 21 distinct geographical regions. Our sampling covered most of the species’ distribution in the Northern Hemisphere (Supplementary Data 1; Fig. 1) and spanned a broad, representative gradient of the environment, by capturing 92% of the climatic niche in the native and 86% in the non-native ranges (Supplementary Note 2). During the glasshouse experiment, 19 populations were excluded due to low germination or seedling survival (Supplementary Data 1), leaving 265 populations for the statistical analyses.

From each population, we collected seeds from a single individual. The individual was chosen to represent the population as well as possible, excluding damaged, small, and poorly developed plants. Sampling a single individual may underestimate within-population genetic variation. However, previous studies have shown that *Erigeron* exhibits relatively low genetic variation within populations^35,44^. Therefore, we prioritised sampling a larger number of populations rather than increasing within-population replication, thereby maximising geographic coverage and our ability to detect large-scale evolutionary differentiation across the species’ range.

To account for variation in the competitive regime across populations, we collected, dried, and measured community biomass (including *Erigeron*) from five 0.25 m^2^ plots following Sheng et al. ^44^. To estimate the local aridity regime experienced by *Erigeron* in the field, we downloaded climatic water deficit data for each population from the TerraClimate^84^ dataset at a ~ 4 km spatial resolution using annual mean values from 1958 to 2023. This measure captures the local aridity conditions of the source populations by quantifying the unmet evaporative demand. In particular, it estimates the difference between actual and potential evapotranspiration, so that the climatic water deficit represents the missing water that would be required to maximise plant growth^85^.

### Experimental design

Our glasshouse experiment had a full factorial design for the drought × competition treatment combination (Fig. 1). The competition treatment included three setups: 1) pots with one *Erigeron* individual without competitors, 2) pots with one focal *Erigeron* and four competitor individuals, each from a different species (i.e., interspecific competition), and 3) pots with one focal *Erigeron* and four competitor *Erigeron* individuals from the same population (i.e., intraspecific competition). The drought treatment included pots with mesic and dry conditions (see below for details). In total, 3408 pots were installed (284 populations × 2 replicates per population × 2 drought treatments × 3 competition treatment). In addition, we grew an extra 1640 pots to perform six ancillary experiments (see below for details).

### Competitor selection

To avoid overestimating competitive effects in pairwise interactions^20,23^, our interspecific competition treatment followed a multispecies approach. The four competitor species were selected based on three criteria. First, candidate species were identified based on their frequent co-occurrence with *Erigeron* in vegetation surveys conducted during field sampling across both the native and non-native ranges. Second, competitor species were chosen with contrasting biogeographic histories relative to *Erigeron*. While *Erigeron* is native to North America and introduced to Eurasia, the selected competitors are native to Eurasia and introduced to North America. Thus, interactions between these species and *Erigeron* have occurred only since recent historical introductions rather than through long-term co-evolution. Third, the selected competitors represent different phylogenetic and functional groups to reduce phylogeny-specific competition effects.

Based on these criteria, we identified eleven candidate species. To ensure that the candidate species would compete with *Erigeron* during early growth stages, we grew individual seedlings of all species. In the first four weeks, most candidate species produced greater biomass than *Erigeron*, confirming their ability to exert competitive pressure during early development (Supplementary Fig. 12). We selected *Lactuca serriola, Plantago lanceolata, Trifolium repens*, and *Bromus tectorum* based on germination rates and seedling survival. Seeds of all competitor species were obtained from commercial seed suppliers (Rieger-Hofmann; WeberSeeds).

### Experimental set up

Our glasshouse experiments (main experiment and the six ancillary experiments) were set up in spring 2021, in Butte, Montana, USA, using 1450 mL pots (11 × 11 × 12 cm). The soil consisted of compost, topsoil (from a ruderal site), and sand in a 1:1:1 ratio and was thoroughly mixed, using a high-volume mixer. Because this mixture contained two-thirds nutrient-rich soil components (compost and topsoil), plant growth was not strongly limited by nutrient availability. The soil mixture was purchased from Wagner Nursery (Butte, Montana, USA) and was not sterilised in order to avoid structural changes to soil properties. No additional fertiliser was applied during the experiment. The pots were filled individually with 1025 g (± 10 g) of well-watered soil mixture. Once the pots were filled with soil, 15 pots were placed in one plant growing tray.

We used field-sampled seeds for our experiments. The environment experienced by maternal plants in the field can influence offspring performance because resources provided by the mother contribute to seedling growth^86^. To reduce potentially confounding effects of seed resources on competitive ability, we selected seedlings of similar size from each population to be included in the experiment.

For the main experiment, the focal *Erigeron* individual was always sown in the centre of the pot, while the competitors (both inter- and intraspecific) were sown near the corners. Ten to twenty seeds per species and position were sown in the alone, interspecific, and intraspecific combinations. After sowing, pots were randomised evenly in the trays and in the glasshouse. No significant patterns in germination among populations or treatments were observed. After successful germination, individuals were thinned to have one *Erigeron* individual in the alone pots, five *Erigeron* individuals in the intraspecific pots, and one *Erigeron* plus one individual from each competitor species in the interspecific pots. The position of individual pots within trays (every Monday), the distribution of pots across trays (every Wednesday), and the position of trays within the glasshouse (every Friday) were randomised throughout the experiment. Pots were watered equally and regularly with a sprayer until transition to either the mesic or dry treatment after six weeks.

Glasshouse temperatures were maintained between 20–25 °C during the day and 10–15 °C at night during the first month of the experiment. Because the experiment started in mid-March when natural light availability and outside temperatures were still limited, supplemental lighting (334 µmol m⁻² s⁻¹) was provided between 7:00–9:00 and 16:00–18:00 during this period. From mid-April onward, plants were grown under ambient glasshouse temperature and natural daylight conditions without additional lighting or temperature control. From June to July, active ventilation was used to increase air circulation through the glasshouse roof openings, thereby preventing prolonged temperatures above 30 °C. Relative air humidity in the glasshouse was not actively controlled.

At that time, we estimated the initial focal *Erigeron* biomass of seedlings using a non-invasive, photography-based area estimation. To calibrate this method, we grew an additional 100 plants in separate pots and harvested them for biomass after six weeks (destructive sampling) to generate regression coefficients (Ancillary Experiment 1 in Supplementary Note 1; Supplementary Fig. 13).

### Drought treatment and data collection

At the start of the drought treatment, all pots from the mesic and dry treatments were watered to field capacity. We measured 360 randomly chosen pots (60 populations from each of the six competition/drought treatment combinations) to record pot weight and soil moisture using a Theta Probe Type ML2x Soil Moisture Device (Delta-T Devices, Cambridge, UK). The measurements confirmed comparable starting conditions across competition and drought treatments for soil moisture (mean = 36.44%; SD = 2.69%; first measurement point in Supplementary Fig. 1a) and pot weight (mean = 1260.53 g; SD = 26.28 g; first measurement point in Supplementary Fig. 1b). The mean pot weight at field capacity was then used as a reference during the experiment to apply a gravimetric drought treatment, following the protocol of Nagy et al. ^34^. In particular, two days after initiating the drought treatment, 180 pots from the mesic treatment (60 from each of the three competition treatments) were weighed to calculate their average water loss (average weight at field capacity minus current average weight). Fifteen times this amount of water in mL was applied to each tray of the mesic treatment (corresponding to the 15 pots per tray) using measuring cups. Trays from the dry treatment received 25% of this volume of water. This procedure was repeated on Mondays, Wednesdays, and Fridays throughout the experiment by determining the average water loss each day.

During the 12-week drought treatment (May–July), a total of 28.9 L of water was applied to each mesic tray and 7.24 L to each dry tray. This corresponds to a total precipitation of 159.5 mm and 39.9 mm for the mesic and dry treatments, respectively. These values match the mean precipitation regimes *Erigeron* experiences during May–July in the following sampled populations: FRA-BAY, an oceanic Aquitanian French population (160.12 mm), and TUN-BIR, an arid eastern-coastal Tunisian population (37.13 mm; values based on monthly data obtained from TerraClimate^84^).

The intensity of the drought treatment was monitored throughout the experiment in Ancillary Experiment 3 (Supplementary Note 1), using soil water content and pot weight measurements taken every two weeks from 360 pots (the same as used for the initial measurement described above). This setup allowed us to track drought stress over time. In addition, stomatal conductance and δ¹³C isotope ratios were measured in 120 randomly selected individuals from the same ancillary experiment to assess the physiological impact of the drought treatment (Ancillary Experiment 3 in Supplementary Note 1; results are shown in Supplementary Fig. 1).

### Survival and harvest

The survival of the focal *Erigeron* individuals was recorded on Mondays, Wednesdays and Fridays throughout the experiment. All plants were harvested 12 weeks after the drought treatment was initiated (18 weeks after sowing). Aboveground biomass of the focal *Erigeron* and competitor communities was collected and dried for three days at 60 °C to determine dry biomass. Biomass can be used to calculate growth rate, but may not always represent a suitable predictor of fecundity. However, many *Erigeron* individuals had not developed their final number of capitula at the time of harvest. To test whether our biomass estimates correlate with fecundity, we conducted an ancillary experiment with 200 pots (Ancillary Experiment 2 in Supplementary Note 1). We found a highly significant positive relationship between biomass at the time of harvest and number of capitula at full bloom (χ^2^_(1)_ = 59.613; *P* < 0.001; R^2^ = 0.452; Supplementary Fig. 4), supporting the use of biomass as a proxy for competitive ability and its relevance to the reproductive fitness of *Erigeron* across the different competition treatments.

### Quantifying competitive ability

To quantify the competitive ability of the investigated *Erigeron* populations under mesic and dry conditions, we calculated a competitive ability index adapted from Hart et al.^36^:

$$\frac{{\lambda }_{i}}{\sqrt{{\alpha }_{{ii}}{\alpha }_{{ij}}}}$$

Equation (1) quantifies competitive ability of *Erigeron*. λ_i_ represents the intrinsic growth rate in the absence of competitors. α refers to the competitive tolerance where α_ii_ and α_ij_ correspond to the per capita effects of intraspecific and interspecific competition on *Erigeron*, respectively, based on the Beverton–Holt competition model^87^. The intrinsic growth rate was calculated as the log_e_-transformed difference between the focal *Erigeron* biomass at harvest and its estimated initial biomass, i.e., the predicted seedling biomass before initiating the drought treatment (see Ancillary Experiment 1 in Supplementary Note 1; Supplementary Fig. 13). Competitive tolerances (α_ii_ and α_ij_) were calculated as the quotients of the difference between the log_e_-transformed biomasses in the alone and competition treatments, divided by the number of competitors. All indices were calculated using population mean data and were calculated separately for the dry and mesic treatments.

To quantify the competitive suppression exerted by *Erigeron*, we estimated the extent to which a focal *Erigeron* individual suppressed the growth of its surrounding inter- or intraspecific competitor communities. To do so, we grew an additional 240 pots of competitor communities growing alone. These extra pots included 120 pots with the four interspecific competitors and 120 pots with four intraspecific *Erigeron* competitors, both without the focal *Erigeron* in the centre. This allowed us to calculate the quotient (α) between the log_e_-transformed biomasses of the communities grown without versus with a focal *Erigeron* individual (Ancillary Experiment 4 in Supplementary Note 1, Supplementary Fig. 2).

Plant-plant interactions can be highly density-dependent^22^. As such, simple comparisons of performance with and without competitors, as we did, may not necessarily reflect the complexity of species interactions, specifically if there are non-linear relationships between competitor density and the effects of competition^21^. However, identifying density-dependent relationships requires a large number of replications within populations (i.e., several levels of density). Our main focus was to quantify the drivers of rapid evolution in competitive ability across two drought treatments and broad biogeographical scales, not to estimate precise values of competitive ability of specific *Erigeron* populations. Our experimental design therefore aimed to include the highest number of populations by using simple comparisons rather than several replications within populations. To explore potential limitations of our results related to density-dependent relationships, we conducted an ancillary experiment with 540 pots from six populations facing 15 different density treatments (Ancillary Experiment 5 in Supplementary Note 1). For each population × treatment combination, individual linear models were fitted, from which the model estimates (intercept and slope of the biomass - density relationship) were used to estimate λ, α_ii_, and α_jj_. From these variables, we calculated density-controlled competitive ability (Equation 1). We then fitted linear models to test the relationship between the estimates of competitive ability calculated in the density-dependent approach and the simplified pairwise approach. There were significant correlations between both experimental approaches for the overall competitive ability (F_(1,10)_ = 9.481; *P* = 0.012; R^2^ = 0.438; Supplementary Fig. 14a), the interspecific competitive tolerance (F_(1,10)_ = 13.780; *P* = 0.017; R^2^ = 0.448; Supplementary Fig. 14b), and the intraspecific competitive tolerance (F_(1,10)_ = 9.048; *P* = 0.031; R^2^ = 0.386; Supplementary Fig. 14c). This means that the results estimated in the main experiment (i.e., measured at only two densities: with and without competition) provide comparable results as if measured through a density gradient approach. In other words, the density of competitors does not generally shift competitive outcomes, allowing the usage of our simplified estimate to describe variation in competitive ability across the *Erigeron* populations.

Most studies that test the competitive ability of a focal species overlook the need to compare it with the competitive ability of the chosen competitor species^24^. This omission can significantly affect the interpretation of plant–plant interactions, as comparing species with fundamentally different competitive abilities can lead to overestimation or underestimation of competitive dynamics^88^. To estimate the competitive ability of the four competitor species, we conducted an ancillary experiment with 240 additional pots (Ancillary Experiment 6 in Supplementary Note 1). There was no significant difference in competitive ability among the four competitor species and *Erigeron* (F_(5,98)_ = 0.718; P = 0.582; Supplementary Fig. 15) or between drought treatments (F_(5,98)_ = 0.081; *P* = 0.777; Supplementary Fig. 15). These results indicate that under our experimental conditions, the competitive abilities of the competitor species did not differ substantially from those of *Erigeron*.

### Population co-ancestry of the sampled populations

Genomic analyses were conducted using the subset of individuals from Lucas et al. ^17^ that were included in the present experiment. SNP generation, sequencing, variant calling, and filtering followed the methods described in that study and are summarised in Supplementary Note 3. Four healthy and vigorous leaves were sampled from one individual per population (list of their identifiers can be found in Supplementary Data 2) for DNA extraction, and SNP data were generated using double-digest restriction site-associated DNA sequencing (ddRADseq). Paired-end sequencing was performed on a NovaSeq 6000 (Illumina, San Diego, USA) and identified single nucleotide polymorphisms (SNPs) using dDocent, version 2.7.8^89^. Subsequent SNP filtering steps retained a single SNP per contig, required a minor allele frequency of 0.05, and retained only SNPs that could be regarded as neutral (Supplementary Note 3).

In total, 4601 biallelic and neutral SNPs were identified, which were used to investigate co-ancestry among populations by creating an identity by descent (IBD) matrix, using the gl.grm function from the dartR package, version 2.9.7^90^. This IBD matrix quantified the populations’ pairwise co-ancestries and was incorporated into our PMMs (see below). In addition, the genetic structure of the populations was assessed using a Bayesian clustering approach. Admixture coefficients of the populations were estimated using the snmf function from the LEA package, version 4.4^91^. Based on the elbow plot of the clustering results, we found that the genetic structure of our populations was best described by four clusters (*K* = 4; Supplementary Fig. 6). Each population was assigned to a distinct genetic cluster according to its most dominant cluster membership (Supplementary Data 1; Supplementary Fig. 7a).

In addition, genetic relationships among populations were visualised using principal component analysis (PCA) (Supplementary Fig. 7b). PCA was conducted using the glPca function from the adegenet package, version 2.1.11^92^. To further examine genetic relationships and potential network patterns among populations, we constructed a neighbour-joining tree based on Nei’s genetic distances (Supplementary Fig. 7c). Pairwise genetic distances were calculated using the stamppNeisD function from the StAMPP package, version 1.6.3^93^, based on population-level allele frequencies. The neighbour-joining tree was then created using the nj function from the ape package, version 5.8-1^94^.

### Statistical analyses

#### General treatment validation

The effects of competition and drought on focal *Erigeron* biomass, stomatal conductance, and δ¹³C isotope ratios were analysed using linear mixed-effects models. These models tested the interaction between competition and drought treatments, including population nested within region as a random effect. Soil water content and pot weight, measured repeatedly over time, were analysed using linear mixed-effects models with time, drought treatment, and their interaction as fixed effects, and pot identity included as a random effect to account for repeated measurements, with Bonferroni correction applied.

To examine survival under different treatment combinations, we performed mixed-effects Cox proportional hazards models, implemented in the R package coxme, version 2.2-16^95^. The interactive effects of competition and drought treatments were tested, including population nested within region as a random effect. Assumptions of proportional hazards were evaluated with Kaplan-Meier plots, and multicollinearity was assessed using the variance inflation factor for each model^96^.

#### Non-native Erigeron populations have evolved increased competitive ability compared with native populations, but drought stress may alter these differences between ranges

Our model tested how competitive ability was affected by the interaction between range and drought treatment. To account for drivers of among-population variation within both ranges, we also included the additive effects of two covariates: the log_e_-transformed climatic water deficit (a proxy for local aridity conditions in the field) and the log_e_-transformed community field biomass (a proxy for the competitive regime in the field). The population nested within the region was set as a random factor. The full model structure is provided in Supplementary Table 1.

#### Competitive ability is influenced by population co-ancestry, with genetically closely related populations expected to show similar competitive responses

To account for co-ancestry in our above-mentioned model, we used pedigree mixed-effects models (PMMs), which weighted the random effect of population according to pairwise co-ancestry, treating populations with higher co-ancestry as less independent samples^35,53^. These PMMs were implemented using the R package pedigreemm, version 0.3-3^97^, employing a modified version of the pedigreemm function following Lachmuth et al.^53^. To assess the impact of co-ancestry on competitive ability, we compared the variance explained by the random effect of population between PMMs and the corresponding standard mixed-effects models (MMs). MMs were performed using the lme4 package, version 1.1-35.5^98^. Any additional variance explained by the population effect in PMMs relative to the respective MMs reflects the contribution of co-ancestry to variation in competitive ability^53^. In other words, such an increment describes how much among-population variation in competitive ability is shaped by co-ancestry, for example through random dispersal patterns or genetic drift^16^. To explore potential differences in the role of co-ancestry between ranges, we repeated the same model separately for native and non-native populations, excluding the fixed effect of range.

We also examined whether competitive ability varied among genetic clusters and how this variation is influenced by geographic range (native vs. non-native). Our goal was to determine whether enhanced competitive ability evolved across all clusters or only in specific ones, and whether competition-related traits diverged differently between native and non-native regions. Genetic clustering was analysed as a factor, using the highest cluster affiliation of each population. The model structure was similar to the previous model, except that we included admixture clustering as an additional explanatory variable (in two-way interaction with range and two-way interaction with drought treatment) in MMs (Supplementary Table 2). We used MM for this analysis because cluster identity represents a discrete summary of genetic structure derived from the same SNP data as the co-ancestry matrix; including both in the same model would introduce collinearity and confound the interpretation of their effects.

#### Evolutionary changes in competitive ability are stronger in interspecific interactions than in intraspecific interactions and may vary between competitive suppression and tolerance

To determine whether intra- or interspecific competition contributed most to the observed patterns in competitive ability, we analysed the estimates of competitive tolerance (αii and αij in Equation 1) and competitive suppression of *Erigeron* (αii and αij for the corresponding approach estimating suppressive effects of the focal *Erigeron*; Ancillary Experiment 4 in Supplementary Note 1) in two separate pedigree mixed-effects models. The model structure was similar to that used for competitive ability, except that the explanatory variables included the two-way interactions of range with competition type and drought treatment with competition type (Supplementary Table 3).

#### Model diagnostics

For all models, we applied stepwise backward model selection based on χ^2^-tests to assess the significance of fixed effects. Normality of residuals and variance homogeneity were evaluated by visually inspecting quantile–quantile plots and residual histograms for models with transformed vs. untransformed variables. Competitive ability was log_e_-transformed prior to the analyses. Climatic water deficit and community field biomass were log_e_-transformed and scaled prior to the analyses.

#### Spatial autocorrelation assessment

To assess the extent of spatial autocorrelation in competitive ability, we applied Moran’s *I* correlograms with the correlog-function of the R package ncf, version 1.3-2^99^. We ran 1000 permutations to estimate the significance of Moran’s *I* at discrete distance classes. The uniformly distributed distance classes were set at 100-km intervals. These assessments allow testing whether setting population nested within region as a random effect appropriately accounts for spatial autocorrelation (see Nagy et al. ^34^ for details). The Moran’s *I* statistics indicated that, without nesting population within region as a random effect, competitive ability showed evidence of spatial autocorrelation. The estimated distance at which objects were no more similar than expected by chance (i.e., x-intercept) was 43.52 km. More specifically, we found that the first distance intervals showed highly significant Moran’s *I* values, but this spatial autocorrelation decreased strongly after a few intervals. In contrast, when setting population nested within region as a random effect, the model showed no spatial autocorrelation. We conclude that our competitive ability data showed spatial autocorrelation due to regional structuring and that setting population nested within region as a random effect appropriately accounted for this pattern (see also Rosche et al. ^35^).

#### Ancillary experiments

Statistical analyses on the ancillary experiments are explained in the respective Supplementary Notes.

### Reporting summary

Further information on research design is available in the Nature Portfolio Reporting Summary linked to this article.

## Supplementary information

Supplementary Information

Description of Additional Supplementary Files

Supplementary Data 1

Supplementary Data 2

Reporting Summary

Transparent Peer Review file

## Source data

Source Data

## Data Availability

The experimental data generated in this study are available in the Figshare database at (https://doi.org/10.6084/m9.figshare.29881193). The demultiplexed raw sequence data are available in the European Nucleotide Archive (ENA) at EMBL-EBI under accession code PRJEB91610 and sample accession numbers ERS25137122 -ERS25137519, ERS25137526-ERS25137761. The list of identifiers of the individuals used in this experiment can be found in Supplementary Data 2. Source data are provided with this paper.

## Code availability

The R code is publicly available in the Figshare database at (https://doi.org/10.6084/m9.figshare.29881193).
