# Supplementary material for: Rapid and repeated evolution of increased competitive ability in a global invader
Source: Nat Commun. 2026 Sep 17;17:9930. doi: 10.1038/s41467-026-77819-z (PMC13586298; doi:10.1038/s41467-026-77819-z)
Supplement: Supplementary file 1 [file 41467_2026_77819_MOESM1_ESM.pdf]

Supplementary Information

Article title: Rapid and repeated evolution of increased competitive ability in a global invader

The following Supplementary Information is available for this article:

Supplementary Note 1. Ancillary experiments supporting the main experiment.

- **Ancillary Experiment 1.** Estimating the initial *Erigeron* biomass.
- **Ancillary Experiment 2.** Estimating the relationship between fecundity and biomass.
- **Ancillary Experiment 3.** Monitoring the effect of drought treatment.
- **Ancillary Experiment 4.** Estimating the competitive suppression of *Erigeron*.
- **Ancillary Experiment 5.** Estimating the effect of density on competitive ability.
- **Ancillary Experiment 6.** Estimating the competitive ability of competitors.

Supplementary Note 2. Quantifying climatic coverage of the sampling.

Supplementary Note 3. Description of population genomic analyses.

Supplementary Table 1. Details of the pedigree mixed-effects models on competitive ability.

Supplementary Table 2. Details of the linear mixed-effects models on competitive ability, using genetic clustering.

Supplementary Table 3. Details of the pedigree mixed-effects models on competitive tolerance and suppression.

Supplementary Table 4. Parameters used during the dDocent filtering.

Supplementary Fig. 1. The effect of drought treatment on pot environment and physiological traits.

Supplementary Fig. 2. Conceptual overview on biogeographical patterns in competitive suppression.

Supplementary Fig. 3. Biomass production and survival of the focal *Erigeron canadensis* individuals under drought condition.

Supplementary Fig. 4. Relationship between aboveground biomass and the number of capitula.

Supplementary Fig. 5: Relationship between competitive ability under mesic and dry conditions.

Supplementary Fig. 6. Elbow plot for the optimal number of genetic clusters (ΔK) based on ddRADseq analysis.

Supplementary Fig. 7. Genetic structure of the *Erigeron* populations.

Supplementary Fig. 8. Drought response of competitive ability differs among the genetic clusters of *Erigeron*.

Supplementary Fig. 9. Effect of genetic clustering coefficients on the competitive ability.

Supplementary Fig. 10. Drought response of competitive tolerance and suppression of *Erigeron* across inter- and intraspecific conditions

Supplementary Fig. 11. Relationship between competitive tolerance and suppression.

Supplementary Fig. 12. Preliminary screening of candidate competitor species.

Supplementary Fig. 13. Relationship between seedling biomass and seedling area.

Supplementary Fig. 14. Relationship between the measures of competitive interactions in the main experiment and those estimated using a density-dependent approach.

Supplementary Fig. 15. Competitive ability of the four competitor species and *Erigeron canadensis*.

Supplementary Fig. 16. Relationship between Similarity and Contigs for different (k1, k2) pairs.

SI References

Supplementary Note 1. Ancillary experiments supporting the main experiment

Ancillary Experiment 1: Estimating the initial *Erigeron* biomass

Competitive ability indices following Hart et al.[1] require an estimate of intrinsic growth rate (λ_i), which quantifies biomass gain in the absence of competitors. Growth rate is inherently a change over time, so it must account for the size of individuals at the beginning of the experimental period. Without initial biomass, the estimate of λ_i would conflate true growth potential with size advantages already present before the competition and drought treatments started. Directly measuring biomass at the start would require destructive harvesting, which is incompatible with following the same individuals to final harvest. Therefore, we used a non-invasive allometric approach: we photographed seedlings in the rosette stage under standardized conditions (same height, angle, camera settings) and calculated total leaf area as a proxy for biomass. This allowed us to estimate initial biomass from an established area–biomass relationship (see Mráz et al.[2]) and calculate λ_i as the log-transformed change in biomass from the start to the final harvest. By including initial biomass in our calculations, we ensured that competitive ability reflects true growth performance rather than pre-existing size differences, thus yielding biologically meaningful and comparable values across populations.

We grew an additional 100 pots (one individual each) from seeds of 50 randomly selected native and 50 non-native populations (Supplementary Data 1). Six weeks after sowing, coinciding with the start of the drought treatment, each of these 100 seedlings was photographed using our standardized photo station setup. At the same time, we photographed all individuals in the main experiment under identical conditions. Seedling area was measured from the photographs in ImageJ Version 1.54c (Laboratory for Optical and Computational Instrumentation, University of Wisconsin–Madison, USA).

Immediately after photographing, the 100 additional seedlings were harvested to measure their dry biomass. We used these biomass and seedling area data to fit a linear regression model, yielding the calibration equation (Supplementary Fig. 13; intercept = 0.0017, slope = 0.0041). This equation was then applied to the seedling area data of all individuals in the main experiment to estimate their initial biomass. These estimates were averaged per population and used to calculate intrinsic growth rates for competitive ability assessment (sensu Hart et al.[1]).

Ancillary Experiment 2: Estimating the relationship between fecundity and biomass

Many traits are used in research to estimate competitive success. Among these, biomass is a frequently used proxy, but it remains unclear whether it is the best trait to identify the long-term winner of competition[3]. Thus, it is important to consider not only the individual performance but also the reproductive success of a given genotype[4]. To address this, we estimated how fecundity of the included *Erigeron* populations related to their biomass production at harvest.

In total 200 additional pots were installed parallel to the main experiment, with 50 native and 50 non-native populations in two replications (Supplementary Data 1). Plants were grown together with the main experiment, and one individual from each population (100 plants) was harvested for biomass at the same time as the main experiment. The other 100 plants stayed in the glasshouse until full bloom stage (i.e. no more, new capitula formed, the majority of capitula have ripped seeds, but before seed loss). In this stage, the number capitula was counted to estimate fecundity.

The relationship between biomass at the harvest with the number of capitula was tested using pedigree mixed-effects model. We found a significant relationship between both variables (Supplementary Fig. 4.; df =1; $F = 80.126$; $P < 0.001$; $R^2 = 0.452$), supporting value of aboveground biomass as a fitness proxy.

Ancillary Experiment 3: Monitoring the effect of drought treatment

To monitor the efficacy of the applied drought treatment, an ancillary experiment was set on with 30 native and 30 non-native *Erigeron* populations (Supplementary Data 1), resulting in a total of 360 additional pots (2 drought treatments × 3 competition treatments × 60 populations). These pots were randomly distributed across the trays of the main experiment. Soil water content and pot weight were monitored for the 360 pots on a bi-weekly basis (every second Wednesday). The soil water content was measured using a Theta Probe Typ ML2x Soil Moisture Device (Delta T Devices, Cambridge, UK).

Moreover, at the harvest, we measured stomatal conductance and $\delta^{13}C$ isotope ratio for 120 of the 360 focal *Erigeron* individuals (10 from each range × treatment combination, randomly chosen). The largest leaf was considered for measurement of stomatal conductance using an AP4 Porometer (Delta T Devices, Cambridge UK). The porometer was calibrated before each measurement.

To determine the $\delta^{13}C$ isotope ratio, we collected three medium-sized leaves. They were dried in an oven at 60° C and grounded prior being analysed with an elemental analyser Flash 2000 with a TCD detector that was coupled to a Conflo IV and mass spectrometer Delta V Advantage (Thermo Fisher Scientific, Bremen, Germany) at the Center for Stable Isotope Research of Charles University, Prague, Czechia. The carbon isotope ratio was expressed as follows: $\delta^{13}C = (R_{sample} / R_{standard} - 1) * 1000$, where R is the relative abundance of the carbon isotopes ($R = {}^{13}C/{}^{12}C$) and reported using the Vienna Pee Dee Belemnite scale. A series of international standards (IAEA-CH3, IAEA-CH6 and IAEA-600) were used as repeated measurements to normalize the measured data. In addition, a birch leaves standard (Elemental Microanalysis Ltd., UK) was run after every 10th sample to quality control for the isotopic measurements. The analytical precision was within ±0.1 ‰.

Ancillary Experiment 4: Estimating the ability of *Erigeron* to suppress competitors

There has been a long-standing debate on how to predict which species will win or lose in competition[1,5,6]. Competitive ability consists of two components: the ability to suppress neighbours (competitive effect or suppression) and the ability to withstand their competitive impact (competitive response or tolerance[7]). Classical theory suggests that when two competitors are of similar size, suppression and tolerance contribute equally to overall competitive ability[8]. In contrast, in more natural, complex multispecies assemblages, selection is expected to favour tolerance because its benefits accrue entirely to the tolerant individual, whereas the benefits of suppression are shared among all nearby competitors[8,9]. Nevertheless, suppression may provide a decisive advantage in certain contexts, particularly during early establishment or when resources are highly limited[10,11]. We therefore quantified both components to capture their potentially independent contributions to the competitive success of *Erigeron* and also to investigate whether selection has a greater impact on tolerance or suppression in the course of *Erigeron*'s invasion.

We established an ancillary experiment in parallel with the main experiment. While the main experiment provided population-level estimates of competitive response, the ancillary setup supplied the corresponding estimate of competitive effect. We grew 240 additional pots (60 replicates per treatment combination) in which interspecific and intraspecific competitor communities (four individuals per pot) were planted without the focal *Erigeron* under mesic and dry conditions (see Supplementary Fig. 2). Interspecific communities comprised the same four species used in the main experiment. Intraspecific communities consisted of four *Erigeron* individuals drawn from 30 randomly selected native and 30 randomly selected non-native populations (Supplementary Data 1). Sowing dates, treatment initiation, and harvest coincided with the main experiment. At harvest, shoots were collected and dried to determine community biomass. Mean community biomasses were: interspecific × dry = 0.747 g; interspecific × mesic = 1.728 g; intraspecific × dry = 0.722 g; intraspecific × mesic = 1.371 g. These *Erigeron*-free community biomasses served as the reference against which suppression by focal *Erigeron* was assessed.

To quantify the competitive suppression (competitive effect) exerted by each focal *Erigeron* population on its interspecific and intraspecific competitor communities, we calculated competition coefficients (α) following the approach of Hart et al.[1]. For each treatment combination (interspecific × mesic, interspecific × dry, intraspecific × mesic, intraspecific × dry), we compared community biomass in pots without a focal *Erigeron* (measured in this ancillary experiment) to community biomass in pots from the main experiment where the same community grew with a focal *Erigeron* individual in the centre.

Specifically, for each *Erigeron* population, competitive suppression was calculated as:

$$\alpha = \ln(B_{\text{without } \textit{Erigeron}}) - \ln(B_{\text{with } \textit{Erigeron}})$$

where $B_{\text{without } Erigeron}$ represents the mean biomass of the *Erigeron*-free community (data from the ancillary experiment), and $B_{\text{with } Erigeron}$ represents the biomass of the same community when grown with the focal *Erigeron* population (data from the main experiment). This $\log_e$-transformed difference expresses the proportional reduction in community biomass attributable to the focal *Erigeron*, scaled per competitor. We computed these suppression indices separately for each treatment combination, resulting in four suppression values per population (interspecific × mesic, interspecific × dry, intraspecific × mesic, intraspecific × dry). Conceptually, these indices are directly parallel to the competitive tolerance values, but from the competitor community's perspective rather than the focal *Erigeron*'s.

The resulting suppression indices were analysed using pedigree mixed models (PMMs) as described in the main text, with model structures detailed in Supplementary Table 3. Results are presented in Fig. 4 and Supplementary Fig. 10 and 11.

Ancillary Experiment 5: Estimating the effect of density on competitive ability

Current research on competition indicates that plant–plant interactions are often density-dependent[1,12,13]. Therefore, simple comparisons of performance with versus without competitors, as applied in our study, may not fully capture competitive interactions, especially when non-linear relationships exist between competitor density and the effects of competition[1]. To address such complexity in species interactions, competition coefficients (α) are generally recommended to be estimated using more elaborate experimental designs rather than simple with-versus-without competitor comparisons. For example, response-surface designs allow accurate parameterization of mathematical models of competition because they mimic the dynamics of species populations across space and time[14]. However, while our main focus was to assess a broad set of populations rather than to precisely quantify density effects, we conducted an additional experiment to examine density-dependence in a representative subset of *Erigeron* populations.

To examine how density affects competitive outcomes, we performed a density-dependence experiment with a subset of our *Erigeron* populations (three from the native and three from the non-native range, Supplementary Data 1). Populations were chosen to represent distinct geo-climatic regions, but parallel between ranges. Seeds were sown to obtain a density gradient with seven levels: 1 (without interactions), 2, 3, 5, 7, 9, and 12 seedlings per pot. Each density level (except when *Erigeron* grew alone) was tested in intra- and interspecific combinations. To test how density-dependence varied under different water supply conditions, the same mesic vs dry treatments were applied from (Supplementary Data 1.). In total, 540 pots were installed: 6 populations × 15 density levels (seven intra- and inter specific + one alone) × 2 experimental droughts (mesic and dry) × 3 replications. The timing for sowing (*Erigeron* and the competitors), initiating the treatments and harvest were the same as for the main experiment.

For each population × drought treatment × competition type, dried shoot biomass of the focal *Erigeron* individuals was recorded and analysed with linear models, using competitor density as the explanatory variable. From these regressions, the intercept was taken as an estimate of the intrinsic growth rate (λ) and the slope as an estimate of the competition coefficient (α). Competitive ability indices were then calculated using the same formula as in the main experiment (see Equation 1). Finally, we tested the relationship between competitive abilities derived from the density-dependent approach (Ancillary Experiment 5) and those obtained from the simplified pairwise approach (Main Experiment) for the six overlapping populations.

Ancillary Experiment 6: Estimating the competitive ability of competitors

Understanding the outcome of competition requires not only assessing the competitive ability of the focal species, but also of the species it interacts with. If competitor species differ strongly in their own competitive strength, comparisons of focal species performance may be biased, as the outcome could reflect the inherent strength or weakness of the competitor rather than differences in the focal species itself[5,7]. Therefore, evaluating the competitive ability of both focal and competitor species provides a more balanced view of interaction outcomes and allows us to determine whether observed patterns are driven by the focal species' evolutionary changes or by differences among its competitors.

To gain a more comprehensive understanding of the competitive interactions between *Erigeron* and its competitor community, we assessed the competitive ability of each species within the community. For this, we applied the same three competition regimes (alone, intra-, and interspecific) to the four competitor species (*Bromus tectorum* L., *Lactuca serriola* L., *Plantago lanceolata* L., *Trifolium repens* L.). Each competitor was either planted alone, surrounded by four conspecifics, or surrounded by four heterospecifics (i.e., the other three competitors and one *Erigeron* individual). To explore how the competitive ability of the competitors varied under different water supply conditions, the same mesic and dry treatments were applied.

In total, 240 pots were set up: 4 species × 3 competition regimes × 2 drought treatments × 10 replications. Additionally, 40 pots (10 per competitor species) were harvested at the onset of the experimental drought to assess initial biomass which we did using the same procedure as described for the focal *Erigerons* (Ancillary Experiment 1, Supplementary Note 1). In this experiment one *Erigeron* population was used (CEU-PLA). Sowing dates (for both *Erigeron* and the competitors), as well as the initiation of treatments and harvests, coincided with the main experiment. At harvest, shoots were collected and their dry biomass measured.

To quantify the competitive ability of the competitor species, we applied the same calculation framework as for *Erigeron*, adapted from Hart et al.[1]. For each competitor species × drought treatment, we first estimated intrinsic growth rate (λ_i) as the $\log_e$-transformed difference between its biomass at harvest and its estimated initial biomass. Competitive tolerance values (α_{ii} and α_{ij}) were calculated as the differences between the $\log_e$-transformed biomasses in the alone treatment and the intraspecific or interspecific treatments, respectively, divided by the number of competitors. Competitive ability indices were then calculated using the same formula as in the main experiment (see Equation 1). These indices allowed us to directly compare the competitive ability of the four competitor species with that of *Erigeron*, under both mesic and dry conditions. To statistically test for differences, we fitted a linear mixed-effects model with the $\log_e$-transformed competitive ability index as the response variable, and species identity and drought treatment as fixed effects.

Supplementary Note 2. Quantifying climatic coverage of the sampling

We quantified how well our sampled populations covered the climatic niche of *Erigeron* across the Northern hemisphere, following suggestions made by Thoma et al.[15]. Climatic coverage was evaluated with dynamic range boxes (DRB[16]), which we used to estimate the overlap between sampled climatic conditions and the species' realised niche in native and nonnative ranges. Occurrence records and sampling sites were linked to bioclimatic variables to define overall and sampled climatic niches, respectively. To reduce bias from intercorrelation, we excluded bioclimatic variables with an absolute pairwise correlation > 0.8. This resulted in the selection of seven variables: BIO1: Annual mean temperature, BIO2: Mean diurnal range, BIO3: Isothermality, BIO12: Annual precipitation, BIO14: Precipitation of the driest month, BIO15: Precipitation seasonality and BIO19: Precipitation of the coldest month. DRB values range from 0 to 100%, with higher values reflecting greater overlap between the sampled climatic space and the species' realised niche.

Supplementary Note 3. Description of the population genomic analyses

Plant Material and DNA Extraction

Four fresh *Erigeron* leaves per mother plant were collected during our glasshouse experiment, freeze-dried, and stored in silica gel until DNA extraction. Genomic DNA was extracted from the dried, macerated leaves using the peqGOLD Plant DNA Mini Kit (Avantor, Radnor, USA). DNA was quantified using a Qubit 2.0 fluorometer (Thermo Fisher Scientific, Waltham, USA) and DNA concentration was increased by evaporation when necessary.

Genomic data

We used a modified version of the ddRADseq protocol of Peterson et al.[17] to construct reduced-representation libraries of genomic DNA for the genotyping of single nucleotide polymorphisms (SNPs), as described in Durka et al.[18]. Approximately 200 ng of DNA from each sample was digested with EcoRI-HF and MspI using Cutsmart buffer (NEB) in a total volume of 13 µl, with an incubation period of 8 h at 37°C. Custom-barcoded adapters were then ligated to restriction fragments. Groups of 96 samples, each with unique barcodes, were pooled in equal volumes, purified with Wizard Gel and PCR Clean-up Kit (Promega, Waldorf, Germany), and size-selected using the Blue Pippin electrophoresis platform to select fragments of 350-450 bp in length. Unique external indices were added to each pool through PCR amplification. The PCR products were purified with a clean-up kit and AMPure XP beads. Fragment sizes were verified on a 2100 Bioanalyzer (Agilent, Santa Clara, USA). Libraries were then pooled in equimolar quantities and sequenced on an NovaSeq™ 6000 (Ilumina, San Diego USA) Sequencing System using 10% PhiX resulting in approx. 5 Mio sequences per sample.

SNP genotyping

Raw reads were demultiplexed using the process_radtags module from the Stacks pipeline, version 2.68[19]. Quality filtering, de novo assembly, reference construction, read alignment, and SNP calling were carried out with dDocent, version 2.7.8[20]. Default parameters were applied throughout (Supplementary Table 4), except for clustering similarity, which yielded better results at a threshold of 0.9 (Supplementary Fig. 16). Subsequently, we filtered loci and individuals following O'Leary et al.[21], using the filtering script available at https://doi.org/10.5281/zenodo.10977129. First, we used vcfallelicprimitives from the library vcflib, version 1.0.0[22] and vcftools, version 0.1.16[23] to remove indels. We kept only biallelic SNPs with a minimum allele count (mac) of 3, a minimum genotype read depth (minDP) of 3, a minimum mean sequence quality (minQ) of 30 and maximum missingness across individuals (max_missing) of 50 %. To exclude paralogous SNPs, we filtered putatively fixed heterozygous SNPs, by calculating the p-value for heterozygosity excess with vcftools and filtering SNPs with P-HET_EXCESS <1e-15. We skipped individuals with >75 % missing values (imiss). Subsequently, using vcffilter from vcflib, version 1.0.0[22], we filtered SNPs according to allele balance

(AB > 0.2 & AB < 0.8 | AB < 0.01 | AB > 0.99), strandedness (SAF / SAR > 100 & SRF / SRR > 100 | SAR / SAF > 100 & SRR / SRF > 100), mapping quality ratio of the two alleles (MQM / MQMR > 0.9 & MQM / MQMR < 1.05), and properly pairing alleles (PAIRED > 0.05 & PAIREDR > 0.05 & PAIREDR / PAIRED < 1.75 & PAIREDR / PAIRED > 0.25 | PAIRED < 0.05 & PAIREDR < 0.05). We then filtered SNPs to maximum missingness of 34 % (max_missing 0.66), the minimum minor allele frequency of (maf) 0.05, minimum mean read depth (min-meanDP) of 10, and a maximum mean depth (max-meanDP) of 1000. Thus, from a total of 801,773 SNPs in 41,149 contigs, 11501 SNPs in 5080 contigs were obtained of which we retained only one single SNP per contig.

The genotypic data were analysed using the dartR package in R[24]. Initially, the VCF file was converted into a genlight object, suitable for subsequent analyses. In order to obtain a putatively neutral SNP dataset, we excluded all loci identified by any of the three following approaches. First, we used PCAdapt, version 4.4.1[25] (Luu et al. 2016) as a genome scan method to detect potential loci under selection by identifying SNPs excessively correlated with population structure inferred from principal component analysis. We used the first two principal components that sufficiently represent the four major ancestral clusters identified by admixture (see main text). Second, we applied latent factor mixed models (LFMM) based on an exact least-squares approach to identify loci showing significant correlations to environmental gradients while accounting for population genetic structure using the LEA package, version 4.4[26]. Environmental variance was described by all 19 bioclim variables from the Worldclim database, version 2.1 http://www.worldclim.org; Hijmans et al.[27]) plus the climatic water deficit obtained from TerraClimate[28]. To account for covariance in environmental gradients, the environmental variables were summarized by projection on their principal components. We then ran the 'lfmm2' function for each of the first four principal components with the number of latent factors equal to the number of the four ancestral clusters identified by our admixture analyses. The p-values for the association between loci and environmental variables were obtained using the 'lfmm2.test' function for the full set of environmental variables and subsequently false discovery ratecorrected[29] retaining loci with significant q-values ($\alpha < 0.05$). Third, we used a redundancy analysis (RDA) to model SNP allelic frequencies by environmental predictors while accounting for ancestral groups. This RDA allowed us to identify outlier loci in environmental space using the 'rdadapt' function described by Capblancq et al.[30]. Similar to the LFMM approach, we included demographic history as covariate, here represented by the admixture coefficients for $K = 4$. The 'rdadapt' function identifies outliers based on Mahalanobis distances, incorporates an inflation factor[31] and calculates q-values. We retained loci with $q < 0.05$. With the abovementioned filtering options 479 adaptive loci were identified to be potentially under selection. These loci were discarded, resulting in 4,601 neutral loci, used for the analyses.

Supplementary Table 1. Details of the pedigree mixed-effects models investigating how competitive ability was related to range (native v. non-native), drought treatment (mesic vs. dry), climatic water deficit and community biomass in the field, across 100 native and 165 non-native *Erigeron* populations.

Fixed effects	Competitive ability		
	$\chi^2_{(1)}$	*Estimate*	*P - value*
(Intercept)		3.07	
$Range_{Non-native}$: $Drought\ treatment_{Mesic}$	2.69	0.11	0.1
$Range_{Non-native}$	13.89	0.22	< 0.001
$Drought\ treatment_{Mesic}$	16.66	0.13	< 0.001
Climatic water deficit	1.5	0.03	0.22
Community biomass	1.33	-0.02	0.25
Random effect variances			
Region:Population		0.07 (265)	
Region		<0.01 (21)	
Residuals		0.13 (525)	

Notes: The first column of the table shows the model structure of the maximal models. Competitive ability was analysed using a pedigree mixed-effects model (PMM). Significance of fixed effects was assessed using likelihood ratio (χ^2) tests by comparing nested models. Pairwise comparisons, where applicable, were performed using Tukey-adjusted post hoc tests. χ^2-values, parameter estimates, and significance levels of fixed effect terms that remained in the minimal adequate models are given. Range and treatment are denoted with the second factor level. Random effect variances of the region and population nested within region are presented with their number of groups in parentheses (i.e., 265 populations, 21 geographical regions). Random effect variances of residuals are presented with the number of observations.

Supplementary Table 2. Details of the linear mixed-effects models investigating how competitive ability was related to range (native v. non-native), drought treatment (mesic vs. dry), genetic clustering, climatic water deficit and community biomass in the field, across 100 native and 165 non-native *Erigeron* populations.

Fixed effects	Competitive ability		
	$\chi^2_{(1)}$	*Estimate*	*P - value*
(Intercept)		0.28	0.28
$Range_{Non\text{-}native}$: Admixture $cluster_2$	2.69	-0.06	0.26
$Range_{Non\text{-}native}$: Admixture $cluster_3$	2.69	-0.07	0.26
$Range_{Non\text{-}native}$: Admixture $cluster_4$	2.69	-0.25	0.26
Drought $treatment_{Mesic}$: Admixture $cluster_2$	4.96	-0.17	0.17
Drought $treatment_{Mesic}$: Admixture $cluster_3$	4.96	-0.14	0.17
Drought $treatment_{Mesic}$: Admixture $cluster_4$	4.96	-0.19	0.17
$Range_{Non\text{-}native}$	18.05	0.26	< 0.001
Drought $treatment_{Mesic}$	17.91	0.13	< 0.001
Admixture $cluster_2$	18.1	0.07	< 0.001
Admixture $cluster_3$	18.1	0.23	< 0.001
Admixture $cluster_4$	18.1	-0.07	< 0.001
Climatic water deficit	1.82	0.03	0.18
Community biomass	0.52	-0.01	0.47
Random effect variances			
Region:Population	0.02 (265)		
Region	<0.01 (21)		
Residuals	0.12 (525)		

Notes: The first column of the table shows the model structure of the maximal models. Competitive ability was analysed using a linear mixed-effects model (MM). Significance of fixed effects was assessed using likelihood ratio (χ^2) tests by comparing nested models. Pairwise comparisons, where applicable, were performed using Tukey-adjusted post hoc tests. χ^2 -values, parameter estimates, and significance levels of fixed effect terms that remained in the minimal adequate models are given. Range and treatment are denoted with the second factor level, admixture cluster with the second, third and fourth levels. Random effect variances of the region and population nested within region are presented with their number of groups in parentheses (i.e., 265 populations, 21 geographical regions). Random effect variances of residuals are presented with the number of observations.

Supplementary Table 3. Details of the pedigree mixed-effects models investigating how competitive tolerance and competitive suppression of *Erigeron* was related to range (native v. non-native), drought treatment (mesic vs. dry), competition type (inter- vs. intraspecific), climatic water deficit and community biomass in the field, across 100 native and 165 non-native *Erigeron* populations.

Fixed effects	Competitive tolerance of *Conyza*			Competitive suppression of *Conyza*		
	$\chi^2_{(1)}$	*Estimate*	*P - value*	$\chi^2_{(1)}$	*Estimate*	*P - value*
(Intercept)		0.29			0.25	
$Range_{Non\text{-}native}$: $Competition\ type_{Intra}$	9.21	0.05	0.002	13.80	0.15	< 0.001
$Drought\ treatment_{Mesic}$: $Competition\ type_{Intra}$	6.28	0.04	0.01	19.11	0.17	< 0.001
$Range_{Non\text{-}native}$	15.63	-0.08	< 0.001	0.23	0.09	0.63
$Drought\ treatment_{Mesic}$	0.22	-0.02	0.64	3.98	-0.13	0.05
$Competition\ type_{Intra}$	65.78	0.01	< 0.001	3.36	-0.03	0.07
Climatic water deficit	1.53	-0.01	0.22	0.09	-0.003	0.76
Community biomass	3.58	0.01	0.06	0.06	0.003	0.8
Random effect variances						
Region:Population		<0.01 (265)			0.01 (265)	
Region		<0.01 (21)			<0.01 (21)	
Residuals		0.02 (1050)			0.09 (1031)	

Notes: The first column of the table shows the model structure of the maximal models. Competitive tolerance and competitive suppression were analysed using pedigree mixed-effects models (PMMs). Significance of fixed effects was assessed using likelihood ratio (χ^2) tests by comparing nested models. Pairwise comparisons, where applicable, were performed using Tukey-adjusted post hoc tests. χ^2 - values, parameter estimates, and significance levels of fixed effect terms that remained in the minimal adequate models are given. Range and treatment are denoted with the second factor level. Random effect variances of the region and population nested within region are presented with their number of groups in parentheses (i.e., 265 populations, 21 geographical regions). Random effect variances of residuals are presented with the number of observations.

Supplementary Table 4. Parameters used during the dDocent, version 2.7.8[20] filtering process for all individuals in the study and their followed description. This includes computational resources allocated (processors and memory), sequence preprocessing steps (trimming, assembly type, clustering similarity), read inclusion criteria (coverage levels, individual inclusion thresholds), read mapping parameters (match values, mismatch penalties, gap penalties), and SNP calling settings.

**Parameter**	
Number of Processors	20
Maximum Memory	0
Trimming	Yes
Assembly?	Yes
Type_of_Assembly	PE
Clustering_Similarity%	0.9
Minimum within individual coverage level to include a read for assembly (K1)	5
Minimum number of individuals a read must be present in to include for assembly (K2)	6
Mapping_Reads?	Yes
Mapping_Match_Value	1
Mapping_MisMatch_Value	3
Mapping_GapOpen_Penalty	5
Calling_SNPs?	Yes

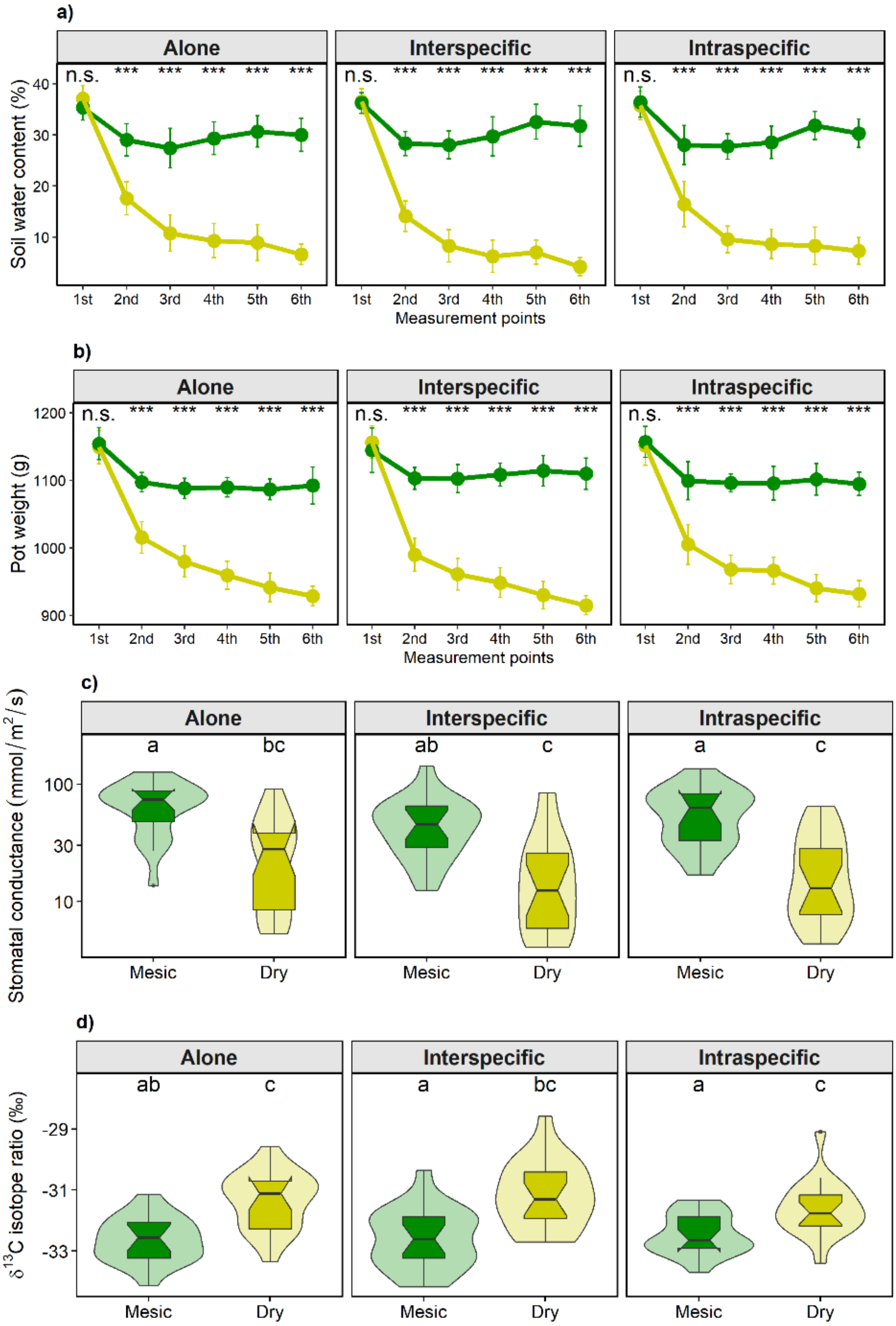

Supplementary Fig. 1: Drought treatment influenced the pot environment and physiological traits of *Erigeron canadensis*. a, Soil water content and b, pot weight were measured biweekly before watering. Significant differences between mesic-treated (green) and dry-treated individuals (yellow) are indicated by asterisks calculated in pairwise comparisons of a repeated measurements analysis, after Bonferroni correction (*, $P < 0.05$; **, $P < 0.01$; ***, $P \leq 0.001$; n.s., not significant). c, Stomatal conductance was measured at the time of the harvest. d, $\delta^{13}C$ isotope ratios were determined from leaves collected at harvest in an ancillary experiment (for details see Ancillary Experiment 3 in Supplementary Note 1). Lowercase letters indicate groupings based on Tukey's post hoc test. Stomatal conductance data are log-scaled, reflecting the $\log_e$-transformation used in statistical analyses. All measurements were performed on independent biological replicates (single pots containing one focal individual), with n = 20 per competition × drought treatment combination. Source data are provided as a Source Data file.

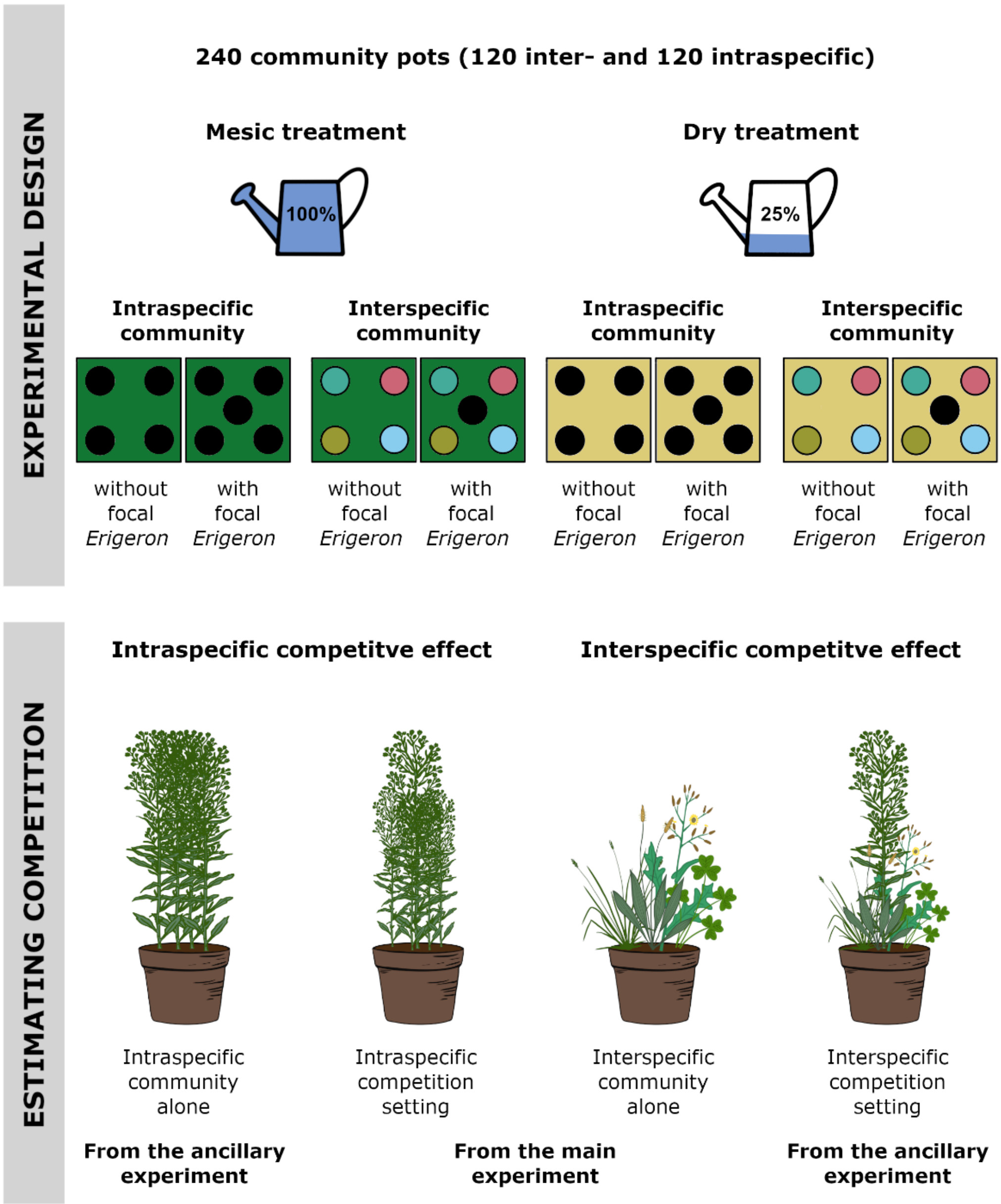

Supplementary Fig. 2: Conceptual overview of the ancillary experiment on competitive suppression of *Erigeron* under mesic and dry conditions. The 265 *Erigeron* populations used to calculate competitive suppression were the same as those used for competitive tolerance and competitive ability (see Fig. 1 in the main text). While our main experiment provided competitive tolerance values for each population, we here obtained corresponding measures of competitive suppression of these populations on the interspecific and intraspecific competitor communities. To quantify this competitive suppression of *Erigeron*, we conducted Ancillary Experiment 4, which involved 240 community pots (2 drought treatments × 2 community types × 60 replications). In this experiment, we estimated the mean biomass of communities grown without the focal *Erigeron* individuals. Competitive suppression was calculated as the $\log_e$-transformed difference between the mean competitor community biomass without the focal *Erigeron* (from Ancillary Experiment 4) and the competitor community biomass with the focal *Erigeron* (from the main experiment). This estimate corresponds to the competition coefficients (αii and αij, respectively) based on Hart et al.[1], mirroring the method used to calculate the competitive tolerance of the focal *Erigeron* plants but this time from the competitor communities' perspective.

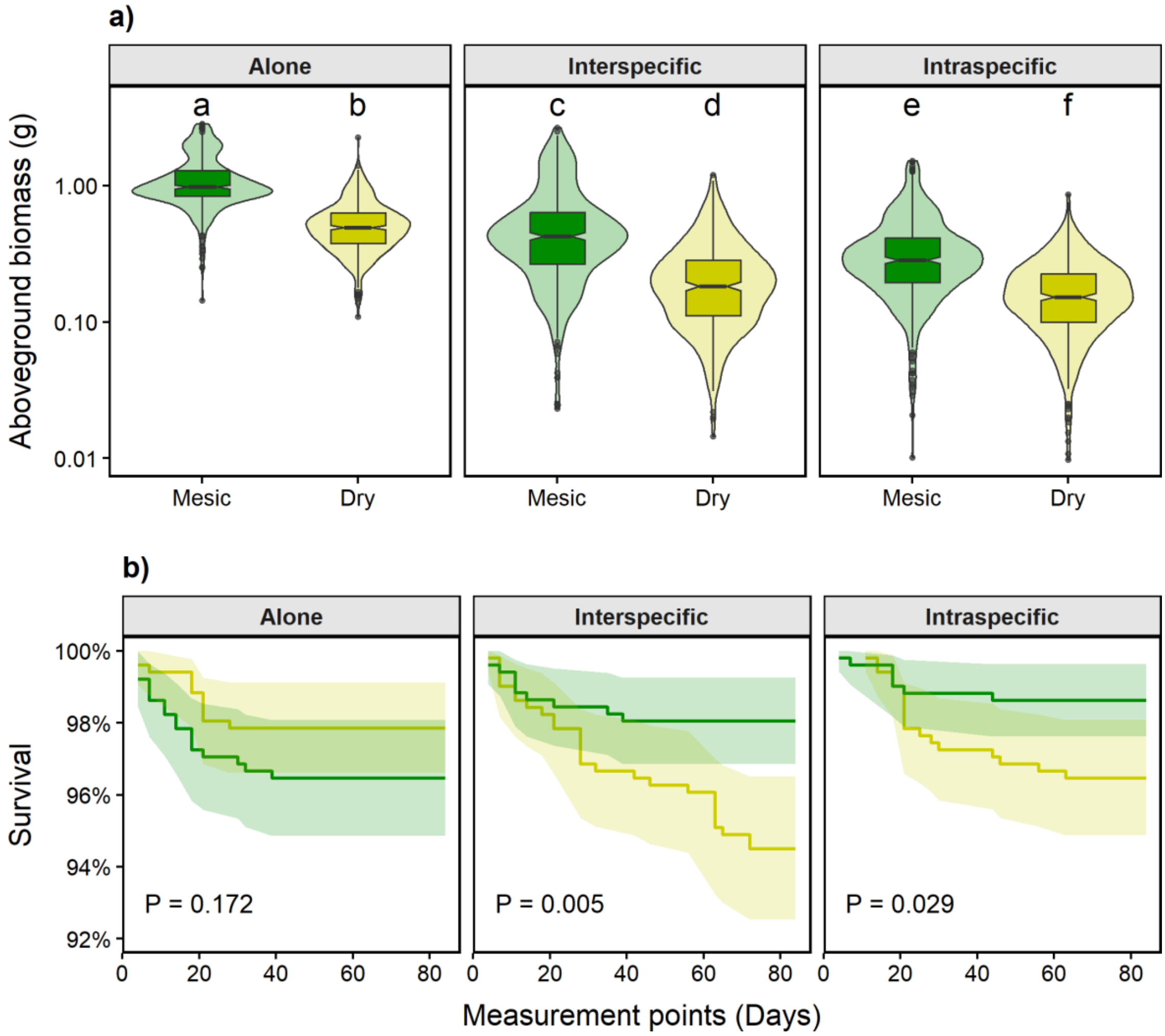

Supplementary Fig. 3: Biomass production and survival of the focal *Erigeron canadensis* individuals are affected by the applied competition and drought treatments. a, Biomass was measured at the end of the experiment (n = 2,778). b, Survival was recorded every two days during the 12-week drought treatment period (n = 3,408). Treatments included competition (grown alone, interspecific, or intraspecific) and drought (mesic = green; dry = yellow). Lowercase letters indicate groupings based on Tukey's post hoc test. Biomass data are log-scaled, reflecting the $\log_e$-transformation used in statistical analyses. Fig. 1 provides an overview of the experimental design. Source data are provided as a Source Data file.

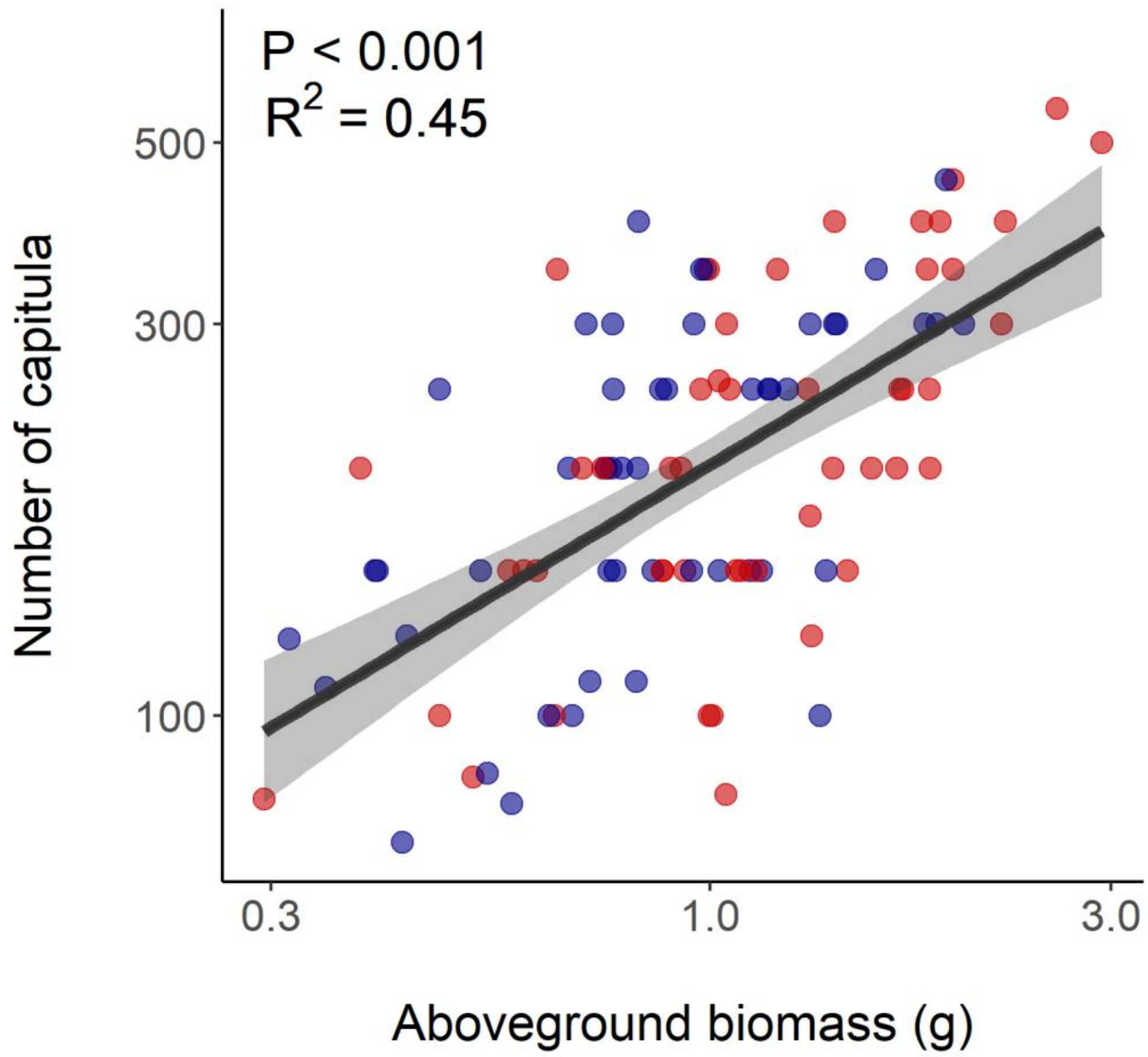

Supplementary Fig. 4: Positive relationship between aboveground biomass and the number of capitula in *Erigeron canadensis*. In an ancillary experiment with 200 pots (Ancillary Experiment 2 in Supplementary Note 1), the relationship between biomass at harvest and the number of capitula at full bloom was tested using n = 100 independent biological replicates originating from 50 native (blue) and 50 non-native (red) *Erigeron* populations. Aboveground biomass was measured at the time of harvest of the main competition experiment, whereas capitula number was quantified on separate individuals from the same populations that were allowed to grow until full reproductive maturity. The P- and R^2-values represent the results of a linear regression. The shaded band represents the 95% confidence interval of the linear regression. Source data are provided as a Source Data file.

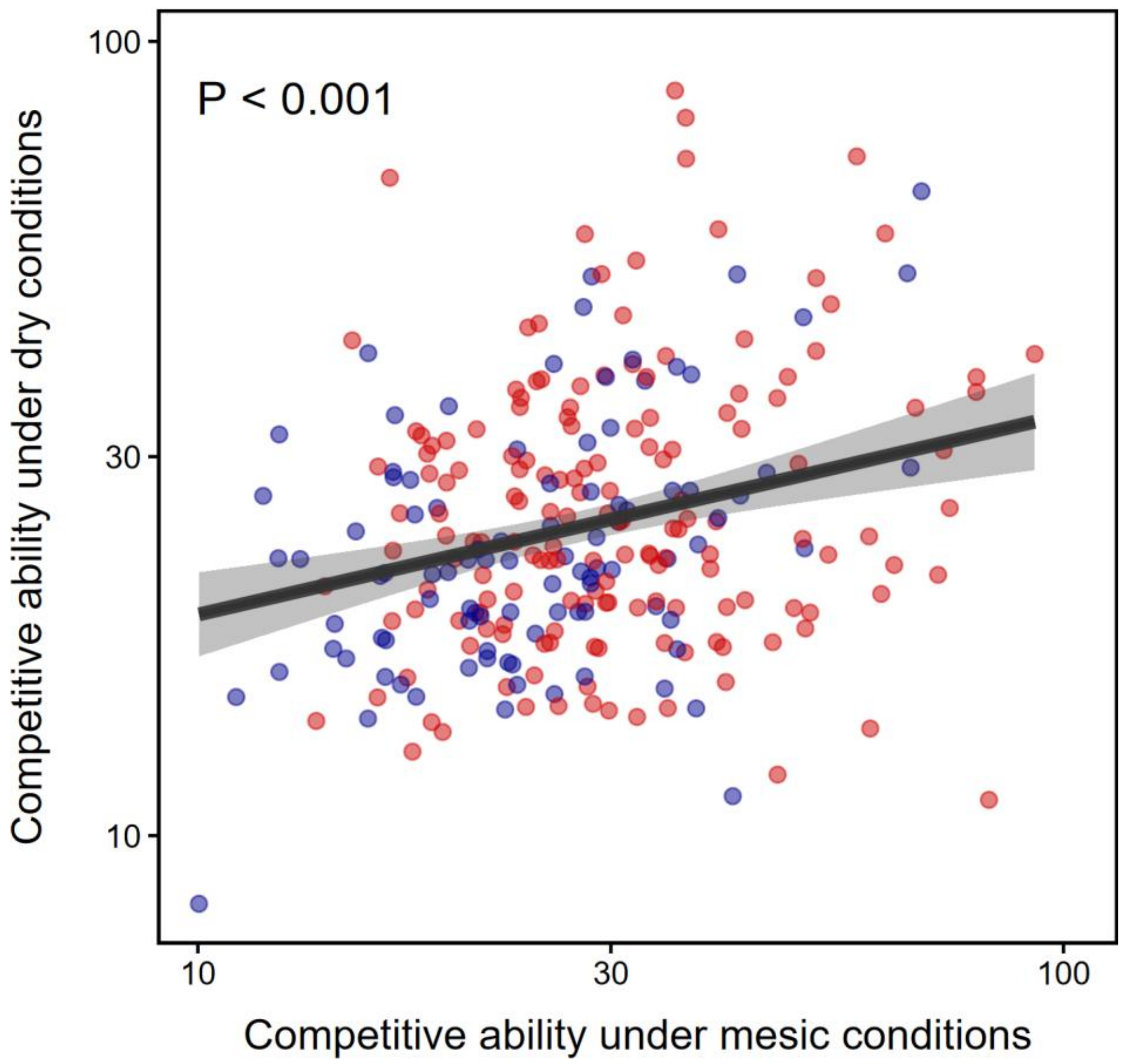

Supplementary Fig. 5: Relationship between competitive ability under mesic and dry conditions across 100 native (blue) and 165 non-native (red) *Erigeron* populations. This analysis aimed to determine whether drought alters the competitive hierarchy among populations. We found that competitive abilities of *Erigeron* under dry and mesic conditions were significantly correlated to one another (n = 265; $F_{(1,258)}$ = 19.97; $P < 0.001$; $R^2 = 0.072$; slope = 0.286). In other words, populations that are more competitive under mesic conditions also tend to be more competitive under drought stress. The shaded band represents 95% confidence intervals of the linear regression. Source data are provided as a Source Data file.

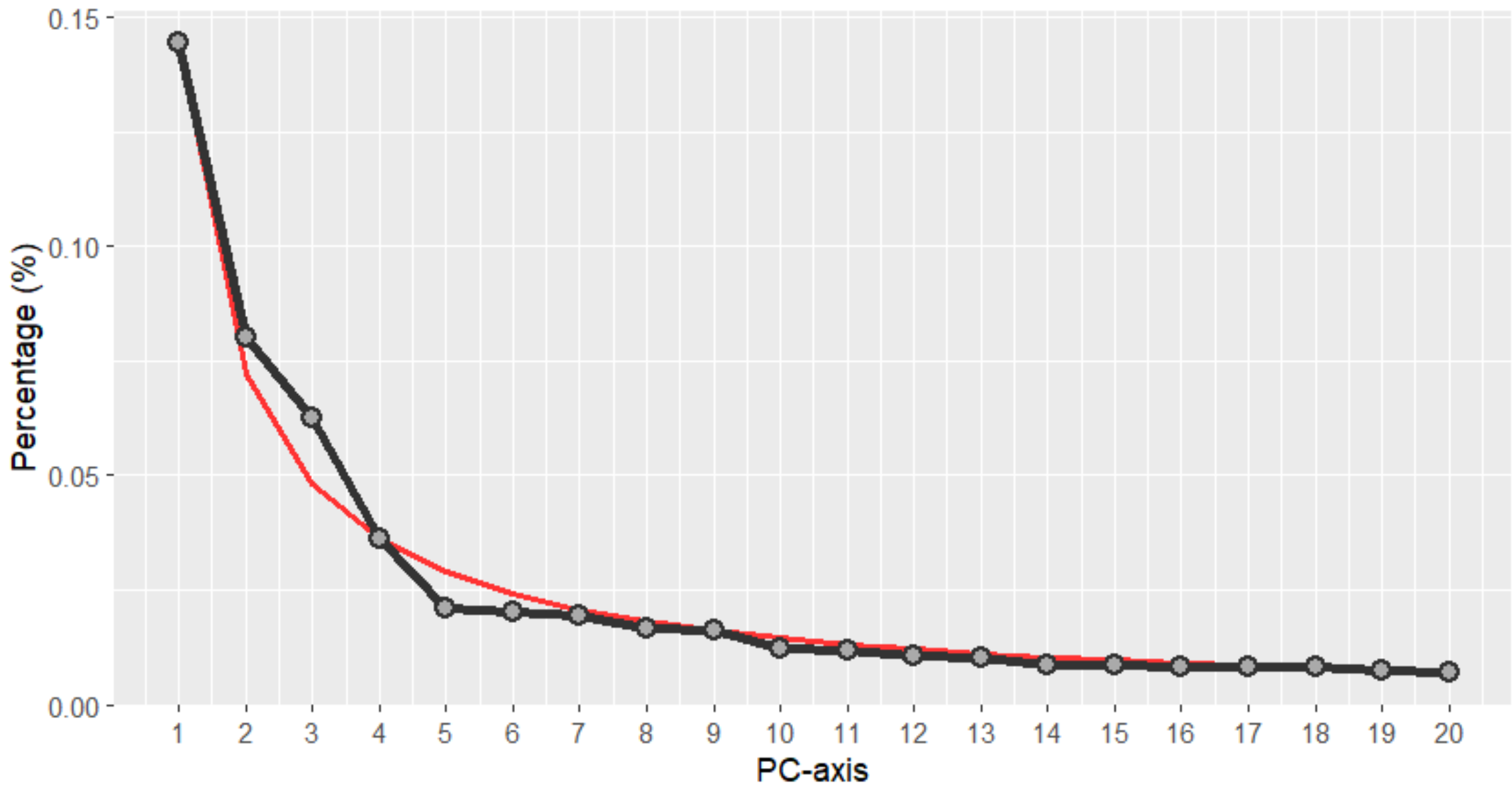

Supplementary Fig. 6: Elbow plot for identifying the optimal number of genetic clusters (*K*) based on 4,601 SNPs from 265 *Erigeron* populations obtained from ddRADseq. Principal component analysis was performed using the pca() function from LEA package, version 4.4[26], generating an object of class pcaProject containing eigenvalues, eigenvectors, and projections. The elbow plot shows the percentage of variance explained by each component, with an elbow at K = 4, suggesting four major genetic clusters. The red line visualizes the fit the geom_smooth of the elbow plot.

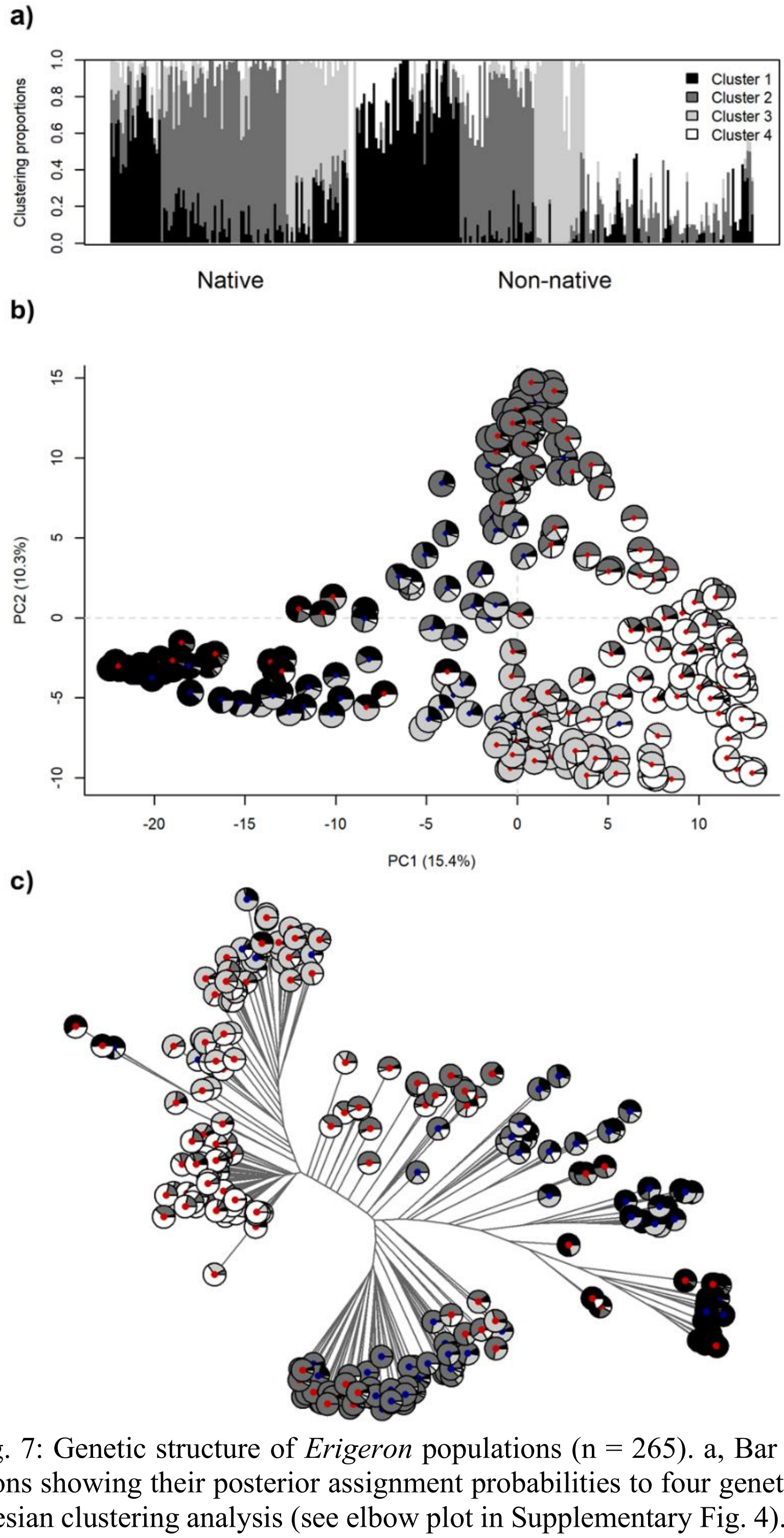

Supplementary Fig. 7: Genetic structure of *Erigeron* populations (n = 265). a, Bar plot of individuals from 265 populations showing their posterior assignment probabilities to four genetic clusters ($K = 4$), as inferred by Bayesian clustering analysis (see elbow plot in Supplementary Fig. 4). Each individual is represented by a vertical bar partitioned into four segments corresponding to cluster membership proportions. (b) Principal component analysis (PCA) of population genomic data showing the distribution of populations along the first two principal components (percentage of explained variance indicated on the axes). (c) Neighbour-joining tree based on Nei's genetic distances among populations; edge lengths reflect genetic distances. In (b) and (c), each pie chart represents a population. The colours of the inner circles indicate geographic range (native: blue; non-native: red), while the outer pie charts show cluster membership proportions: Cluster 1 (black), Cluster 2 (dark grey), Cluster 3 (light grey), and Cluster 4 (white).

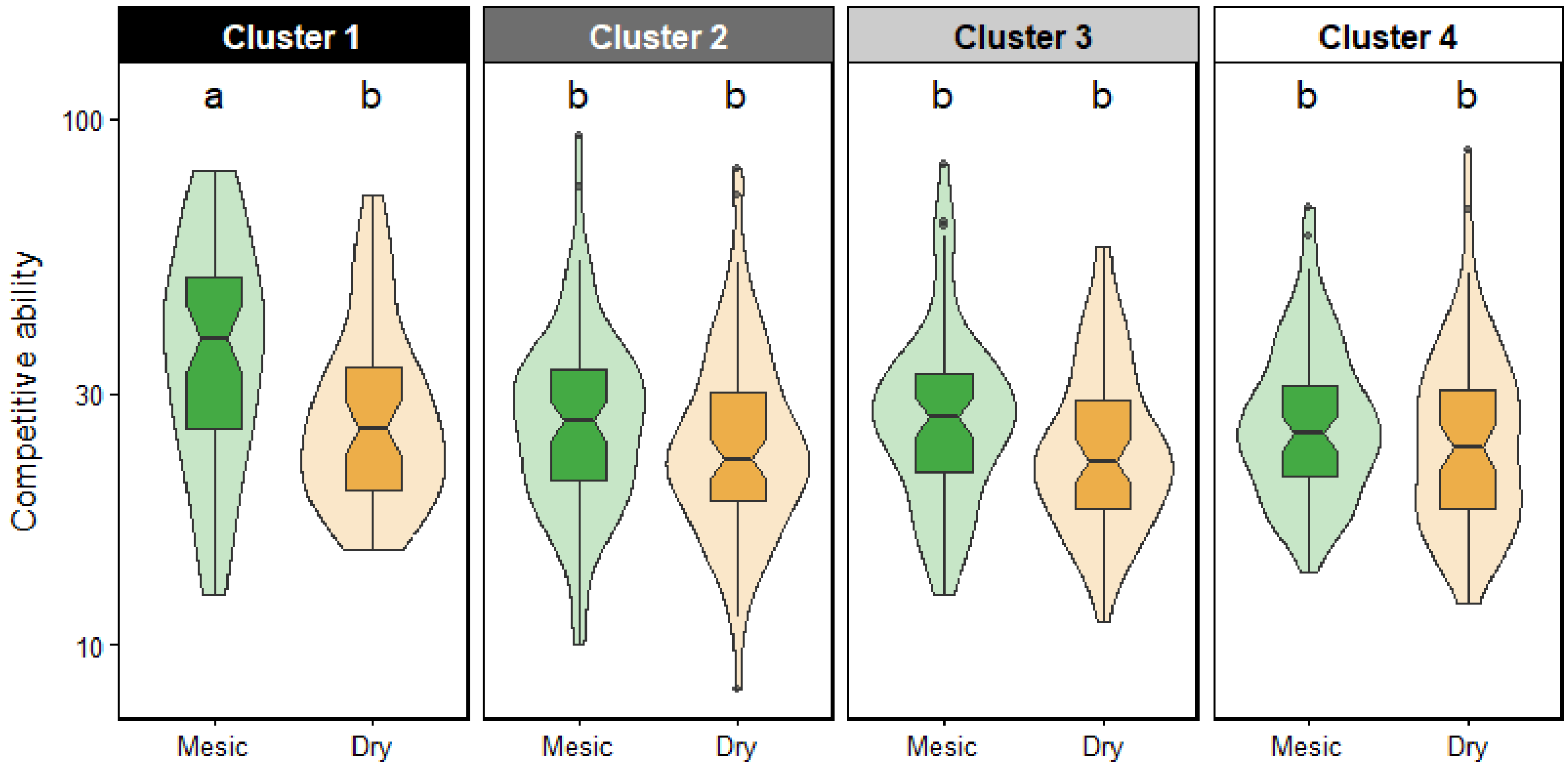

Supplementary Fig. 8: Drought response of competitive ability differs among the genetic clusters of *Erigeron* (n = 265). Competitive ability differs significantly between mesic (green) and dry (yellow) treatments but only in cluster 1 (see Supplementary Table 2 for details). Lowercase letters in a indicate groupings based on Tukey's post-hoc test. Source data are provided as a Source Data file.

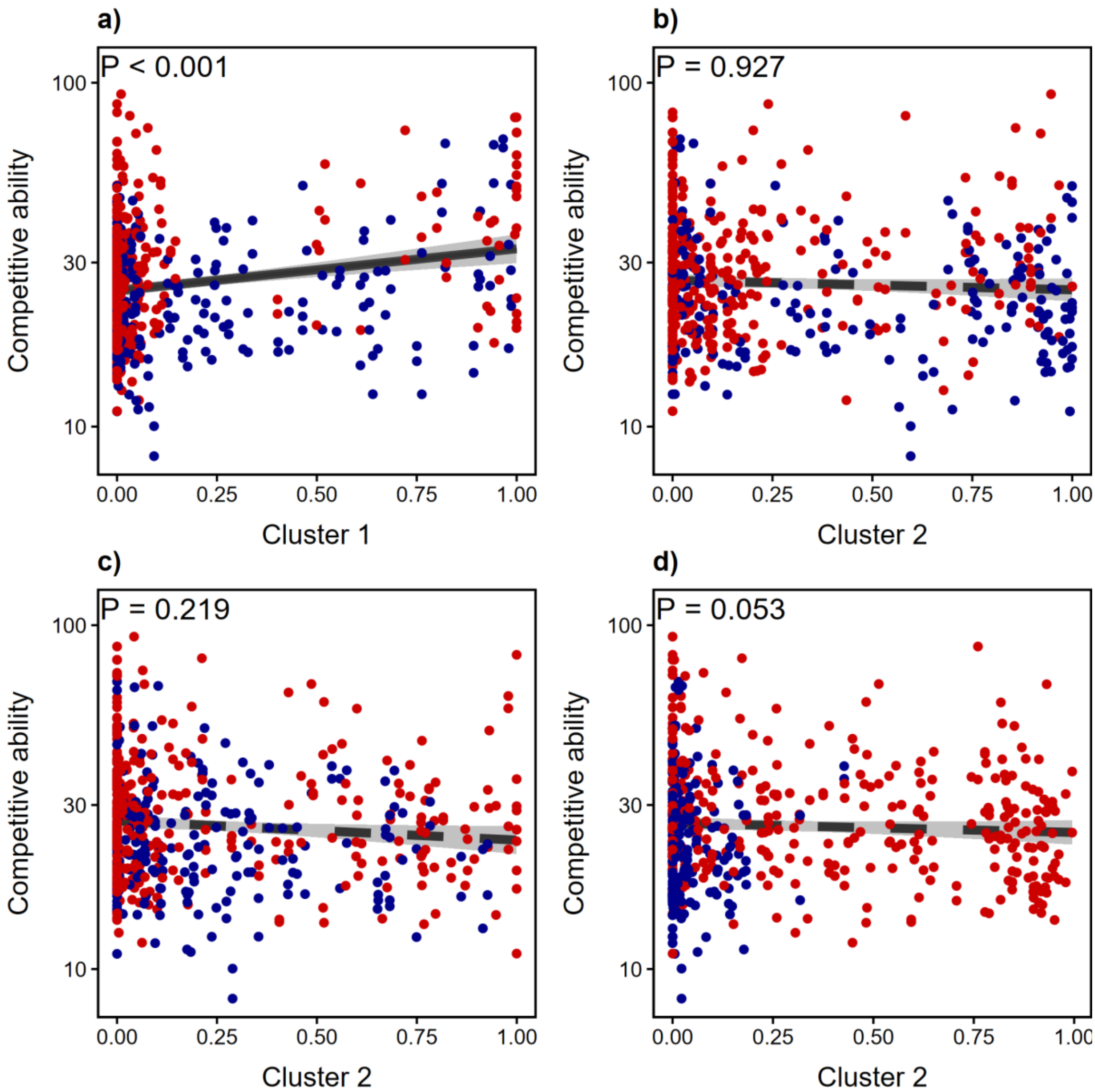

Supplementary Fig. 9: Effect of genetic clustering on the competitive ability of 100 native (blue) and 165 non-native (red) *Erigeron* populations. In contrast to the main manuscript where clustering was used as a categorical predictor (Fig. 3), here we used the assignment probability to each of the four genetic clusters as a continuous predictor of competitive ability. These analyses indicated that clusters 2, 3, and 4 (b, c, and d) did not individually influence competitive ability, whereas an increase in the clustering coefficient for cluster 1 (a) was associated with greater competitive ability. P-values refer to the results of the individual models. Linear mixed-effects models for panels a, b, c, and d only included the respective clustering coefficient without any interactions or covariates. Populations nested within region was set as a random effect. The shaded bands represent 95% confidence intervals of the linear regression. Source data are provided as a Source Data file.

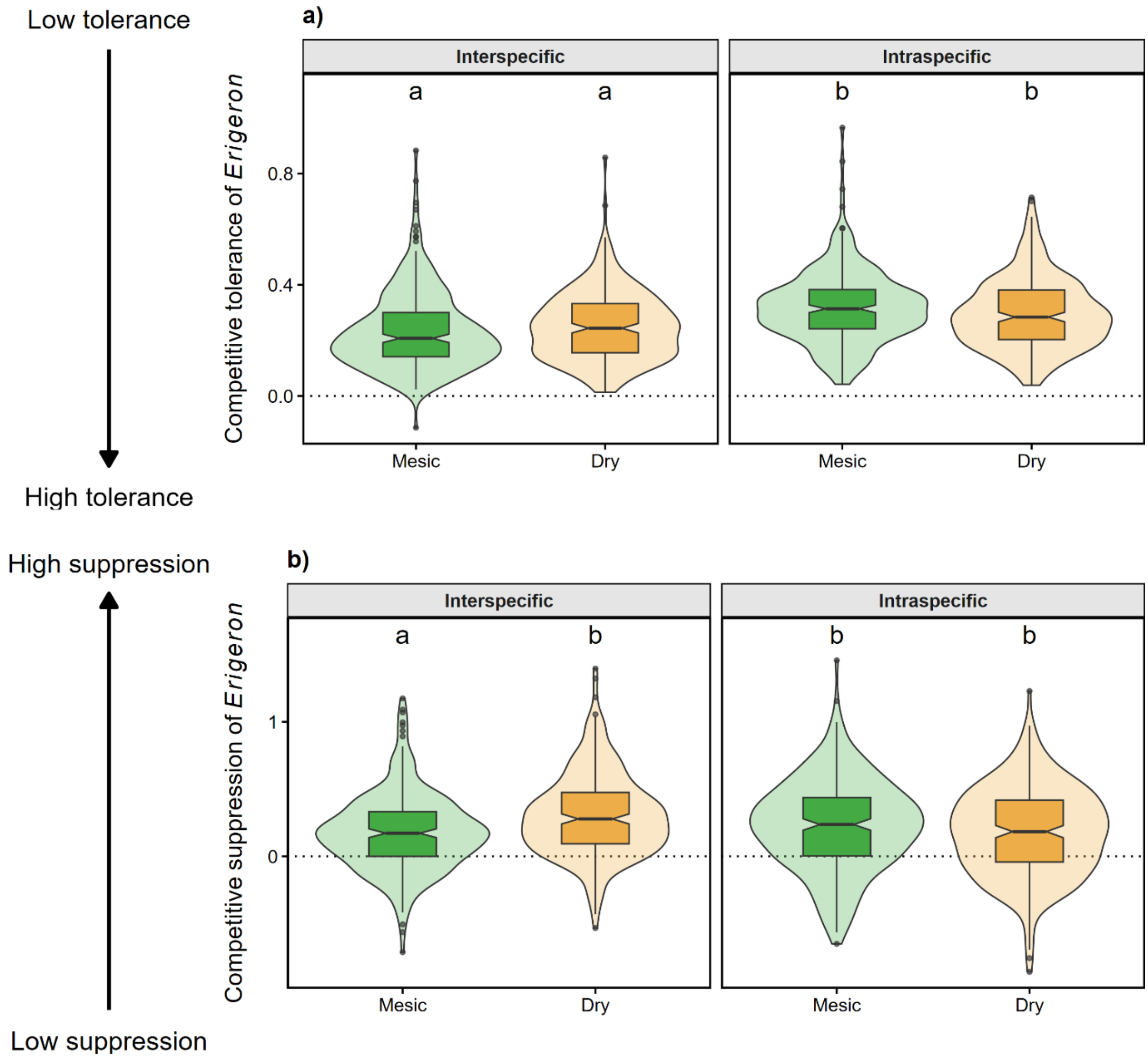

Supplementary Fig. 10: Drought response of competitive tolerance and suppression of *Erigeron* across inter- and intraspecific conditions (n = 265). a, Competitive tolerance did not differ between mesic and dry conditions under either interspecific or intraspecific competition. b, Competitive suppression significantly increased under dry compared to mesic conditions for the interspecific communities, but no such increase was observed for intraspecific communities. The increased competitive suppression under drought raises concerns that *Erigeron* may become more harmful under climate change[34]. In both panels, horizontal dashed lines at 0 indicate the threshold between competition (y > 0) and facilitation (y < 0). Arrows visualize the direction of change: an increase in competitive tolerance reflects greater effect experienced by *Erigeron* (higher values = lower tolerance), whereas an increase in competitive suppression reflects greater effect on competitor communities (higher values = higher suppression). Lowercase letters indicate groupings based on Tukey's post-hoc test. Model estimates are presented in Supplementary Table 3. Source data are provided as a Source Data file.

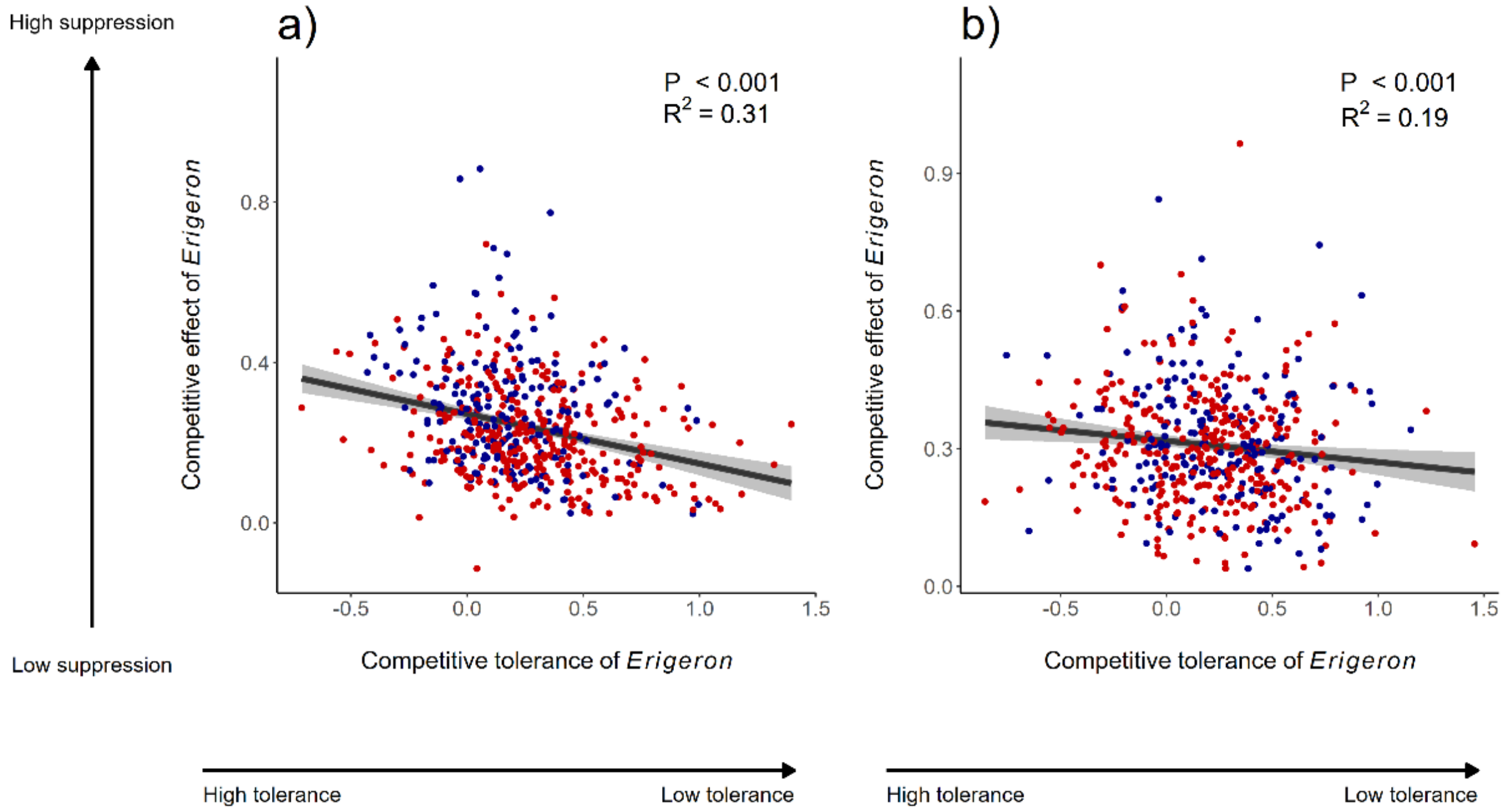

Supplementary Fig. 11: Relationship between competitive tolerance and competitive suppression across 100 native (blue) and 165 non-native (red) *Erigeron* populations with respect to the inter- (a) and intraspecific (b) competitor communities. To know if lower competitive tolerance is associated with higher competitive suppression in case of *Erigeron* populations, we tested the relationship between them in separate models. We found a significant relationship between competitive tolerance and suppression under interspecific ($\chi_{(1)}^2 = 26.17$; $P = 0.001$) and intraspecific ($\chi_{(1)}^2 = 11.07$; $P < 0.001$) conditions. The analyses revealed that as the competitive tolerance of *Erigeron* decreases, its effect lowers. In an other way, *Erigeron* populations which are less tolerant to competition, have less effect on competitors, and more tolerant populations have stronger suppression on competitors. Arrows indicate the direction of competitive tolerance and suppression: an increase in competitive tolerance reflects greater harm experienced by *Erigeron* (i.e., higher response indicates lower tolerance), whereas an increase in competitive suppression reflects greater harm caused by *Erigeron* to competitor communities. The shaded bands represent 95% confidence intervals of the linear regression. Source data are provided as a Source Data file.

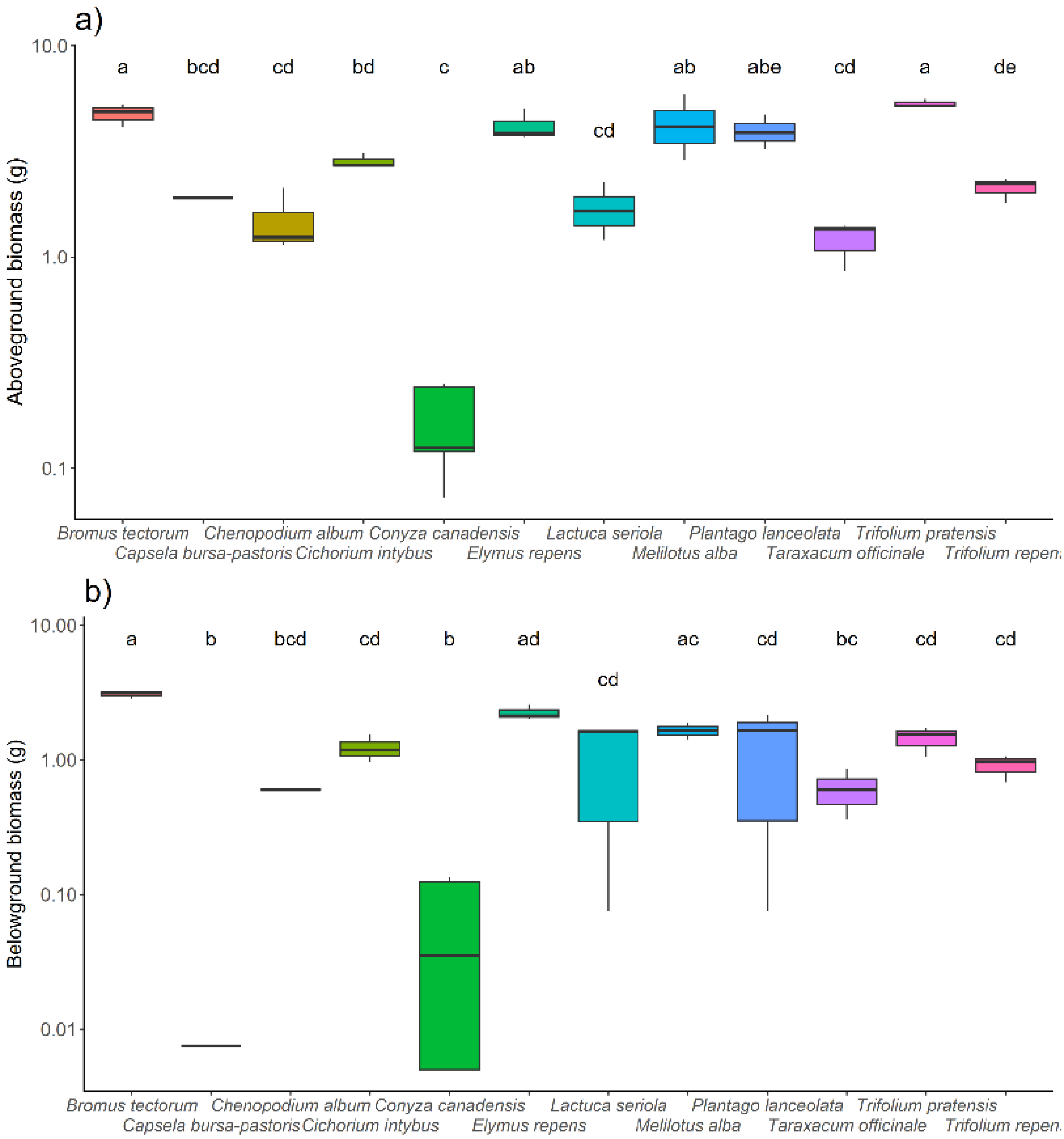

Supplementary Fig. 12: Preliminary screening of candidate competitor species. Eleven candidate competitor species were grown individually under greenhouse conditions for four weeks to assess early growth performance (n = 5 for each species). After harvest, above- and belowground biomass were measured following drying. Across species, candidate competitors produced substantially greater biomass than *Erigeron*, indicating their potential to exert competitive pressure during early developmental stages. Lowercase letters indicate groupings based on Tukey's post hoc tests. Source data are provided as a Source Data file.

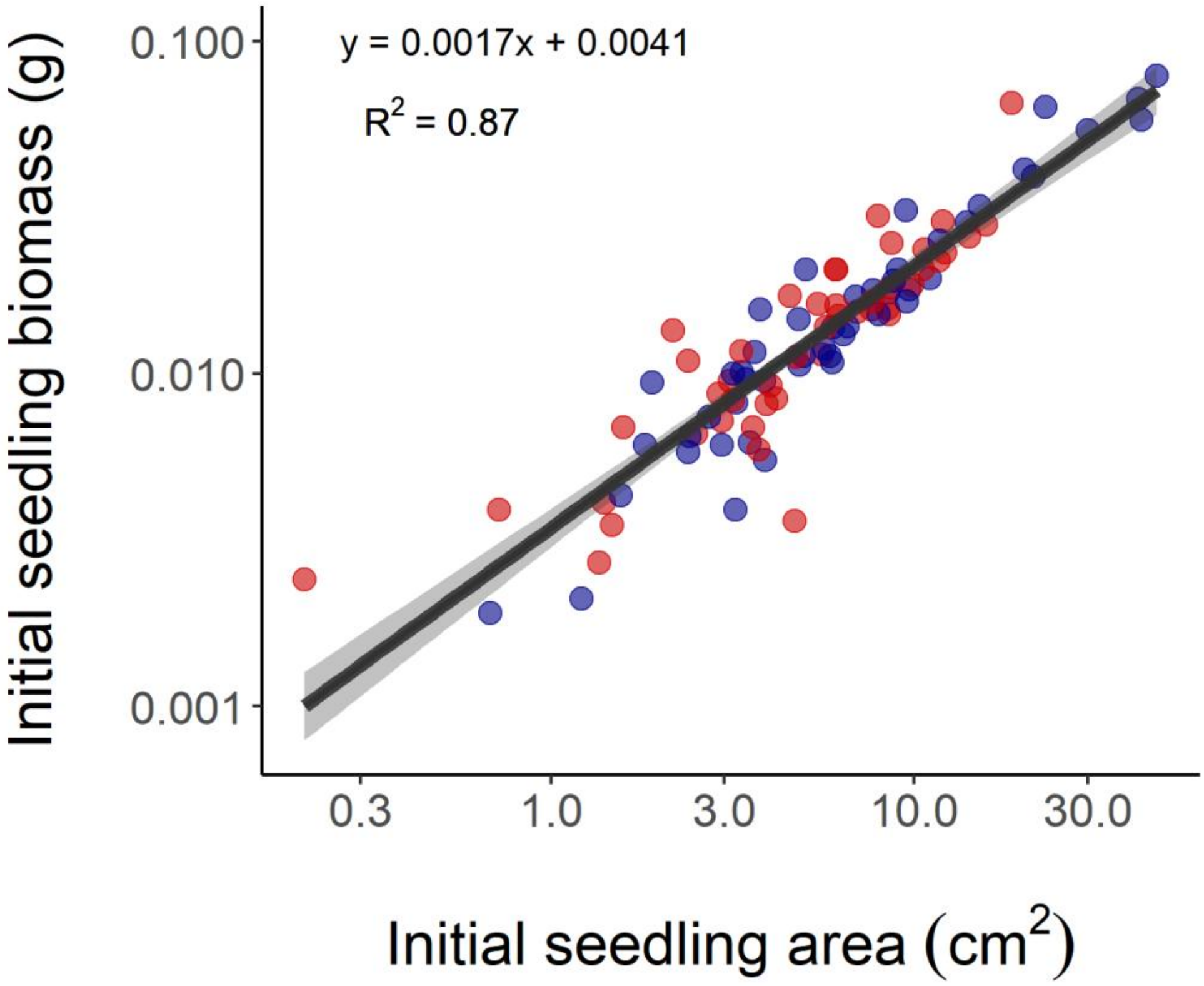

Supplementary Fig. 13: Calibration curve showing the relationship between seedling biomass and seedling area for n = 50 native (blue) and n = 47 non-native (red) *Erigeron canadensis* individuals from Ancillary Experiment 1. Plants were measured for area and harvested for biomass six weeks after sowing (Ancillary Experiment 1 in Supplementary Note 1). The resulting data were used for calculating the equation of the line (y = 0.0017x + 0.0041), where *y* is biomass and *x* is seedling area. This equation was applied to estimate initial biomass of the *Erigeron* seedlings included in the main experiment (y in the equation) by using their initial seedling area (x in the equation). The estimated initial biomass data were then used to calculate the intrinsic growth rate, which is important for estimating competitive ability[26]. The shaded band represents the 95% confidence interval of the linear regression. Source data are provided as a Source Data file.

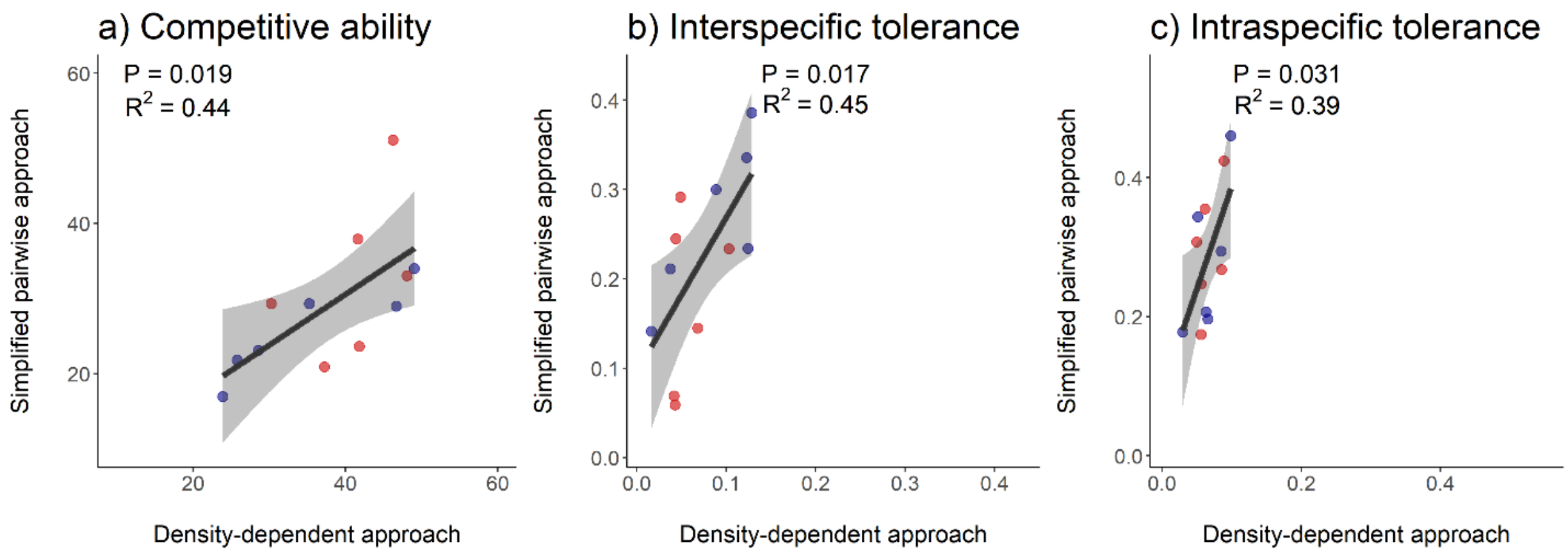

Supplementary Fig. 14: Relationship between the measures of competitive interactions in our main experiment (simplified pairwise comparison: with vs. without competitors) and those estimated using a density-dependent approach (i.e., along a competitor density gradient). The panels show the relationships for a) competitive ability, b) interspecific competitive tolerance, and c) intraspecific competitive tolerance. The estimates were compared for six populations (three native (blue) and three non-native (red) *Erigeron* populations; Supplementary Data 1) across both drought treatments (n = 12). Specifically, we compared values from the main experiment with those from a density experiment (ancillary experiment with 540 pots; Ancillary Experiment 5 in Supplementary Note 1). Importantly, both estimates capture consistent relative differences among populations. This suggests that the simplified approach does not systematically over- or underestimate competitive ability but instead provides a comparable ranking of populations while differing in absolute magnitude. The x- and y-axes show competition estimates from the density-dependent and simple pairwise approach, respectively. The P- and R^2- values represent the results of linear regression models for the comparison between the estimates from the two different experimental sources. Shaded bands represent the 95% confidence intervals of the linear regressions. Source data are provided as a Source Data file.

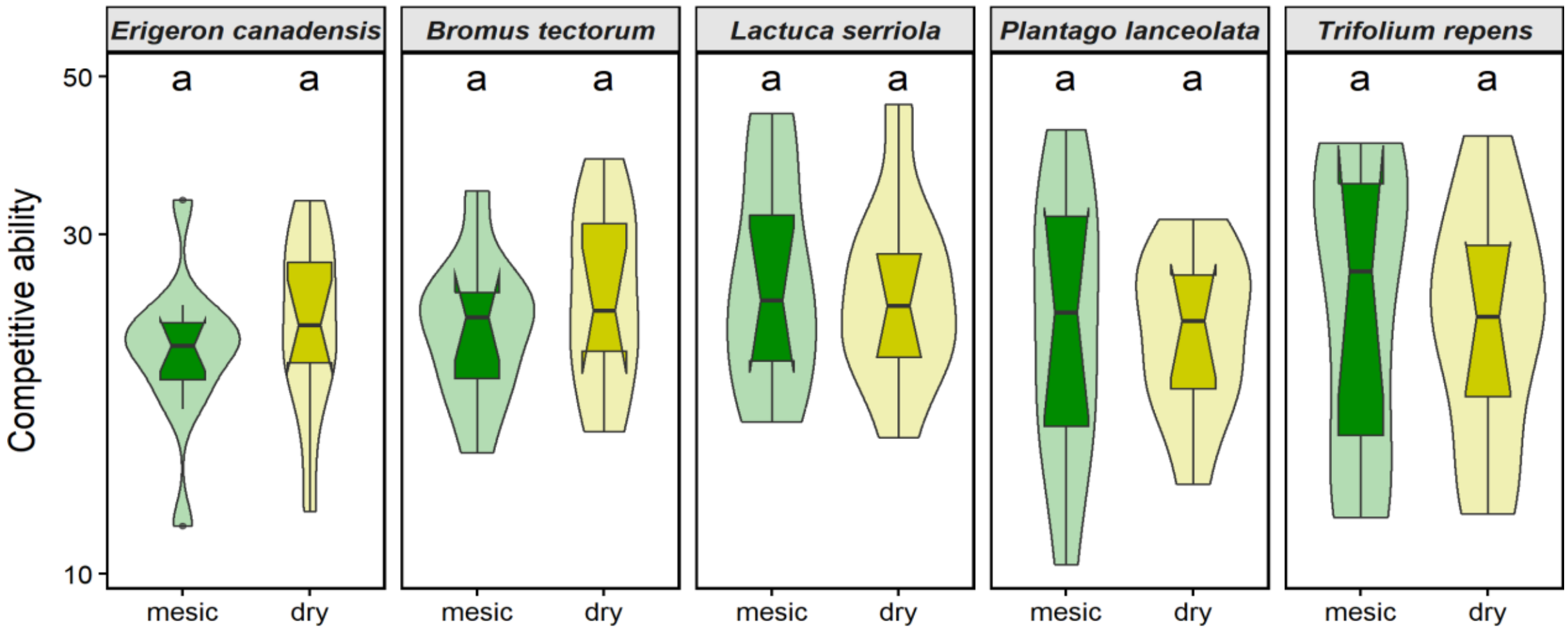

Supplementary Fig. 15: Competitive ability of the four competitor species (*Bromus tectorum*, *Lactuca serriola*, *Plantago lanceolata*, *Trifolium repens*) compared with our target species *Erigeron canadensis* under mesic (green) and dry (yellow) conditions (n = 20 for each species, except *Erigeron* for which n = 24). Competitive ability was estimated for each species using 240 pots in an ancillary experiment (Ancillary Experiment 6 in Supplementary Note 1). The effects of drought treatment and species identity were tested using a linear model. Lowercase letters indicate groupings based on pairwise comparisons, estimated using Tukey's HSD tests. Source data are provided as a Source Data file.

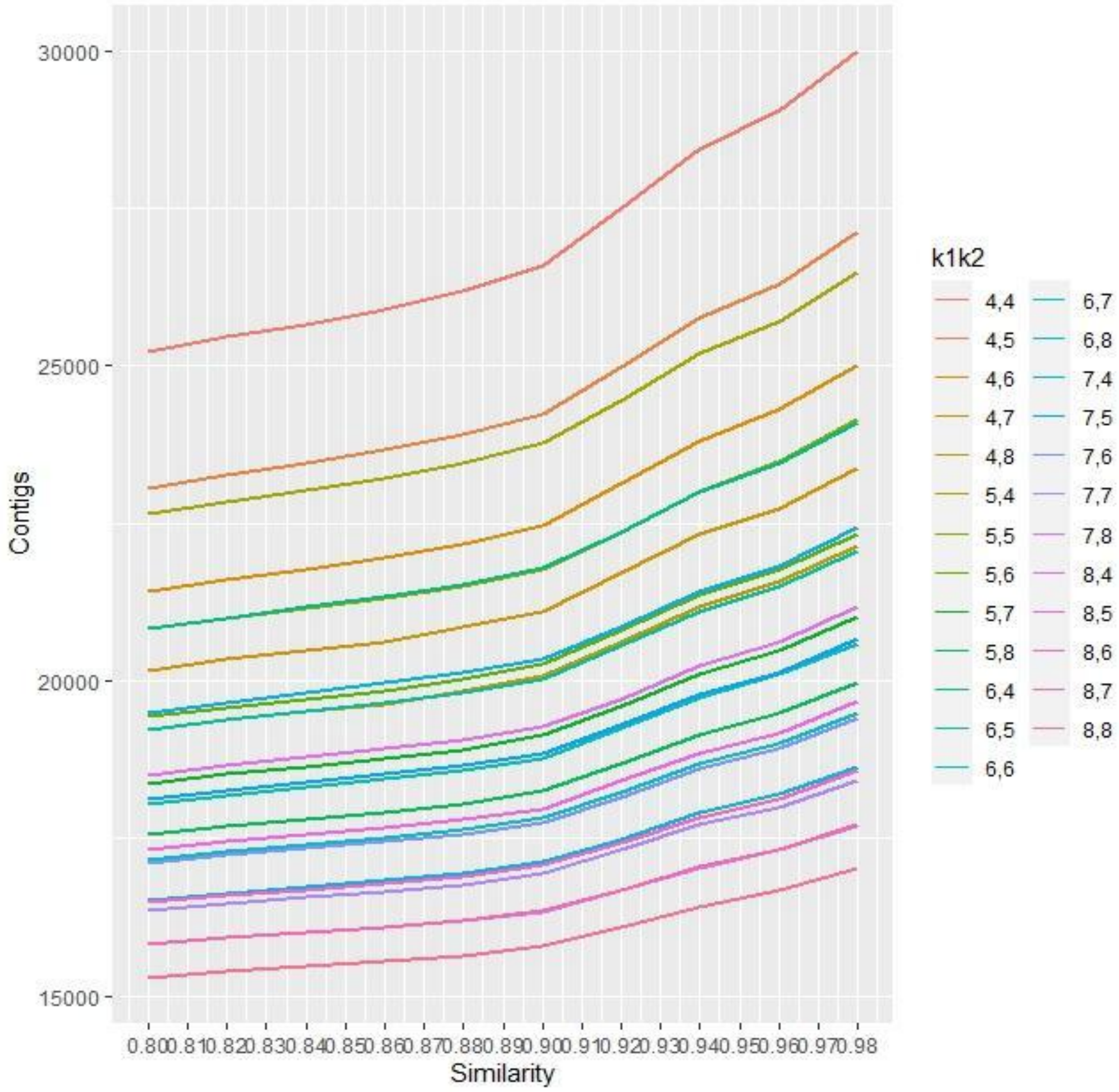

Supplementary Fig. 16: Parameter optimization for SNP genotyping with dDocent version 2.7.8[20]. Plot shows the relationship between the similarity and number of contigs for different (k1, k2) pairs. k1 and k2 represent different lengths of k-mers, i.e., substrings of a fixed length that are derived from a larger sequence. Each line represents a unique pair, with line thickness varying to indicate different levels of a specific metric. It is noted that at 0.9% similarity, the number of contigs reached its best value, indicating optimal performance at this similarity level.

SI References

1. Hart, S. P., Freckleton, R. P. & Levine, J. M. How to quantify competitive ability. *J. Ecol.* **106**, 1902–1909 (2018).

2. Mráz, P., Tarbush, E. & Müller-Schärer, H. Drought tolerance and plasticity in the invasive knapweed *Centaurea stoebe* s.l. (Asteraceae): effect of populations stronger than those of cytotype and range. *Ann. Bot.* **114**, 289–299 (2014).

3. Bennett, J. A., Riibak, K., Tamme, R., Lewis, R. J. & Pärtel, M. The reciprocal relationship between competition and intraspecific trait variation. *J. Ecol.* **104**, 1410–1420 (2016).

4. Aarssen, L. & Keogh, T. Conundrums of competitive ability in plants: what to measure? *Oikos* **96**, 531–542 (2002).

5. Aschehoug, E. T., Brooker, R., Atwater, D. Z., Maron, J. L. & Callaway, R. M. The mechanisms and consequences of interspecific competition among plants. *Annu. Rev. Ecol. Evol. Syst.* **47**, 263–281 (2016).

6. Wu, T., Li, Y., Cadotte, M., Godoy, O. & Chu, C. Can competitive effects and responses of alien and native species predict invasion outcomes? *Fundam. Res.* In press.

7. Goldberg, D. E. Competitive ability: definitions, contingency and correlated traits. *Philos. Trans. R. Soc. Lond. B Biol. Sci.* **351**, 1377–1385 (1996).

8. Atwater, D., Callaway, R. & Xiao, S. Competition as a demolition derby: why tolerating competitors is more important than suppressing them. *Oikos* **130**, 143-155 (2021).

9. Rowe, C. L. J. & Leger, E. A. Competitive seedlings and inherited traits: a test of rapid evolution of *Elymus multisetus* (big squirreltail) in response to cheatgrass invasion. *Evol. Appl.* **4**, 485–498 (2011).

10. Schwinning, S., Meckel, H., Reichmann, L., Polley, H. & Fay, P. Accelerated development in Johnsongrass seedlings (*Sorghum halepense*) suppresses the growth of native grasses through size-asymmetric competition. *PLoS ONE* **12**, e0176042 (2017).

11. Weiner, J. Weed suppression by cereals: Beyond 'competitive ability'. *Weed Res.* **63**, 133-138 (2023).

12. Zhang, Z. & van Kleunen, M. Common alien plants are more competitive than rare natives but not than common natives. *Ecol. Lett.* **22**, 1378–1386 (2018).

13. Zhang, R. & Tielbörger, K. Density-dependence tips the change of plant–plant interactions under environmental stress. *Nat. Commun.* **11**, 2532 (2020).

14. Feng, Y. et al. Inferring competitive outcomes, ranks and intransitivity from empirical data: a comparison of different methods. *Methods Ecol. Evol*. **11**, 117–128 (2020).

15. Thoma, A. E. et al. Progress and future directions of biogeographical comparisons of plant-fungal interactions in invasion contexts. *New Phytol.* **247**, 477–486 (2025).

16. Junker, R. R., Kuppler, J., Bathke, A. C., Schreyer, M. L. & Trutschnig, W. Dynamic range boxes – a robust nonparametric approach to quantify size and overlap of n-dimensional hypervolumes. *Methods Ecol Evol.* **7**, 1503–1513 (2016).

17. Peterson, B. K., Weber, J. N., Kay, E. H., Fisher, H. S. & Hoekstra, H. E. Double digest RADseq: an inexpensive method for de novo SNP discovery and genotyping in model and non-model species. *PLOS One* **7**, e37135. (2012).

18. Durka, W. et al. Assessment of genetic diversity among seed transfer zones for multiple grassland plant species across Germany. *Basic Appl. Ecol.* **84**, 50–60 (2025).

19. Catchen, J., Hohenlohe, P. A., Bassham, S., Amores, A. & Cresko, W. A. Stacks: an analysis tool set for population genomics. *Mol. Ecol.* **22**, 3124-3140 (2013).

20. Puritz, J. B., Hollenbeck, C. M. & Gold, J. R. dDocent: a RADseq, variant-calling pipeline designed for population genomics of non-model organisms. *PeerJ* **2**, e431 (2014).

21. O'Leary, S. J. et al. These aren't the loci you're looking for: principles of effective SNP filtering for molecular ecologists. *Mol. Ecol.* **27**, 3193–3206 (2018).

22. Garrison, E., Kronenberg, Z. N., Dawson, E. T., Pedersen, B. S., Prins, P. A spectrum of free software tools for processing the VCF variant call format: vcflib, bio-vcf, cyvcf2, hts-nim and slivar. *PLoS Comput. Biol*. **18**, e1009123 (2022).

23. Danecek, P, et al. The variant call format and VCFtools. *Bioinformatics* (2011)

24. Gruber, B., Unmack, P. J., Berry, O. F. & Georges, A. dartr: An r package to facilitate analysis of SNP data generated from reduced representation genome sequencing. *Mol. Ecol. Resour.* **18**, 691-699 (2018).

25. Luu, K., Bazin, E. & Blum, M. G. B. pcadapt: an R package to perform genome scans for selection based on principal component analysis. *Mol. Ecol. Resour.* **17**, 67–77 (2016).

26. Frichot, E. & François, O. LEA: an R package for landscape and ecological association studies. *Methods Ecol. Evol.* **6**, 925–929 (2015).

27. Hijmans, R. J., Cameron, S. E., Parra, J. L., Jones, P. G., Jarvis, A. Very high resolution interpolated climate surfaces for global land areas. *Int. J. Climatol.* **25**, 1965–1978 (2005).

28. Abatzoglou, J. T., Dobrowski, S. Z., Parks, S. A. & Hegewisch, K. C. Terraclimate, a high-resolution global dataset of monthly climate and climatic water balance from 1958–2015. *Sci. Data* **5**, 170191 (2018).

29. Benjamini, Y. & Hochberg, Y. Controlling the false discovery rate: a practical and powerful approach to multiple testing. *J. R. Stat. Soc.* B **57**, 289–300 (1995).

30. Capblancq, T., Luu, K., Blum, M. G. B. & Bazin, E. Evaluation of redundancy analysis to identify signature of local adaptation. *Mol. Ecol. Resour.* **18**, 1223–1233 (2018).

31. François, O., Martins, H., Caye, K. & Schoville, S. D. Controlling false discoveries in genome scans for selection. *Mol. Ecol.* **25**, 454–469 (2016).

32. Lachmuth, S., Durka, W. & Schurr, F. M. Differentiation of reproductive and competitive ability in the invaded range of *Senecio inaequidens*: the role of genetic Allee effects, adaptive and nonadaptive evolution. *New Phytol.* **192**, 529–541 (2011).

33. Rosche, C. et al. Climate outweighs native vs. nonnative range-effects for genetics and common garden performance of a cosmopolitan weed. *Ecol. Monogr.* **89**, e01386 (2019).

34. Nagy, D. U. et al. Among-population variation in drought responses is consistent across life stages but not between native and non-native ranges. *New Phytol.* **243**, 922–935 (2024).
