# Supplementary material for: Rapid and repeated evolution of increased competitive ability in a global invader
Source: Nat Commun. 2026 Sep 17;17:9930. doi: 10.1038/s41467-026-77819-z (PMC13586298; doi:10.1038/s41467-026-77819-z)
Supplement: Supplementary file 2 [file 41467_2026_77819_MOESM2_ESM.pdf]

Description of Additional Supplementary Files

File Name: Supplementary Data 1

Description: Includes information on every population used in this study. The data file includes population codes, names, coordinates, genetic clustering and environmental data used, together with the usage of populations in specific experiments.

File Name: Supplementary Data 2

Description: The list of identifiers of the individuals used in this experiment for the raw sequence data stored in the European Nucleotide Archive (ENA).
