# Supplementary material for: Rapid and repeated evolution of increased competitive ability in a global invader
Source: Nat Commun. 2026 Sep 17;17:9930. doi: 10.1038/s41467-026-77819-z (PMC13586298; doi:10.1038/s41467-026-77819-z)
Supplement: Supplementary file 3 [file 41467_2026_77819_MOESM5_ESM.pdf]

Corresponding author(s): DBPR: NCOMMS-25-78468B

Last updated by author(s): Jul 31, 2026

Reporting Summary

Nature Portfolio wishes to improve the reproducibility of the work that we publish. This form provides structure for consistency and transparency in reporting. For further information on Nature Portfolio policies, see our Editorial Policies and the Editorial Policy Checklist.

Statistics

For all statistical analyses, confirm that the following items are present in the figure legend, table legend, main text, or Methods section.

n/a	Confirmed	
☐	☒	The exact sample size (*n*) for each experimental group/condition, given as a discrete number and unit of measurement
☐	☒	A statement on whether measurements were taken from distinct samples or whether the same sample was measured repeatedly
☐	☒	The statistical test(s) used AND whether they are one- or two-sided<br>*Only common tests should be described solely by name; describe more complex techniques in the Methods section.*
☐	☒	A description of all covariates tested
☐	☒	A description of any assumptions or corrections, such as tests of normality and adjustment for multiple comparisons
☐	☒	A full description of the statistical parameters including central tendency (e.g. means) or other basic estimates (e.g. regression coefficient) AND variation (e.g. standard deviation) or associated estimates of uncertainty (e.g. confidence intervals)
☐	☒	For null hypothesis testing, the test statistic (e.g. *F*, *t*, *r*) with confidence intervals, effect sizes, degrees of freedom and *P* value noted<br>*Give P values as exact values whenever suitable.*
☒	☐	For Bayesian analysis, information on the choice of priors and Markov chain Monte Carlo settings
☐	☒	For hierarchical and complex designs, identification of the appropriate level for tests and full reporting of outcomes
☒	☐	Estimates of effect sizes (e.g. Cohen's *d*, Pearson's *r*), indicating how they were calculated

Our web collection on statistics for biologists contains articles on many of the points above.

Software and code

Policy information about availability of computer code

Data collection	No software used for data collection.
Data analysis	All statistical analyses were performed in R (4.3.3; R Core Team, 2024). All analyses used published R packages, as detailed in the Methods section (e.g., lme4, coxme, pedigreemm, LEA, dartR, ncf). All data and R scripts supporting the results, including model code, are archived at https://doi.org/10.6084/m9.figshare.29881193

For manuscripts utilizing custom algorithms or software that are central to the research but not yet described in published literature, software must be made available to editors and reviewers. We strongly encourage code deposition in a community repository (e.g. GitHub). See the Nature Portfolio guidelines for submitting code & software for further information.

Data

Policy information about availability of data

All manuscripts must include a data availability statement. This statement should provide the following information, where applicable:

- Accession codes, unique identifiers, or web links for publicly available datasets
- A description of any restrictions on data availability
- For clinical datasets or third party data, please ensure that the statement adheres to our policy

The processed data are on the Figshare Repository at the following link:https://doi.org/10.6084/m9.figshare.29881193. Climate data have been extracted from TerraClimate at https://www.climatologylab.org/terraclimate.html.

Research involving human participants, their data, or biological material

Policy information about studies with human participants or human data. See also policy information about sex, gender (identity/presentation), and sexual orientation and race, ethnicity and racism.

Reporting on sex and gender	N/A
Reporting on race, ethnicity, or other socially relevant groupings	N/A
Population characteristics	N/A
Recruitment	N/A
Ethics oversight	N/A

Note that full information on the approval of the study protocol must also be provided in the manuscript.

Field-specific reporting

Please select the one below that is the best fit for your research. If you are not sure, read the appropriate sections before making your selection.

☐ Life sciences ☐ Behavioural & social sciences ☒ Ecological, evolutionary & environmental sciences

For a reference copy of the document with all sections, see nature.com/documents/nr-reporting-summary-flat.pdf

Ecological, evolutionary & environmental sciences study design

All studies must disclose on these points even when the disclosure is negative.

Study description	We used a full factorial design of three competition treatments (alone; interspecific with four heterospecifics; intraspecific with four conspecifics) with two water regimes (mesic vs. dry), testing all combinations per population. The experimental unit was a single pot containing one focal Erigeron canadensis individual (plus competitors depending on treatment). Each population × treatment combination was replicated twice, yielding 3,408 experimental units in the glasshouse.
Research sample	The research sample consisted of 285 globally distributed populations of Erigeron canadensis (syn. Conyza canadensis), a cosmopolitan plant species that serves as a powerful model for studying rapid evolution. All data used in this study were derived from the glasshouse experiment described above. To characterize the local aridity regime experienced by each population in the field, we extracted climatic water deficit values from the TerraClimate dataset.
Sampling strategy	Sampling was done by harvesting the aboveground biomass of all pot included in the experiment.
Data collection	The biomass data was measured by the authors, using precision scales. Population genetic data was assessed using ddRAD sequencing method.
Timing and spatial scale	The glasshouse experiment was conducted from March to July 2022. Biomass measurements and genetic analyses based on the resulting material were completed between late 2022 and mid-2023.
Data exclusions	During the glasshouse experiment, 19 out of 284 populations were excluded due to low germination or poor seedling survival, resulting in 265 populations included in the final statistical analyses (see Supplementary Table 1 for details)
Reproducibility	All data and code required to reproduce the analyses, including raw glasshouse measurements, calculated competitive ability indices, the genetic relatedness matrix, and all R scripts used for statistical analyses, are available at https://doi.org/10.6084/m9.figshare.29881193. Raw sequencing data are also deposited as described in the Data Availability section, enabling full reproducibility of the results.
Randomization	The pots in the experiment were randomized three times a week in the glasshouse. For details, see Methods/Experimental set up section.
Blinding	It is not relevant to our study since all measurements (e.g., biomass measurements) were based on objective quantitative data rather than subjective assessment.

Did the study involve field work? ☒ Yes ☐ No

Field work, collection and transport

Field conditions	Field work had been conducted on private property or public land, i.e., not protected areas.

Location	Seeds of Erigeron canadensis were collected from 285 populations spanning both the native and non-native ranges of the species. The exact collection localities, including geographic coordinates and regional assignments, are provided in Supplementary Table 1.
Access & import/export	Export permits were obtained for each country when required. Erigeron is not under protection in any country where seeds were collected. There are no specific import regulations as this species is native to USA.
Disturbance	This study did not cause environmental disturbance.

Reporting for specific materials, systems and methods

We require information from authors about some types of materials, experimental systems and methods used in many studies. Here, indicate whether each material, system or method listed is relevant to your study. If you are not sure if a list item applies to your research, read the appropriate section before selecting a response.

Materials & experimental systems

n/a	Involved in the study	
☒	☐	Antibodies
☒	☐	Eukaryotic cell lines
☒	☐	Palaeontology and archaeology
☒	☐	Animals and other organisms
☒	☐	Clinical data
☒	☐	Dual use research of concern
☐	☒	Plants

Methods

n/a	Involved in the study	
☒	☐	ChIP-seq
☒	☐	Flow cytometry
☒	☐	MRI-based neuroimaging

Dual use research of concern

Policy information about dual use research of concern

Hazards

Could the accidental, deliberate or reckless misuse of agents or technologies generated in the work, or the application of information presented in the manuscript, pose a threat to:

No	Yes	
☒	☐	Public health
☒	☐	National security
☒	☐	Crops and/or livestock
☒	☐	Ecosystems
☒	☐	Any other significant area

Experiments of concern

Does the work involve any of these experiments of concern:

No	Yes	
☒	☐	Demonstrate how to render a vaccine ineffective
☒	☐	Confer resistance to therapeutically useful antibiotics or antiviral agents
☒	☐	Enhance the virulence of a pathogen or render a nonpathogen virulent
☒	☐	Increase transmissibility of a pathogen
☒	☐	Alter the host range of a pathogen
☒	☐	Enable evasion of diagnostic/detection modalities
☒	☐	Enable the weaponization of a biological agent or toxin
☒	☐	Any other potentially harmful combination of experiments and agents

Plants

Seed stocks	Seeds of Erigeron canadensis were collected from 285 populations spanning both the native and non-native ranges of the species. The exact collection localities, including geographic coordinates and regional assignments, are provided in Supplementary Table 1.
Novel plant genotypes	N/A
Authentication	N/A
