# Supplementary material for: Rapid and repeated evolution of increased competitive ability in a global invader
Source: Nat Commun. 2026 Sep 17;17:9930. doi: 10.1038/s41467-026-77819-z (PMC13586298; doi:10.1038/s41467-026-77819-z)
Supplement: Supplementary file 4 [file 41467_2026_77819_MOESM6_ESM.pdf]

nature portfolio

Peer Review File

Rapid and repeated evolution of increased competitive ability in a global invader

Corresponding Author: Dr Dávid Nagy

This file contains all reviewer reports in order by version, followed by all author rebuttals in order by version.

Version 0:

Reviewer comments:

Reviewer #1

(Remarks to the Author)

This work was a pleasure to review. It is of exceptional quality in testing the EICA hypothesis. Previous studies often relied on limited sampling sizes (often 10 or fewer populations with limited geographic reach), used a simple measure of biomass as indication of competitive ability. Low sample sizes often resulted in outcomes that were contradictory. The authors here overcome many of these problems by having enormous sample sizes in native and non-native regions, directly test both intra- and interspecific competition under two different scenarios (mesic, dry) using appropriate experimental designs, and add genetic data to their analyses. Their results are convincing and the MS is easy to read and appropriately illustrated. The Supplementary Information is expansive and supports the main conclusions using a serious of supporting experiments

I have few suggestions for the authors, nothing major to critique. I am not familiar with the genetics work, so I am unable to critically address that part of the investigation. Here are a few issues I like the authors to address/correct:
1. I like for the authors to sharpen their questions or hypotheses. As written in Q1 and Q3, they are not addressing a single question, but often a complex of interactions. Maybe hypotheses would be more appropriate?
2. I am concerned about collecting seeds from only a single individual in the field. There is no description on how these individuals were selected.
3. While extensive, the experiments were done in very small pots, considering that up to 5 individuals of different species were grown for several months. The pictures in the Supplemental show seedlings. Would plants become pot-bound, or resource limited? I don't think there are pictures of the full sized or terminal pots that the authors make available.
4. I like to see a discussion of how choice of the experimental organism as an anuual may influence the findings. Is the ability of an annual plant to respond to changes in selection pressure elevated compare dot species that are more long-lived? On the other hand, if Erigeron is mostly self pollinating, as claimed, how can we expect such rapid evolution to occur? These topics appear less well-developed in the discussion.

5. In Supplementary Fig. 6 the panels are not appropriately labeled. There are 3 (b).
6. In Supplementary Fig.9 the graphs use Conyza instead of Erigeron

Reviewer #2

(Remarks to the Author)
Assessing the mechanisms driving success of invasive species in their new ranges has been a goal in ecological research for several decades. One prominent hypothesis (proposed in 1995) is the evolution of increased competitive abilities (EICA) hypothesis (Blossey and Notzold, J of Ecology). In this current manuscript, the authors evaluate one part of this hypothesis (that of increased competitive ability in the introduced range) with a very large experiment in greenhouse mesocosms with ~250 populations of an invasive plant species collected from across both the native and ranges, coupled with genetic analyses to assess population relatedness. They find that individuals from populations in the introduced range perform better than those from the native range when grown in competition in their experimental setup. The scale of the populations sampled to include in the experiment is remarkable, and the main and supporting greenhouse experiments are substantial.

While I appreciate the tremendous amount of work that went into the experiments, including all of the ancillary experiments, overall, I disagree that the results support the interpretation as presented. A full assessment of limitations and stronger engagement with the literature would help put the paper more firmly and in context and make the interpretations more believable.

Here I provide more detail about my concerns along with suggestions for improving the manuscript.

1. The first paragraph of the introduction oversimplifies earlier research. In particular, the original EICA hypothesis was proposed as increased competitive ability in the introduced range arising from escape from enemies leading to a reallocation of resources from defense to growth. It is a difficult hypothesis to test given the multiple pathways that it includes (even as many, many common garden experiments have been done to evaluate one or several components of it). Here the authors instead cite the hypothesis as increased competitive ability as arising from selection pressures from new neighbors in the introduced range. This is both an incorrect citation of the original 1995 paper, and I think also gets at one of my core concerns about the manuscript. I agree that the current study definitively shows that plants from invasive populations can grow larger across a range of conditions (drought, wet, different densities and compositions of neighbors), but I am not convinced the design is sufficient to evaluate whether it is selection from neighbors that is the mechanism driving the changes in traits.

2. While the genetic analyses seem key to ruling out some of the alternative hypotheses, the story seems more complex than described here. The main result that introduced pops have higher competitive ability is only supported by clusters 1 and 2, and cluster 4 (which is not present in the native range), actually has the lowest competitive ability. I would thus like to see the results presented with more cautionary language, and acknowledge the pieces of them that point in a different direction. It also seems important to compare the results (split by genetic clusters) in the drought treatment vs. control in the main text.

3. How were these 4 competitor species chosen? Where did the seeds for them come from? What does it mean that they are species that Erigeron did not evolve with? The justification in the text is not very clear, but I think their identities are critical. It doesn't seem like the neighbor species provide a particularly competitive environment. Two of them are perennials, and we would expect them to grow more slowly than the focal annual species, and the focal species can grow to 2 m, much larger than any of the others. In Suppl Fig 1, it is already quite apparent that the focal species is much larger than any of the others, which don't seem that likely to be providing much competition, even if they're all rather weedy species. I would like more context provided, and a consideration of how greenhouse results might or might translate to the field.

4. The conditions in which the plants were grown matter. We know this from common garden studies in general, and more specifically from studies that have conducted multiple common gardens of the same invasive species (e.g. Coulatti et al 2009, Evol. Applications & 2016 book chapter in Invasion Genetics; Williams et al. 2008, Oecologia). Tell us what soil was used, what day length, temperature, humidity and supplemental lighting in the greenhouse were used – and explain how the choices were made. In particular – despite the drought treatment, are these plants growing in a nutrient rich environment? Presumably with no soil biota?

5. The literature on competition and coexistence is entirely overlooked beyond the citation of the Hart et al paper, and I think provides important context, and needs to be engaged with. For example, we know that competitive ability depends on the environment (e.g. Van Dyke et al 2022, Nature; Wainwright et al 2019, J Ecol) and more recent work has considered evolution in this context (e.g. Germain et al. 2023 J Ecol). Engagement with this literature particularly in the intro (L44-68) and then in the interpretation of the results would help with the context.

6. I found the conclusion on L268 that no trade-off between drought and competitive ability means the invaders are more likely to win as a large stretch. (I encourage the authors to double check the references throughout this paragraph– to me they are not the most appropriate refs for the conclusions. e.g. why ref 58 to support sentence 1 – a meta-analysis or EICA that isn't acknowledge earlier). Also, even though Suppl Fig 3 shows a significant positive relationship in competitive ability measured in mesic and dry conditions, it is not a strong relationship: the slope of the line does not appear to be that different from zero (would help if the figure were square, and the R2 and slope reported), and there is a ton of variability. I would tread carefully.

7. Ancillary experiment 5 is critical to interpreting these results, but more care needs to be taken in interpreting the results. It is difficult to examine the strength of the correlation, because the axes are on different scales (and the figs are not square). Does the experiment with one density over or underestimating competitive ability? Shouldn't these results lead to more caution in the absolute values reported in the main text?

A few more minor comments:
8. The study system needs more details. What is the invasion history of Erigeron? When was it introduced to Eurasia? Do we know anything about the invasion pathway? What kind of habitat does it occur in? (My impression is that it's a species that's adapted to disturbance, which provides a different lens to consider competition in). Does the community biomass shown in Fig 1 include Erigeron?

9. L438 – competitive ability of which species?

10. L 465 – I must have missed the reporting of these results, but this is repeated measurements and running a series of t-tests is not appropriate

11. The physiology measurements are not well incorporated in the manuscript – could be dropped.

12. I think Extended data fig 2 is important – it is critical to see the raw data not just the estimates of competition coefficients, and I think worth reporting at least something about the magnitude of seed production reduction in the main text (not just comparing competitive abilities). That said, I was surprised how small these plants are, and even more surprised that a plant with 1 g of dry weight could have 300 capitula.

Reviewer #3

(Remarks to the Author)
In this manuscript, the authors describe a glasshouse experiment of impressive scale utilizing the invasive weed Erigeron canadensis. Using hundreds of populations spanning native and invaded ranges and exposed to mesic and dry conditions, they investigate competitive ability while accounting for population ancestry — an important but still relatively rare consideration in experiments of this scale.

The authors report increased competitive ability in invaded populations relative to native populations, regardless of drought stress. At the level of genetic clusters (based on ADMIXTURE analysis of ddRADseq genotypes), two clusters showed increased competitive ability in the invaded range, a third did not show a significant difference, and a fourth contained only representatives of the invaded range. Using separate competition treatments, they determine that the increased competitive ability arises from interspecific rather than intraspecific interactions.

I found the manuscript to be well written and carefully edited, with clear figures and, for the most part, results that support the authors' conclusions. The study provides strong empirical evidence for a long-hypothesized feature of invasive species. Given the scale of the data strength of the results, I believe it would make a solid contribution to the Nature Communications. However, I have several comments that I believe would strengthen the manuscript:

1. It is somewhat unexpected that genetic cluster 4 is not represented in the native range, given the authors' assertion that the native range was comprehensively sampled. Could cluster 4 represent admixture or post-introduction divergence within the invaded range? Additional visualization of genetic structure (e.g., a PCA showing cluster assignment and native vs. invaded) would help clarify the relationships among clusters and support the interpretation of evolutionary independence.

2. The authors interpret increased competitive ability in two introduced clusters as evidence for multiple independent evolutionary origins of this trait. However, it would be helpful to quantify the extent of gene flow among clusters within the invaded range. If substantial gene flow has occurred, elevated competitive ability in multiple clusters could reflect introgression from a single origin rather than independent evolution. Providing estimates of inter-cluster gene flow would strengthen the inference of repeated evolution.

3. The authors account for population co-ancestry using pedigree mixed-effects models in the primary analyses. However, when examining how competition varied among genetic clusters, the authors revert to standard mixed models including only admixture cluster as a fixed explanatory variable. It is unclear why this was done but it would be helpful to clarify whether the results are robust to modelling continuous relatedness rather than discrete cluster identity alone.

4. It is not clear whether maternal environmental effects were controlled (e.g., via a common-garden generation). A brief clarification or discussion of the potential influence of maternal effects would further strengthen the evolutionary interpretation.

5. In Fig. 4 I suggest re-labelling the y-axes "Competitive…of Erigeron" or just drop "of Conyza" altogether.

Version 1:

Reviewer comments:

Reviewer #1

(Remarks to the Author)
I have reviewed the changes the authors have made and these have improved the manuscript. I appreciate the level of detail the authors have gone to in explaining their reasoning and the changes they made.

Reviewer #2

(Remarks to the Author)
In their revision, the authors have now more carefully framed their manuscript, and take somewhat more care in distinguishing their hypotheses and questions from the EICA hypothesis. They have addressed most of my concerns, along with those of the other reviewers. Below I bring back a few concerns I think still need addressing and one major point on the framing.

Major point on framing:
Throughout the introduction and discussion, the authors emphasize that their study is more rigorous and complete than any

that have come before. While I appreciate the tremendous effort that went into the experiments and the genomics, in general, just doing a bigger experiment does not necessarily make a study a better contribution. I would be more compelled if this language were used more sparingly, particularly in the discussion. Dozens if not hundreds of studies have examined the possibility that invasive species are more competitive where they are introduced, and we have learned quite a lot from them. This is one more study in one species. In response to my comment to add something about what it means to evaluate this hypothesis in a species beyond this one annual (comment 4, I think), the authors have added one sentence in the conclusions that it also might apply to perennials. This does not address my comment. More nuance and an argument would help here.

One more major comment: L115-116- I disagree with this statement. The dispute (and at least one of the cited papers) is about EICA and not about competitive ability. This study stands on its own, and if the idea is to move away from EICA, I would delete this part about disputed. Presumably some of the dispute is not because of a lack of rigor from previous studies, but because other mechanisms lead to success of invasive plants, and we should not expect all species to have increased competitive ability. I appreciate the acknowledgment of all the mechanisms in the next paragraph. This paragraph is could be more concise, and less hand wavy.

Minor comments:

L69 says: “Non-native Erigeron populations have evolved increased competitive ability compared with native populations, but drought stress may alter these differences between ranges.”

What does ‘alter these differences mean?’

L48- 49 says: “Competition is typically more intense under low abiotic stress and weakens or even turns into facilitation under high stress”

Typically yes, but not always. Competition can also increase when resources are limited (e.g. Michalet et al., 2014 https://doi.org/10.1111/jvs.12123; Foxx & Fort, 2019, https://doi.org/10.1371/journal.pone.0220674), and when high densities reduce local resource availability (e.g. Goldberg, 1990). The latter is acknowledged later, but worth being more precise on the former.

L101-103 – To help the reader keep track of all the experiments and what they found, I think it’s worth being clear here that you’re measuring biomass of vegetative plants after 12 weeks or something like that (could frame as proportion of growing season). Also worth being more precise that while survival was slightly lower, it was also very high – very few plants died.

L130-131 – Please be more precise. What comparison is the p-value for? Only non-native, right? Native pops don’t differ significantly between wet & dry according to the letters in Fig 2. I think it’s okay to acknowledge the large amount of variation. I could not quite a handle on how large the effect size is from these plots.

L195-197 – Importantly, its inclusion does not contradict our overall findings but rather makes the overall observed range effect more conservative, as the non-native range includes a lineage with relatively low competitive ability.
It took me a long time to understand this argument. I think it is worth writing something more clearly about how it’s not all pops everywhere that are more competitive in the non-native range?

L205-207 – Shouldn’t it say Cluster 1 in this sentence?

L321 – I encourage the authors to drop the phrase ‘model plant. This paragraph could be much shorter. My original query was to ask for more context as to how what we know about Erigeron might link to other species, but adding all this text, and then L305-307 doesn’t actually address this point.

L340 Here I think it's important to provide the key result in the response letter – that more variation is observed among populations compared to within.

L382 –This study took place in the introduced range? Say so. (And after the double blind is over, say where the experiment took place and the company where the soil came from)

Reviewer #3

(Remarks to the Author)
The authors have addressed all my suggestions and concerns in their response and updated manuscript, and I happy to recommend the manuscript be accepted for publication.

Open Access This Peer Review File is licensed under a Creative Commons Attribution 4.0 International License, which permits use, sharing, adaptation, distribution and reproduction in any medium or format, as long as you give appropriate credit to the original author(s) and the source, provide a link to the Creative Commons license, and indicate if changes were made.
In cases where reviewers are anonymous, credit should be given to 'Anonymous Referee' and the source.
The images or other third party material in this Peer Review File are included in the article’s Creative Commons license, unless indicated otherwise in a credit line to the material. If material is not included in the article’s Creative Commons license and your intended use is not permitted by statutory regulation or exceeds the permitted use, you will need to obtain permission directly from the copyright holder.
To view a copy of this license, visit https://creativecommons.org/licenses/by/4.0/

Reviewer #1 (Remarks to the Author):

Comment: This work was a pleasure to review. It is of exceptional quality in testing the EICA hypothesis. Previous studies often relied on limited sampling sizes (often 10 or fewer populations with limited geographic reach), used a simple measure of biomass as indication of competitive ability. Low sample sizes often resulted in outcomes that were contradictory. The authors here overcome many of these problems by having enormous sample sizes in native and non-native regions, directly test both intra- and interspecific competition under two different scenarios (mesic, dry) using appropriate experimental designs, and add genetic data to their analyses. Their results are convincing and the MS is easy to read and appropriately illustrated. The Supplementary Information is expansive and supports the main conclusions using a serious of supporting experiments

> ***Response:*** *We thank Reviewer #1 for the positive evaluation of our manuscript. Below, we address the comments and suggestions in detail.*

I have few suggestions for the authors, nothing major to critique. I am not familiar with the genetics work, so I am unable to critically address that part of the investigation. Here are a few issues I like the authors to address/correct:

Comment: 1. I like for the authors to sharpen their questions or hypotheses. As written in Q1 and Q3, they are not addressing a single question, but often a complex of interactions. Maybe hypotheses would be more appropriate?

> ***Response:*** *We have now formulated the questions as hypotheses to better reflect their complexity (LINES: 67-73). Accordingly, we have also revised the subheadings in the Statistical analyses section (LINES: 560-561; 568-569; 595-596).*

Comment: 2. I am concerned about collecting seeds from only a single individual in the field. There is no description on how these individuals were selected.

> ***Response:*** *We thank the reviewer for raising this important point. We understand that sampling a single individual per population ignores within-population genetic variation. We provide three reasons why we believe this sampling strategy was appropriate for our system.*
>
> *First, our main goal was to test our hypothesis at a large-scale population level (see LINES: 59-62 and 489-493). Given our treatment design, each seed family needed to be represented by six individuals (3 competition x 2 drought treatments). Our design prioritised broad population sampling across biogeographical gradients rather than greater replication within populations, allowing stronger inference about population-level patterns across the species' native and non-native ranges. With 284 populations, one seed family per population already resulted in 1704 pots.*
>
> *Second, using more than one seed family per population may lead to pseudoreplication with respect to the range comparison. This is especially relevant in the context of our pedigree mixed-effects models (see Rosche et al.[1]).*

Third, previous work on Erigeron *has shown generally low genetic variation within populations but substantial differentiation among populations*[1,2]*. Many populations even consist of only one genotype*[1,2]*, indicating that sampling many populations is a more effective use of research effort than including more individuals per population. Furthermore, methodological reviews suggest that covering environmental heterogeneity across the species' range can be more informative for detecting adaptive patterns than increasing the number of individuals per population*[3,4]*.*

As for the selection of individuals in the field, the individual was selected to represent established plants within each population while avoiding very small or damaged individuals. We have now clarified this approach in the Methods section of the revised manuscript the following way (LINES: 340-344):

Comment: 3. While extensive, the experiments were done in very small pots, considering that up to 5 individuals of different species were grown for several months. The pictures in the Supplemental show seedlings. Would plants become pot-bound, or resource limited? I don't think there are pictures of the full sized or terminal pots that the authors make available.

Response: *We agree that the pot size may restrict root growth in a way that limits plant growth compared with natural field conditions.*

To minimise the effects of resource limitation, the pots were filled with a nutrient-rich soil mixture consisting of compost, topsoil and sand (1:1:1 mixture). Nutrient availability may have decreased during the experiment, but many individuals reached typical field sizes, typically between 70 and 100 cm in height (the tallest individual was around 150 cm). During our field sampling, the average plant height at seed ripening was 103.5 cm. We included this information into the caption of Supplementary Fig. 1.

Competition for space was intentional because we wanted to create a competitive environment, i.e., competition for light and resources. This size limitation likely promoted early-stage competitive interactions during rapid growth. Importantly, any potential size limitation would have affected all populations equally and is therefore unlikely to bias the biogeographical comparisons central to our study.

In natural environments, Erigeron *often grows in highly space-restricted substrates such as asphalt cracks in urban habitats, where rooting space might be even more limited than in experimental pots.* Erigeron *can also be often found on roofs with virtually no substrate. The other four species are also ruderal species that can thrive in such habitats with very little substrate. Therefore, although pot size restricts absolute growth, the experimental conditions still appear to allow realistic competitive interactions among individuals, as shown in our results.*

To address potential issues related to the nutrient availability, we improved the Methods section (LINES: 384-390).

Thank you for the recommendation to better illustrate plant growth under adult growth conditions! We have added a photograph showing the plants at the final stage of the experiment immediately before harvest. This image has been included as Supplementary Figure 1c in the revised Supplementary Information (attached here too).

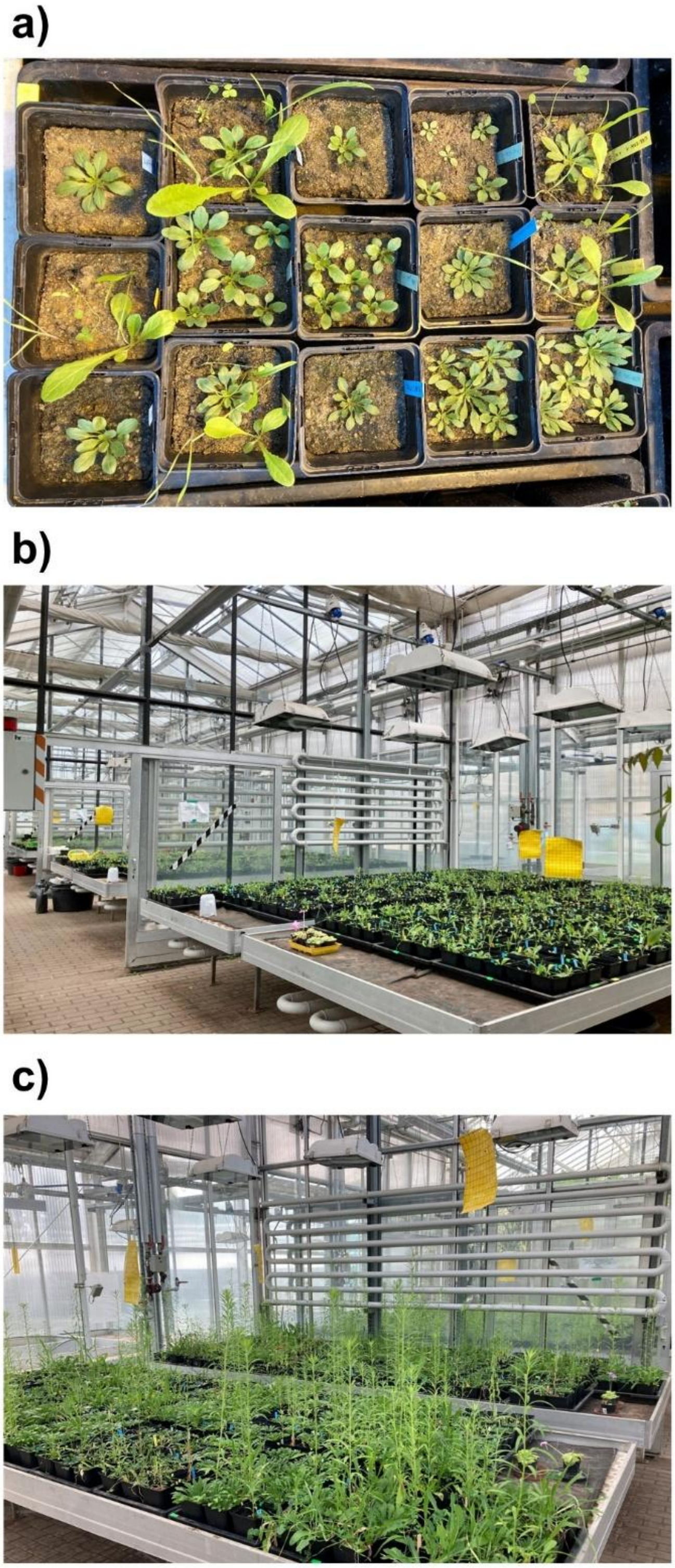

Supplementary Fig. 1: *Experimental setup testing the competitive ability of* Erigeron. *a, Fifteen experimental pots on a tray. These pots differed in the applied competition treatments, having pots without competition (only one Erigeron in the middle), interspecific (one* Erigeron *in the middle and the four competitor species in the corners) and intraspecific (one* Erigeron *in the middle and four other* Erigeron *individuals sourced from the same population in the four corners). The pots within a tray received one type of drought treatment (either mesic or dry). The tray on the photo received the dry treatment. b, Distribution of the* Erigeron *pots in the glasshouse. The position of single pots within*

the trays (every Monday), the distribution of pots across trays (every Wednesday), and the position of the trays within the glasshouse (every Friday) were randomized during the whole experiment. c, Erigeron *pots before the harvest. Plants were harvested 18 weeks after sowing them in the pots. The photo shows that many* Erigeron *individuals reached sizes comparable to those observed in the field, typically between 70 and 100 cm in height (maximum ~150 cm). During field sampling, the average plant height at seed ripening, was 103.5 cm.*

Comment: 4. I like to see a discussion of how choice of the experimental organism as an annual may influence the findings. Is the ability of an annual plant to respond to changes in selection pressure elevated compare dot species that are more long-lived? On the other hand, if Erigeron is mostly self pollinating, as claimed, how can we expect such rapid evolution to occur? These topics appear less well-developed in the discussion.

Response: *We appreciate this important comment that life history and mating system may influence the evolutionary dynamics.* Erigeron canadensis *is a model species for rapid evolution*[1,4]*. For example, it was the first eudicot weed to evolve glyphosate resistance*[5]*, which has occurred multiple times and independently at multiple locations*[6,7]*.*

First, annual species may respond more rapidly to changes in selection pressure because of their short generation times and the large number of generations that can occur over relatively short time periods[8]*. At the same time, although* Erigeron canadensis *is predominantly self-pollinating, studies show that occasional outcrossing and repeated introductions can maintain genetic variation within the invasive range, enabling evolutionary responses to novel environments. Most importantly, we argue that the genomic mechanisms of rapid evolution are not necessarily related to outcrossing. Large-effect genomic variants may instead arise from other mating-independent mechanisms such as transposon activity and structural variation caused by recombination, even in selfing germ lines*[9]*. An additional advantage of selfing is that, if a novel beneficial mutation arises or is introduced, the highly self-fertilising mating system of* Erigeron *may accelerate the fixation of beneficial genotypes within a population because beneficial alleles are less likely to be diluted by outcrossing*[10]*. We have now expanded the Methods with a more detailed description of the species and its suitability as a model system for investigating rapid evolutionary changes (LINES: 321-331).*

5. In Supplementary Fig. 6 the panels are not appropriately labelled. There are 3 (b).

Response: *Thank you for identifying this mistake! We have corrected the figure. Due to other changes in the main text, the figure is now called Supplementary Figure 7.*

6. In Supplementary Fig.9 the graphs use Conyza instead of Erigeron

Response: *Thank you! We have corrected the figure.*

Reviewer #2 (Remarks to the Author):

Comment: Assessing the mechanisms driving success of invasive species in their new ranges has been a goal in ecological research for several decades. One prominent hypothesis (proposed in 1995) is the evolution of increased competitive abilities (EICA) hypothesis (Blossey and Notzold, J of Ecology). In this current manuscript, the authors evaluate one part of this hypothesis (that of increased competitive ability in the introduced range) with a very large experiment in greenhouse mesocosms with ~250 populations of an invasive plant species collected from across both the native and ranges, coupled with genetic analyses to assess population relatedness. They find that individuals from populations in the introduced range perform better than those from the native range when grown in competition in their experimental setup. The scale of the populations sampled to include in the experiment is remarkable, and the main and supporting greenhouse experiments are substantial. While I appreciate the tremendous amount of work that went into the experiments, including all of the ancillary experiments, overall, I disagree that the results support the interpretation as presented. A full assessment of limitations and stronger engagement with the literature would help put the paper more firmly and in context and make the interpretations more believable.

> ***Response:*** *We thank Reviewer #2 for the evaluation of our manuscript and for recognising the experimental effort underlying this study. We appreciate the reviewer's constructive comments and agree that a more explicit discussion of the limitations, together with stronger engagement with the relevant literature, can help to place our results in a clearer context and strengthen the interpretation of our findings. In the revised manuscript, we have addressed these points by expanding the discussion of potential limitations and incorporating additional references where appropriate. Below, we respond to the reviewer's specific comments in detail.*

Here I provide more detail about my concerns along with suggestions for improving the manuscript.

Comment: 1. The first paragraph of the introduction oversimplifies earlier research. In particular, the original EICA hypothesis was proposed as increased competitive ability in the introduced range arising from escape from enemies leading to a reallocation of resources from defense to growth. It is a difficult hypothesis to test given the multiple pathways that it includes (even as many, many common garden experiments have been done to evaluate one or several components of it). Here the authors instead cite the hypothesis as increased competitive ability as arising from selection pressures from new neighbors in the introduced range. This is both an incorrect citation of the original 1995 paper, and I think also gets at one of my core concerns about the manuscript. I agree that the current study definitively shows that plants from invasive populations can grow larger across a range of conditions (drought, wet, different densities and compositions of neighbors), but I am not convinced the design is sufficient to evaluate whether it is selection from neighbors that is the mechanism driving the changes in traits.

> ***Response:*** *We thank the reviewer for pointing this out. We fully agree that our previous wording did not accurately reflect the original formulation of the EICA hypothesis. We also agree with the reviewer that EICA is based on shifts in herbivory, rather than plant competition. Most importantly, we agree that our data cannot provide mechanistic evidence for EICA. However, our intention was not to introduce or test EICA as a central framework in this study. For this reason, EICA was not explicitly stated in the Introduction and was first mentioned in the Discussion of the previous version of our manuscript (Line 230 therein). The original sentence that the reviewer most likely refers to aimed to highlight the*

general observation that invasive populations often exhibit increased competitive ability, rather than to attribute this pattern to a specific mechanism. The original sentence was:

"A central phenomenon of invasions is that novel interactions with resident species can select for larger size and increased competitive ability in non-native ranges[5,6,7,8]".

With the following references:

5. Blossey, B. & Nötzold, R. Evolution of increased competitive ability in invasive nonindigenous plants: a hypothesis. J. Ecol. 83, 887–889 (1995).

6. Catford, J. A., Bode, M. & Tilman, D. Introduced species that overcome life history tradeoffs can cause native extinctions. Nat. Commun. 9, 2131 (2018). 563

7. Montesinos, D., Graebner, R. C. & Callaway, R. M. Evidence for evolution of increased competitive ability for invasive Centaurea solstitialis, but not for naturalized C. calcitrapa. Biol. Invasions 21, 99–110 (2019).

8. Callaway, R. M., Lucero, J. E., Hierro, J. L. & Lortie, C. J. The EICA is dead? Long live the EICA! Ecol. Lett. 25, 2289–2302 (2022).

The combination of references may have unintentionally created the impression that our study focuses on EICA. We thank the reviewer for their comment, as it highlighted that the selection of references may have led to the impression that our manuscript focuses on EICA. We have therefore revised the Introduction to avoid this ambiguity.

Instead, the purpose of the first paragraph is to emphasize that increased competitive ability in non-native ranges has been frequently reported, but that empirical tests have yielded inconsistent results. Our aim is to address this discrepancy by focusing on limitations in previous approaches and by providing a more robust framework for testing differences in competitive ability across ranges. In this sense, our study focuses on improving how this phenomenon is quantified, rather than on identifying the specific evolutionary mechanisms underlying it.

We have made four major changes to address this issue. (1) We have revised the wording in the Abstract (LINES: 6-8). (2) We have revised the wording and citations in the Introduction to avoid the impression that we aimed to test a specific mechanistic framework such as EICA (LINES: 26-34). (3) We now use the last paragraph of the Introduction to clearly state that we did not aim to identify the specific mechanisms of increased competitive ability (LINES: 65-67). (4) We have revised the first section of the Results and Discussion to include the EICA hypothesis along with other potential mechanisms for the observed increased competitive ability (LINES: 117-129).

Comment: 2. While the genetic analyses seem key to ruling out some of the alternative hypotheses, the story seems more complex than described here. The main result that introduced pops have higher competitive ability is only supported by clusters 1 and 2, and cluster 4 (which is not present in the native range), actually has the lowest competitive ability. I would thus like to see the results presented with more cautionary language, and acknowledge the pieces of them that point in a different direction. It also seems important to compare the results (split by genetic clusters) in the drought treatment vs. control in the main text.

Response: *We thank the reviewer for this important comment and agree that the genetic clustering results reveal a more complex pattern than our previous wording suggested. In the revised manuscript, we have adopted more cautious wording. We have clarified that increased competitive ability in the non-native range was supported by two clusters (Clusters 1 and 2), whereas other clusters showed different patterns. In particular, we now explicitly acknowledge that Cluster 4, which occurs only in the non-native range, exhibited comparatively low competitive ability. At the same time, we note that Cluster 4 was not present in the native range and therefore cannot be used to directly test evolutionary differences between ranges. Its inclusion does not contradict our main conclusion but rather reflects the fact that our sampling captures additional genetic diversity present in the non-native range. Importantly, because Cluster 4 shows relatively low competitive ability, its inclusion makes our estimate of the overall non-native competitive advantage more conservative. We have clarified this point in the revised manuscript to avoid potential misinterpretation (LINES: 179–197).*

In addition, we have added to the main text a comparison of the competitive ability of the different genetic clusters in the mesic and drought treatments. This analysis shows that only Cluster 1 exhibited a significant difference between treatments, whereas the other clusters showed similar but non-significant trends (LINES: 202-207).

Comment: 3. How were these 4 competitor species chosen? Where did the seeds for them come from? What does it mean that they are species that Erigeron did not evolve with? The justification in the text is not very clear, but I think their identities are critical. It doesn't seem like the neighbor species provide a particularly competitive environment. Two of them are perennials, and we would expect them to grow more slowly than the focal annual species, and the focal species can grow to 2 m, much larger than any of the others. In Suppl Fig 1, it is already quite apparent that the focal species is much larger than any of the others, which don't seem that likely to be providing much competition, even if they're all rather weedy species. I would like more context provided, and a consideration of how greenhouse results might or might translate to the field.

Response: *We thank the reviewer for raising this important point regarding the need to more precisely describe the selection criteria of the four species. However, we argue that the selected species did provide effective competition, as competition reduced Erigeron biomass by 63.4%, i.e. to less than half of its value when grown alone (Extended Data Fig. 2) across all populations (LINES: 101-103). To estimate the competitive ability of the competitor species, we conducted Ancillary Experiment 6, which showed that their competitive ability was comparable to that of* Erigeron *(Extended Data Fig. 6).*

The four species were selected based on three main criteria: (1) frequent co-occurrence with Erigeron *in vegetation surveys across both the native and non-native ranges, (2) representation of different functional groups to avoid phylogeny-specific competition effects, and (3) contrasting biogeographic histories relative to* Erigeron *to ensure that the species do not share a long co-evolutionary history.*

(1) To identify suitable competitors, we first asked field collaborators sampling Erigeron *populations across its ranges to report the most commonly co-occurring species. From these field surveys, we identified twenty candidate species.*

(2) Given the list of species, we assigned them to four functional groups (Asteraceae, non-related herb, N-fixing legume, and grass).

(3) We then selected species that do not share a native range with Erigeron*, leaving us with eleven candidate species.*

To ensure that the chosen species were capable of competing with Erigeron *during early growth stages, we conducted a preliminary growth experiment in which seedlings of the candidate species were grown individually under the same greenhouse conditions. During the first four weeks, the candidate competitors generally produced substantially greater biomass than* Erigeron*, indicating that they were capable of providing meaningful competitive pressure during early development (see newly added Supplementary Figure 10).*

From these candidates we selected four species based on their germination success and their representation of different functional groups: Lactuca serriola, Plantago lanceolata, Trifolium repens*, and* Bromus tectorum*.*

We have revised the respective part in the Methods to increase clarity (LINES: 366-379). Below, please find the newly added Supplementary Fig. 10, along with its caption:

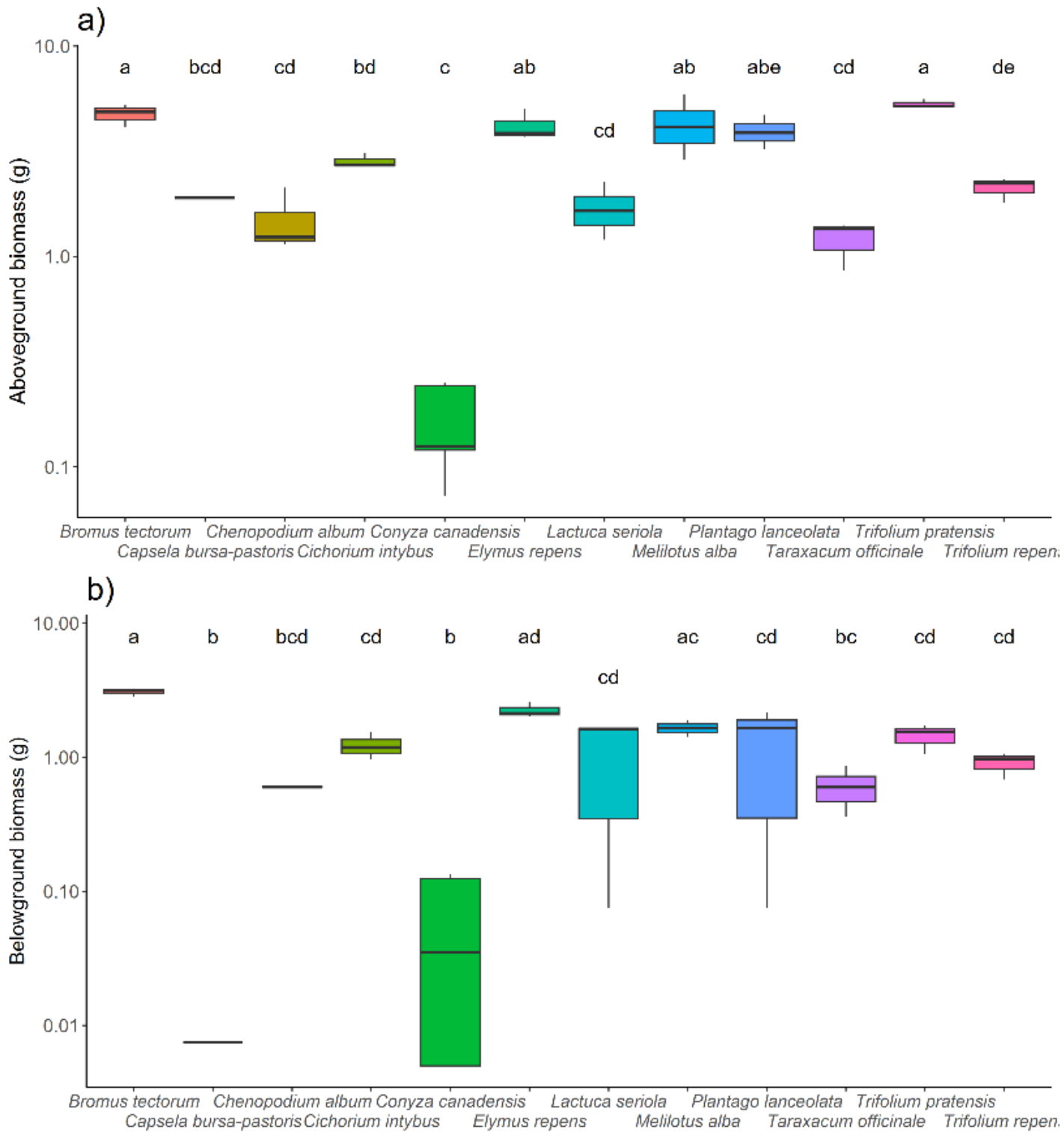

Supplementary Fig. 10: Preliminary screening of candidate competitor species. *Eleven candidate competitor species were grown individually under greenhouse conditions for four weeks to assess their early growth performance. After harvest, aboveground and belowground biomasses were measured following drying. Across species, candidate competitors produced substantially greater biomass than* Erigeron*, indicating their ability to exert competitive pressure during early developmental stages. Lowercase letters indicate groupings based on Tukey's post hoc tests.*

Regarding the apparent size differences between Erigeron *and the competitor species in Supplementary Fig. 1, we agree that the provided photographs may give the impression*

that the focal species dominates its neighbours. However, plant size in these images reflects only a subset of genotypes and does not represent the variation across the whole experiment. We thank the reviewer for carefully inspecting the Supplementary Figures because this showed us that the choice of the photo in Figure S1 was not representative.

To better illustrate the variability, we have added additional photographs from the experiment to this response letter. These images include examples from Ancillary Experiment 6 (a,b), where competitor species were grown alone and in competition, as well as images from the main experiment (c). These photographs demonstrate that competitor species, particularly Lactuca serriola*, can achieve comparable or even greater biomass than* Erigeron*, and that competitive outcomes vary depending on the identity and competitive ability of the focal genotype.*

Importantly, plant size differences were not consistent across the experiment but depended strongly on genotype and competitive context. While highly competitive Erigeron *individuals may overtop their neighbours, less competitive genotypes frequently did not, resulting in substantial overlap in plant size among species. This variation is also reflected in the quantitative results presented in the manuscript.*

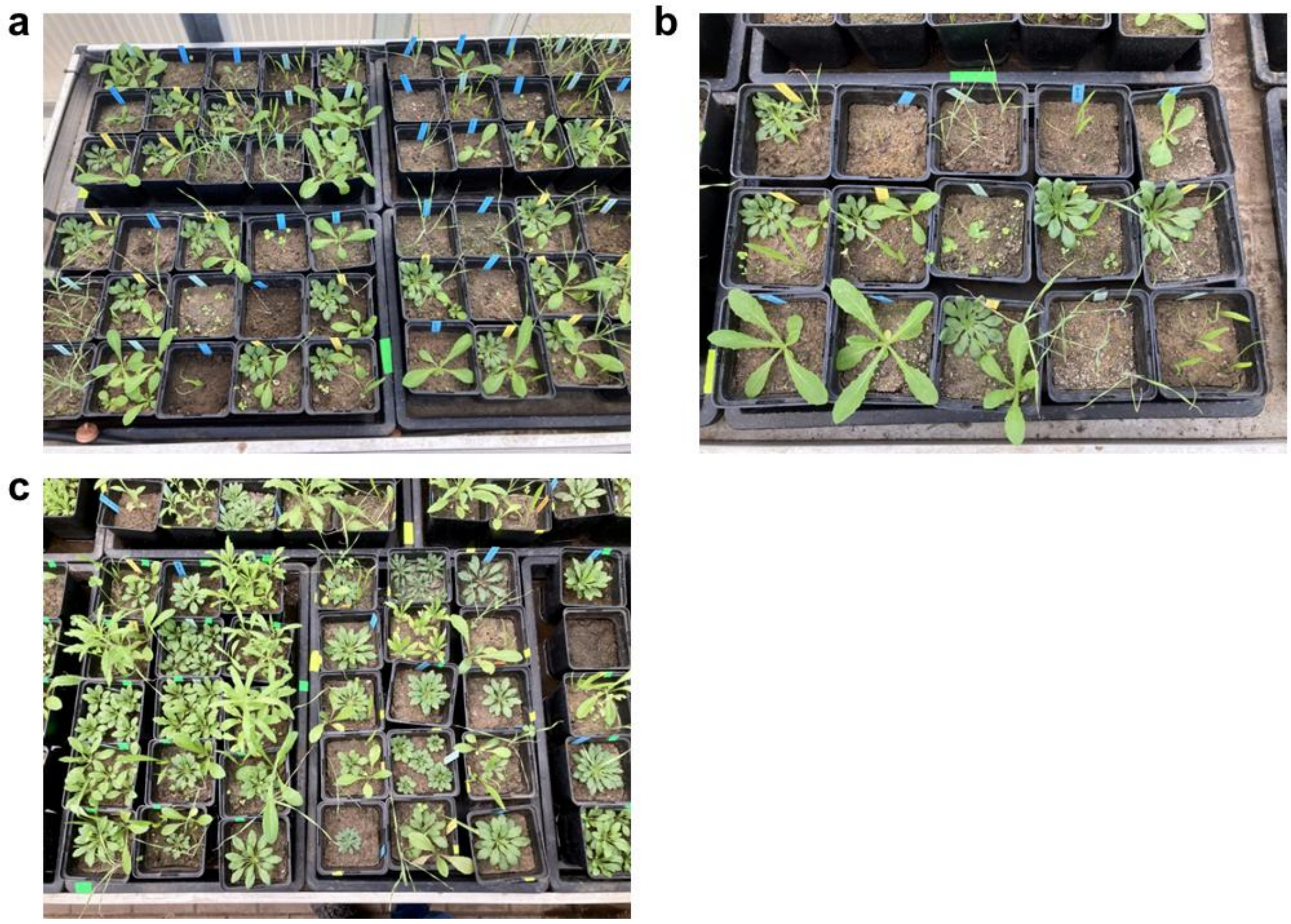

Variation in plant size and competitive interactions among* Erigeron *and competitor species. *(a-b) Images from Ancillary Experiment 6 showing growth of competitor species under different conditions 6 weeks after germination.* ***c****, Image from the main experiment 10 weeks after germination. At this stage, substantial variation in plant size among competitor species is evident, depending on the identity and competitive ability of the focal* Erigeron *genotype.*

In addition, we agree that the provided information on the origin of the seed was not clear. We have added the missing information in the Competitor selection section (LINES: 379-380).

We also agree that including both annual and perennial competitors may influence competitive dynamics because annual invaders can indeed suppress perennial seedlings during early growth stages, whereas perennials might become stronger competitors at later stages[11]*. In this context, we additionally acknowledge the species-specific nature of our approach and its generalisability to field conditions. We have clarified these points and added a discussion of potential limitations of greenhouse competition experiments and their translation to field conditions within the Results and Discussion (LINES: 251-253), and Conclusions (LINES: 304-306) section.*

Comment: 4. The conditions in which the plants were grown matter. We know this from common garden studies in general, and more specifically from studies that have conducted multiple common gardens of the same invasive species (e.g. Coulatti et al 2009, Evol. Applications & 2016 book chapter in Invasion Genetics; Williams et al. 2008, Oecologia). Tell us what soil was used, what day length, temperature, humidity and supplemental lighting in the greenhouse were used – and explain how the choices were made. In particular – despite the drought treatment, are these plants growing in a nutrient rich environment? Presumably with no soil biota?

Response: *We thank the reviewer for highlighting the importance of clearly describing greenhouse growing conditions. First, we now acknowledge that greenhouse conditions simplify natural environments*[12]*, and that future studies should test whether these patterns persist in the field (see above, LINES: 253-255). We have also incorporated the fact that outcomes of plant–plant interactions depend on soil biota into the Discussion, where we acknowledge that interactions with soil biota (e.g. mycorrhizal fungi) may have contributed to variation in competitive ability among populations (LINES: 125-129). In addition, we have now expanded the Methods section to provide additional details on temperature, light regime, humidity conditions, and soil composition, including an explanation for these choices where appropriate (LINES: 384-390 and LINES: 407-414). In this context, we also now explicitly state that the soil used in the experiment was not sterilised to avoid alterations of soil structure and to allow plants to interact with soil biota, thereby maintaining more realistic belowground conditions:*

Comment: 5. The literature on competition and coexistence is entirely overlooked beyond the citation of the Hart et al paper, and I think provides important context, and needs to be engaged with. For example, we know that competitive ability depends on the environment (e.g. Van Dyke et al 2022, Nature; Wainwright et al 2019, J Ecol) and more recent work has considered evolution in this context (e.g. Germain et al. 2023 J Ecol). Engagement with this literature particularly in the intro (L44-68) and then in the interpretation of the results would help with the context.

Response: *We agree that we have overused the reference to Hart et al.*[13]*. We have now revised this throughout the text and incorporated additional references.*

In addition, we have revised the relevant paragraph, by including the references of Van Dyke et al[14]*; Wainwright et al.*[15]*. In response to this comment, we have also improved our acknowledgement of the conditionality of competitive outcomes in relation to the abiotic and biotic environment. We changed the paragraph in the Introduction. (LINES: 44-51).*

Comment: 6. I found the conclusion on L268 that no trade-off between drought and competitive ability means the invaders are more likely to win as a large stretch. (I encourage the authors to double check the references throughout this paragraph– to me they are not the most appropriate refs for the conclusions. e.g. why ref 58 to support sentence 1 – a meta-analysis or EICA that isn't acknowledge earlier). Also, even though Suppl Fig 3 shows a significant positive relationship in competitive ability measured in mesic and dry conditions, it is not a strong relationship: the slope of the line does not appear to be that different from zero (would help if the figure were square, and the R2 and slope reported), and there is a ton of variability. I would tread carefully.

Response: *Again, we thank the reviewer for their careful assessment and helpful suggestions.*

First, regarding the Reference 58 (Felker-Quinn et al.[16]), we fully agree that this paragraph is not the appropriate place to cite it. Instead, we have added the citation where it provides important conceptual context, that is, where we speculate about the role of increased competitive ability of non-native populations (Results & Discussion; LINE: 114-116).

Second, following the reviewer's recommendation, we revised the figure to use equal axis scaling, which makes the relationship between competitive ability under mesic and dry conditions easier to interpret. While our analysis shows a positive relationship between competitive ability across watering treatments (slope = 0.26; $P < 0.001$), we agree that there is substantial variability among populations and now explicitly acknowledge this when reporting the result (LINES: 135-137).

Third, we agree that the implications regarding the absence of a trade-off between drought and competitive ability were overinterpreted. We have therefore reconsidered the conclusions drawn in this paragraph and revised the text to present a more cautious interpretation of the results (LINES: 298-302).

Comment: 7. Ancillary experiment 5 is critical to interpreting these results, but more care needs to be taken in interpreting the results. It is difficult to examine the strength of the correlation, because the axes are on different scales (and the figs are not square). Does the experiment with one density over or underestimating competitive ability? Shouldn't these results lead to more caution in the absolute values reported in the main text?

Response: *We thank the reviewer for these helpful suggestions. First, we agree that non-squared figures may affect the perception of relationships. We have adjusted all figures presenting linear regression models to use equal axis scaling throughout the main text.*

Second, we also agree that Ancillary Experiment 5 is critical for our results, and according to the recommendation, we have changed the scales of Extended Data Fig. 5 which we included in our response, making the strength of correlation more visible. Please note that the differing scales between the density-dependent and simplified approaches are expected and arise from fundamental differences in how competitive ability is estimated. In the density-dependent approach, competitive ability is derived from regression-based estimates of per capita interaction coefficients (α). In contrast, the simplified with-versus-without competitor approach captures the cumulative effect of competition on performance, inherently leading to the lower α values which translate into slightly higher absolute values of competitive ability (approx. 1.5 times higher). In other words, the difference in scale reflects differences in measurement frameworks rather than inconsistencies between the

approaches. This suggests that the simplified approach does not systematically over- or underestimate competitive ability but instead provides a comparable ranking of populations while differing in absolute magnitude. The latter is particularly important for our analyses. However, to acknowledge the potential density dependency of competitive interactions we improved our Results and Discussion according to the recommendation (LINES: 255-258).

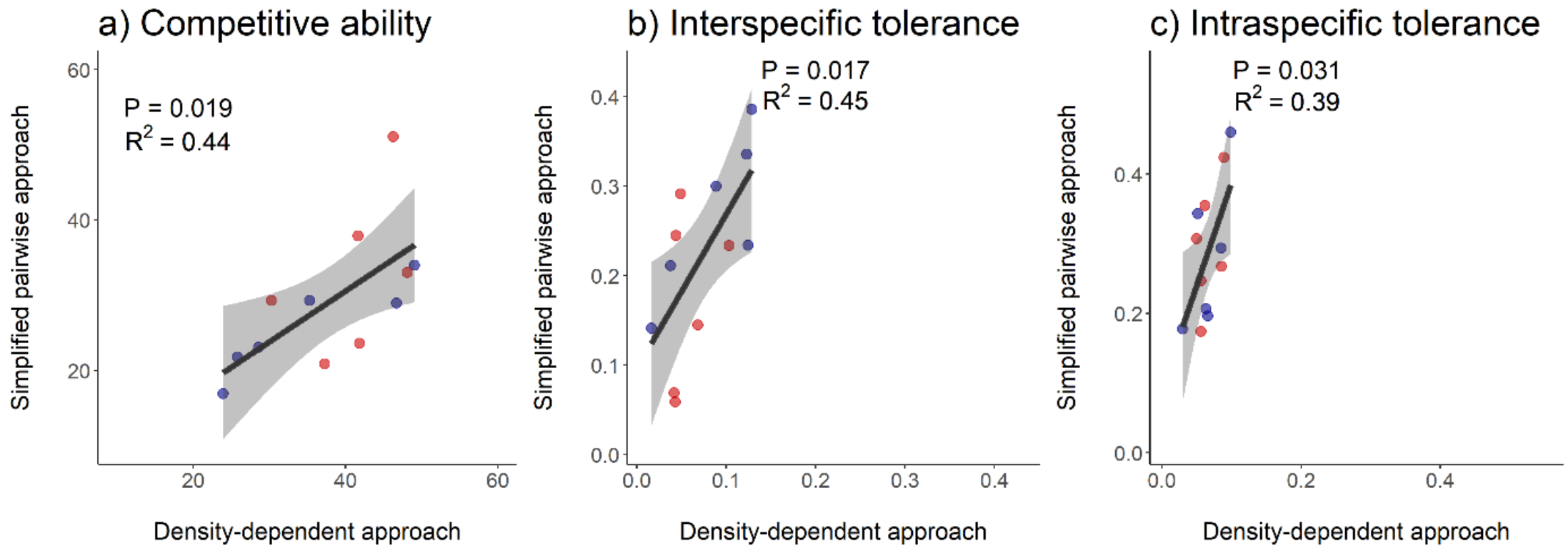

Extended Data Fig. 5: Relationship between the measures of competitive interactions in our main experiment (simplified pairwise comparison: with vs. without competitors) and those estimated using a density-dependent approach (i.e., along a competitor density gradient)*. The panels show the relationships for a) competitive ability, b) interspecific competitive tolerance, and c) intraspecific competitive tolerance. The estimates were compared for six populations (three native (blue) and three non-native (red)* Erigeron *populations; Supplementary Table 1) across both drought treatments. Specifically, we compared values from the main experiment with those from a density experiment (ancillary experiment with 540 pots; Ancillary Experiment 5 in Supplementary Note 1). Most importantly, both estimates capture consistent relative differences among populations. This suggests that the simplified approach does not systematically over- or underestimate competitive ability but instead provides a comparable ranking of populations while differing in absolute magnitude. The x- and y-axes show competition estimates from the density-dependent and simple pairwise approach, respectively. The P- and* R^2*- values represent the results of linear regression models for the comparison between the estimates from the two different experimental sources. Shaded bands represent the 95% confidence intervals of the linear regressions.*

A few more minor comments:

Comment: 8. The study system needs more details. What is the invasion history of Erigeron? When was it introduced to Eurasia? Do we know anything about the invasion pathway? What kind of habitat does it occur in? (My impression is that it's a species that's adapted to disturbance, which provides a different lens to consider competition in). Does the community biomass shown in Fig 1 include Erigeron?

> ***Response:*** *We agree that requested further information on the study species facilitate interpretation of our findings. Based on the suggestions, we have now added more detailed information on its habitat preference, introduction and dispersal history in the Methods section (LINES: 312-320).*
>
> *In addition, we clarified that the community biomass collected include* Erigeron *biomass (LINES: 345-346).*

Comment: 9. L438 – competitive ability of which species?

> ***Response:*** *We meant the competitive ability among the four competitor species and* Erigeron*. We have corrected the sentence (LINES: 515-518).*

Comment: 10. L 465 – I must have missed the reporting of these results, but this is repeated measurements and running a series of t-tests is not appropriate

> ***Response:*** *The results of these analyses are presented in Extended Data Fig. 1. We redid the analyses by using repeated measures analyses and corrected the related description of the statistical methods (LINES: 551-554).*

Comment: 11. The physiology measurements are not well incorporated in the manuscript – could be dropped.

> ***Response:*** *We thank the reviewer for this suggestion. We agree that the physiological measurements are not central to the main focus of our study. Because they provide important validation that our drought treatment induced a physiologically meaningful stress response in the plants, which may be relevant for some readers, we prefer not to remove them altogether. However, we removed any details from the main manuscript and left only a small comment in caption of Figure 1 to avoid disturbing the reading flow with these side results (LINES: 91-92).*

Comment: 12. I think Extended data fig 2 is important – it is critical to see the raw data not just the estimates of competition coefficients, and I think worth reporting at least something about the magnitude of seed production reduction in the main text (not just comparing competitive abilities). That said, I was surprised how small these plants are, and even more surprised that a plant with 1 g of dry weight could have 300 capitula.

Response: *We thank the reviewer for this helpful comment and agree that Extended Data Fig. 2 provides important context by showing the raw biomass data underlying our estimates of competitive ability. We now added information about the magnitude (LINES: 108-111). We also appreciate the suggestion to report reductions in seed production. However, in our main experiment, plants were harvested during the vegetative phase (mid-July) and therefore did not reach full reproduction. In* Erigeron*, capitula production typically begins after ~120–150 days and can continue for several weeks, with full reproductive output often only reached after ~200 days under greenhouse conditions. As a result, it was not feasible to quantify seed production within the timeframe of the competition experiment.*

To address this limitation, we conducted the Ancillary Experiment 3, in which a subset of plants was grown until full reproductive maturity. In this experiment, we found a strong positive relationship between aboveground biomass at harvest and final capitula production (Extended Data Fig. 3). This provides evidence that competition induced biomass reduction potentially correlates with capitula reduction. We now improved the figure caption to clarify this in a clearer way (LINES: 883-885).

Reviewer #3 (Remarks to the Author):

Comment: In this manuscript, the authors describe a glasshouse experiment of impressive scale utilizing the invasive weed *Erigeron canadensis*. Using hundreds of populations spanning native and invaded ranges and exposed to mesic and dry conditions, they investigate competitive ability while accounting for population ancestry — an important but still relatively rare consideration in experiments of this scale.

The authors report increased competitive ability in invaded populations relative to native populations, regardless of drought stress. At the level of genetic clusters (based on ADMIXTURE analysis of ddRADseq genotypes), two clusters showed increased competitive ability in the invaded range, a third did not show a significant difference, and a fourth contained only representatives of the invaded range. Using separate competition treatments, they determine that the increased competitive ability arises from interspecific rather than intraspecific interactions.

I found the manuscript to be well written and carefully edited, with clear figures and, for the most part, results that support the authors' conclusions. The study provides strong empirical evidence for a long-hypothesized feature of invasive species. Given the scale of the data strength of the results, I believe it would make a solid contribution to the Nature Communications. However, I have several comments that I believe would strengthen the manuscript:

> ***Response:*** *We thank Reviewer #3 for the positive and thoughtful evaluation of our manuscript and for recognising the scale and strength of our experimental design. We are grateful for the constructive comments and suggestions, which we believe have helped to further improve the clarity of the manuscript. We address the specific points raised below.*

Comment: 1. It is somewhat unexpected that genetic cluster 4 is not represented in the native range, given the authors' assertion that the native range was comprehensively sampled. Could cluster 4 represent admixture or post-introduction divergence within the invaded range? Additional visualization of genetic structure (e.g., a PCA showing cluster assignment and native vs. invaded) would help clarify the relationships among clusters and support the interpretation of evolutionary independence.

> ***Response:*** *We thank the reviewer for this helpful suggestion and agree that the absence of Cluster 4 in the native range requires clarification. The intriguing pattern may reflect a combination of multiple introductions, admixture, and post-introduction divergence in the non-native range*[17]*. These possibilities may have been facilitated by its annual life cycle and the relatively long invasion history*[1]*, as* Erigeron *has been established in Europe since the 17th century*[18].
>
> *Notably, the cluster is not completely absent in the native range but occurs in some individuals in the Northwest USA with a probability of > 20%. This pattern is only visible when considering the probabilities of individual assignment to each cluster (see Supplementary Fig. 5 and Supplementary Table 1). In the main manuscript, we only show the most likely cluster, so we effectively reduce a continuous variable to a single categorical assignment (Fig. 1). We now clarify more explicitly in the text that there is also continuous variation within clusters.*
>
> *Nevertheless, Cluster 4 remains strongly overrepresented in the non-native range. The few occurrences of Cluster 4 in the native range could reflect source genotypes that further evolved and spread in the non-native range but could also reflect reintroduction from non-*

native regions. Our data do not allow us to distinguish between these possibilities. Based on a neighbour-joining tree, Cluster 4 most likely evolved from Cluster 3, either in the native or non-native range. We have therefore aimed to keep the wording as neutral as possible, adding explanation of the overrepresentation in the main text without becoming too speculative (LINES: 197-202).

To support this interpretation, we followed the reviewer's suggestion to add additional figures. We have added a PCA, as desired by the reviewer, and, in addition, a neighbour-joining tree visualising the genetic structure (Supplementary Fig. 5b,c). Both figures include information on cluster assignment on a continuous scale and native vs. non-native populations, which helps clarify the relationships among clusters and their distribution across ranges

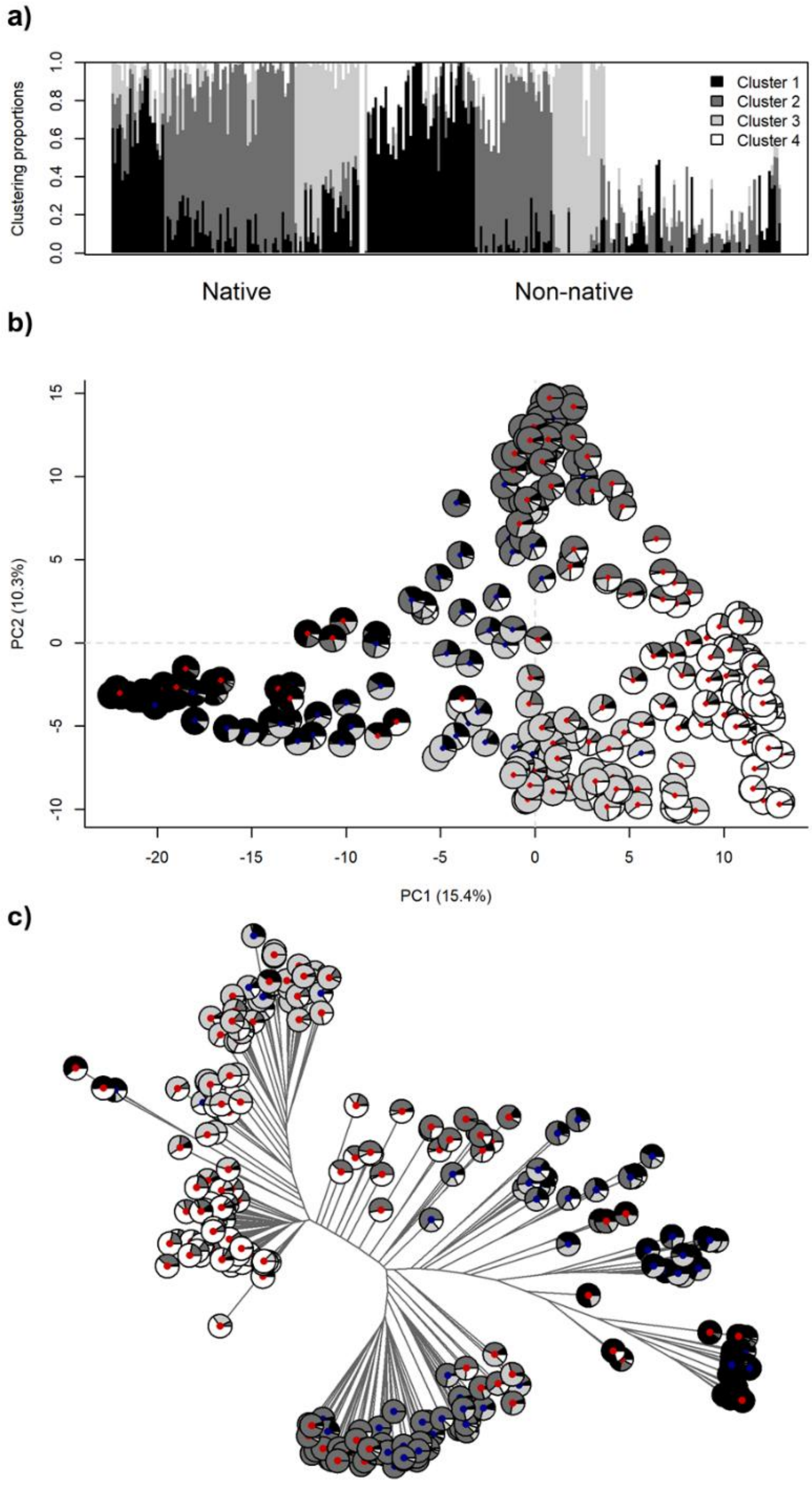

Supplementary Fig. 5: Genetic structure of *Erigeron* populations. a, Bar plot of individuals from 265 populations showing their posterior assignment probabilities to four genetic clusters (K = 4), as inferred by Bayesian clustering analysis. Each individual is represented by a vertical bar partitioned into four segments corresponding to cluster membership proportions. **b,** Principal component analysis (PCA) of population genomic data (ddRADseq) showing the distribution of populations along the first two principal components (percentage of explained variance indicated on axes). Each pie chart represents a population. **c,** Neighbour-joining tree based on Nei's genetic distances among populations. Edge lengths reflect genetic distances. Each node represents a population, displayed as a pie chart indicating cluster membership proportions. Colours correspond to clusters: Cluster 1 (black), Cluster 2 (dark grey), Cluster 3 (light grey), and Cluster 4 (white). Populations are grouped by geographic range (native (blue) vs. non-native (red)). The number of clusters (K = 4) was supported by an elbow plot (Supplementary Fig. 4).

Comment: 2. The authors interpret increased competitive ability in two introduced clusters as evidence for multiple independent evolutionary origins of this trait. However, it would be helpful to quantify the extent of gene flow among clusters within the invaded range. If substantial gene flow has occurred, elevated competitive ability in multiple clusters could reflect introgression from a single origin rather than independent evolution. Providing estimates of inter-cluster gene flow would strengthen the inference of repeated evolution.

> ***Response:*** *We thank the reviewer for this insightful suggestion. We agree that gene flow among clusters is an important consideration when interpreting patterns of repeated evolution. However, several lines of evidence suggest that extensive introgression among clusters is unlikely to explain our results.*
>
> *First,* Erigeron canadensis *is a highly selfing species (selfing rate ≈ 0.96*[1]*), which strongly limits effective gene flow and introgression among genetic lineages. While occasional admixture may occur, widespread genetic exchange sufficient to homogenise adaptive traits across clusters is unlikely*[19]*.*
>
> *Second, the geographic distribution of clusters indicates limited opportunities for gene flow between the clusters showing increased competitive ability (Fig. 1). Cluster 1 is primarily distributed in southwestern Europe and North Africa, whereas Cluster 2 predominates in East Asia, with only limited overlap (e.g. southwestern Europe). In large parts of the invaded range, particularly in Asia, Cluster 2 occurs largely in the absence of Cluster 1, reducing the likelihood of substantial gene exchange between them.*
>
> *Third, the genetic structure itself supports their evolutionary independence. The recently added PCA and neighbour-joining analyses (Supplementary Fig. 5b,c) show clear differentiation among clusters, with Cluster 1 being the most genetically distinct and Cluster 2 diverging separately from Clusters 3 and 4. If increased competitive ability were driven by introgression from a single origin, we would expect a more uniform distribution of this trait across clusters.*
>
> *Taken together, these patterns suggest that increased competitive ability most likely evolved independently in multiple genetic backgrounds. We have clarified this reasoning in the revised manuscript (LINES: 179-202) and added additional visualisation of genetic structure (Supplementary Fig. 5) to support our interpretation.*

Comment: 3. The authors account for population co-ancestry using pedigree mixed-effects models in the primary analyses. However, when examining how competition varied among genetic clusters, the authors revert to standard mixed models including only admixture cluster as a fixed explanatory variable. It is unclear why this was done but it would be helpful to clarify whether the results are robust to modelling continuous relatedness rather than discrete cluster identity alone.

> ***Response:*** *We thank the reviewer for this important point. In the primary analyses, we used pedigree mixed-effects models (PMMs) to account for continuous genetic relatedness among populations via the co-ancestry matrix. However, when analysing variation among genetic clusters, our aim was to explicitly test the effect of discrete genetic structure (i.e. cluster identity) on competitive ability. Because admixture clusters are derived directly from the same genetic data as the co-ancestry matrix, including both cluster identity and the co-ancestry structure in the same model would introduce substantial collinearity. Moreover, when comparing the relative importance of fixed and random effects between PMMs and MMs (see Fig. 3a), such an approach may result in circular reasoning. For this reason, we used standard mixed-effects models for the cluster-based analyses to isolate the*

effect of genetic clustering. We have clarified this rationale in the Methods section (LINES: 591-594).

Comment: 4. It is not clear whether maternal environmental effects were controlled (e.g., via a common-garden generation). A brief clarification or discussion of the potential influence of maternal effects would further strengthen the evolutionary interpretation.

Response: *We thank the reviewer for this important point. The environment experienced by maternal plants can influence offspring performance (i.e. maternal effects) and could, in principle, contribute to our results. We considered the use of a common-garden (F1) generation. However, in our greenhouse experience, additional generations often produce smaller and less viable seeds, likely due to artificial conditions, which may introduce bias rather than reduce it. Moreover, greenhouse cultivation may weaken field-derived competitive signals, potentially diluting adaptive responses mediated by epigenetic effects. Given these constraints, and the large number of populations included, we prioritised using the sampled seeds over additional generations. To reduce this potentially confounding effect, we standardised early ontogeny by selecting seedlings of similar size across populations. We have now clarified this point in the manuscript and acknowledge this limitation explicitly (LINES: 391-395).*

Comment: 5. In Fig. 4 I suggest re-labelling the y-axes "Competitive…of Erigeron" or just drop "of Conyza" altogether.

Response: *Thank you so much for finding this mistake. We have now corrected the y-axes to "Competitive ... of* Erigeron*".*

REFERENCE LIST

1. Rosche, C. *et al.* Climate outweighs native vs. nonnative range-effects for genetics and common garden performance of a cosmopolitan weed. *Ecol. Monogr.* **89**, e01386 (2019).
2. Bhattacharya, S. *et al.* Genetic diversity and population structure of invasive and native populations of *Erigeron canadensis* L. *J. Plant Ecol.* **15**, 864–876 (2022).
3. Santos, A. S. & Gaiotto, F. A. Knowledge status and sampling strategies to maximize cost-benefit ratio of studies in landscape genomics of wild plants. *Sci. Rep.* **10**, 3706 (2020).
4. Lucas, M. S. *et al.* Re-focusing sampling, design and experimental methods to assess rapid evolution by non-native plant species. *Biol. Invasions* **26**, 1327–1343 (2024).
5. VanGessel, M. J. Glyphosate-resistant horseweed from Delaware. *Weed Sci.* **49**, 703–705 (2001).
6. Okada, M. *et al.* Evolution and spread of glyphosate resistance in *Conyza canadensis* in California. *Evol. Appl.* **6**, 761–777 (2013).
7. Yuan, J. S. *et al.* Functional genomics analysis of horseweed (*Conyza canadensis*) with special reference to the evolution of non–target-site glyphosate resistance. *Weed Sci.* **58**, 109–117 (2010).
8. Hamann, E., Weis, A. E. & Franks, S. J. Two decades of evolutionary changes in *Brassica rapa* in response to fluctuations in precipitation and severe drought. *Evolution* **72**, 2682–2696 (2018).
9. Munasinghe, M. *et al.* Combined analysis of transposable elements and structural variation in maize genomes reveals genome contraction outpaces expansion. *PLOS Genet.* **19**, e1011086 (2023).
10. Peischl, S., Kirkpatrick, M. & Excoffier, L. Expansion load and the evolutionary dynamics of a species range. *Am. Nat.* **185**, E81–E93 (2015).
11. Seabloom, E. W., Harpole, W. S., Reichman, O. J. & Tilman, D. Invasion, competitive dominance, and resource use by exotic and native California grassland species. *Proc. Natl. Acad. Sci.* **100**, 13384–13389 (2003).
12. Batstone, R. T., Peters, M. A. E., Simonsen, A. K., Stinchcombe, J. R. & Frederickson, M. E. Environmental variation impacts trait expression and selection in the legume–rhizobium symbiosis. *Am. J. Bot.* **107**, 195–208 (2020).

13. Hart, S. P., Freckleton, R. P. & Levine, J. M. How to quantify competitive ability. *J. Ecol.* **106**, 1902–1909 (2018).

14. Van Dyke, M. N., Levine, J. M. & Kraft, N. J. B. Small rainfall changes drive substantial changes in plant coexistence. *Nature* **611**, 507–511 (2022).

15. Wainwright, C. E., HilleRisLambers, J., Lai, H. R., Loy, X. & Mayfield, M. M. Distinct responses of niche and fitness differences to water availability underlie variable coexistence outcomes in semi-arid annual plant communities. *J. Ecol.* **107**, 293–306 (2019).

16. Felker-Quinn, E., Schweitzer, J. A. & Bailey, J. K. Meta-analysis reveals evolution in invasive plant species but little support for Evolution of Increased Competitive Ability (EICA). *Ecol. Evol.* **3**, 739–751 (2013).

17. De Kort, H. *et al.* Life history, climate and biogeography interactively affect worldwide genetic diversity of plant and animal populations. *Nat. Commun.* **12**, 516 (2021).

18. Kowarik, I. Historical evidence for context-dependent assessment of *Erigeron canadensis* invasions in an 18th-century European landscape. *NeoBiota* **89**, 1–15 (2023).

19. Pickup, M. *et al.* Mating system variation in hybrid zones: facilitation, barriers and asymmetries to gene flow. *New Phytol.* **224**, 1035–1047 (2019).

Reviewer #1 (Remarks to the Author):

Comment: I have reviewed the changes the authors have made and these have improved the manuscript. I appreciate the level of detail the authors have gone to in explaining their reasoning and the changes they made.

Response: *We thank Reviewer #1 for the positive evaluation of our manuscript.*

Reviewer #2 (Remarks to the Author):

Comment: In their revision, the authors have now more carefully framed their manuscript, and take somewhat more care in distinguishing their hypotheses and questions from the EICA hypothesis. They have addressed most of my concerns, along with those of the other reviewers. Below I bring back a few concerns I think still need addressing and one major point on the framing.

> ***Response:*** *We thank Reviewer #2 for their evaluation of our manuscript and for recognising the experimental efforts underlying this study. We appreciate the reviewer's constructive comments and agree that a more explicit discussion of the limitations, together with stronger engagement with the relevant literature, can help to place our results in a clearer context and strengthen the interpretation of our findings. In the revised manuscript, we have addressed these points by expanding the discussion of potential limitations and incorporating additional references where appropriate. Below, we respond to the reviewer's specific comments in detail.*

Major point on framing:

Throughout the introduction and discussion, the authors emphasize that their study is more rigorous and complete than any that have come before. While I appreciate the tremendous effort that went into the experiments and the genomics, in general, just doing a bigger experiment does not necessarily make a study a better contribution. I would be more compelled if this language were used more sparingly, particularly in the discussion. Dozens if not hundreds of studies have examined the possibility that invasive species are more competitive where they are introduced, and we have learned quite a lot from them. This is one more study in one species. In response to my comment to add something about what it means to evaluate this hypothesis in a species beyond this one annual (comment 4, I think), the authors have added one sentence in the conclusions that it also might apply to perennials. This does not address my comment. More nuance and an argument would help here.

> ***Response:*** *Response: We thank the reviewer for this thoughtful comment. We agree that the previous version placed too much emphasis on the relative scope and rigour of our study compared with earlier work. Throughout the Introduction, Discussion, figure legends, and Abstract, we have therefore revised the wording to avoid unnecessary comparisons with previous studies, removed claims of the quality of the work, and adopted a more balanced interpretation of our findings (for examples, please see: LINES: 180-184; 192-193; 207-212; 221; 370). In addition, we removed several self-promoting sentences throughout the text.*
>
> *We also appreciate the suggestion to better discuss the implications of using an annual species. We have expanded the section "Ecological and evolutionary implications" to clarify why rapid evolutionary responses may be more readily detectable in annual species with short generation times, while also acknowledging that similar evolutionary processes may occur in perennial or clonally reproducing invaders (LINES: 386-390). We hope this revised discussion more clearly addresses the broader applicability and limitations of our findings beyond* Erigeron canadensis.

One more major comment: L115-116- I disagree with this statement. The dispute (and at least one of the cited papers) is about EICA and not about competitive ability. This study stands on its own, and if the idea is to move away from EICA, I would delete this part about disputed. Presumably some of the dispute is not because of a lack of rigor from previous studies, but because other mechanisms lead to success of invasive plants, and we should not expect all species to have increased competitive ability. I appreciate the acknowledgment of all the mechanisms in the next paragraph. This paragraph is could be more concise, and less hand wavy.

> ***Response:*** *We thank the reviewer for this helpful suggestion and agree with the comment. Our intention was not to reintroduce the debate surrounding the EICA hypothesis or to imply that increased competitive ability should be expected in all invasive species. Rather, our study focuses on testing whether increased competitive ability occurs in* Erigeron canadensis*. To avoid overinterpreting our findings and to better align the text with this aim, we have removed the final sentence of the paragraph.*

Minor comments:

L69 says: "Non-native Erigeron populations have evolved increased competitive ability compared with native populations, but drought stress may alter these differences between ranges."

What does 'alter these differences mean?'

> ***Response:*** *We thank the reviewer for pointing out that this wording was unclear. By "alter these differences", we meant that drought stress could modify the magnitude or even the direction of the differences in competitive ability between native and non-native populations. Our hypothesis was that drought stress might reduce or eliminate the difference in competitive ability between ranges, as environmental stress often decreases the intensity of competition[3,4]. However, following the editorial request, we have removed the hypotheses from the manuscript, and this statement is therefore no longer included.*

L48- 49 says: "Competition is typically more intense under low abiotic stress and weakens or even turns into facilitation under high stress"

Typically yes, but not always. Competition can also increase when resources are limited (e.g. Michalet et al., 2014 https://doi.org/10.1111/jvs.12123; Foxx & Fort, 2019, https://doi.org/10.1371/journal.pone.0220674), and when high densities reduce local resource availability (e.g. Goldberg, 1990). The latter is acknowledged later, but worth being more precise on the former.

> ***Response:*** *We thank the reviewer for this important clarification and agree that our original wording was too general. We have now revised the Introduction to acknowledge that, although competition often weakens with increasing abiotic stress, the outcome is context dependent. In particular, the effects of resource limitation depend on the type of stress, the resources limiting growth, and the balance between above- and belowground competition. We therefore modified the text (LINES: 207-212).*

L101-103 – To help the reader keep track of all the experiments and what they found, I think it's worth being clear here that you're measuring biomass of vegetative plants after 12 weeks or something like

that (could frame as proportion of growing season). Also worth being more precise that while survival was slightly lower, it was also very high – very few plants died.

> ***Response:*** *We thank the reviewer for this helpful suggestion. We have now clarified that aboveground biomass was measured after 12 weeks of growth, corresponding to the main vegetative growth phase of* Erigeron *before peak reproduction (LINES: 234-235). We also now state that overall survival remained very high throughout the experiment (96.8%), although drought caused a small but statistically significant reduction (3.2%) (LINES: 237-239). We agree that these additions make the biological interpretation of the results clearer.*

L130-131 – Please be more precise. What comparison is the p-value for? Only non-native, right? Native pops don't differ significantly between wet & dry according to the letters in Fig 2. I think it's okay to acknowledge the large amount of variation. I could not quite a handle on how large the effect size is from these plots.

> ***Response:*** *We thank the reviewer for pointing this out. The reported 12.9% reduction and the corresponding P-value refer to the overall main effect of drought across both native and non-native populations, rather than to comparisons within each range separately. We have now clarified this in the text to avoid confusion (LINES: 265-266). We agree that the pairwise comparisons shown in Fig. 2 indicate a significant difference only within the non-native range, whereas the native populations show the same trend but do not differ significantly. Nevertheless, the mixed-effects model detected a significant overall effect of drought across all populations, but not in interaction with range. We also agree that there is considerable variation among populations. As our populations originate from a wide range of climatic conditions, spanning local aridity gradients in both the native and non-native ranges, such variation is expected. To account for this, we included climatic water deficit as a covariate in our statistical models. We therefore believe that the overall estimate appropriately reflects the effect of drought while accounting for environmental differences among populations.*

L195-197 – Importantly, its inclusion does not contradict our overall findings but rather makes the overall observed range effect more conservative, as the non-native range includes a lineage with relatively low competitive ability.

It took me a long time to understand this argument. I think it is worth writing something more clearly about how it's not all pops everywhere that are more competitive in the non-native range?

> ***Response:*** *We thank the reviewer for pointing this out. We agree that our previous wording was not sufficiently clear. Our intention was to explain that Cluster 4, despite exhibiting comparatively low competitive ability, occurs predominantly in the non-native range and is therefore included in the overall comparison between native and non-native populations. As such, the estimated range effect is conservative because the non-native range includes a cluster with relatively low competitive ability. Despite this, non-native populations still exhibited significantly greater competitive ability overall. We have now rewritten this section to explain this reasoning more explicitly and to clarify that increased competitive ability was not observed uniformly across all non-native populations or genetic clusters (LINES: 318-321).*

L205-207 – Shouldn't it say Cluster 1 in this sentence?

Response: *We thank the reviewer for spotting this error. The reviewer is correct—it should refer to Cluster 1. We have now corrected this in the revised manuscript (LINE: 329).*

L321 – I encourage the authors to drop the phrase 'model plant. This paragraph could be much shorter. My original query was to ask for more context as to how what we know about Erigeron might link to other species, but adding all this text, and then L305-307 doesn't actually address this point.

Response: *We thank the reviewer for this helpful comment. We have removed the term "model species" and refined this sentence to better explain why* Erigeron *is well suited for studying rapid evolution. We decided to keep the paragraph on the study species because reviewers in the first round requested additional background information on its biology and evolutionary characteristics. We now better understand the reviewer's concern and have also revised the corresponding sentence in the former Conclusions (now called "Ecological and evolutionary implications") section to more clearly acknowledge that our findings are based on an annual species while avoiding overgeneralisation. We hope these revisions address the reviewer's concerns (LINES:411-412).*

L340 Here I think it's important to provide the key result in the response letter – that more variation is observed among populations compared to within.

Response: *We thank the reviewer for this helpful suggestion. We agree that the rationale for our sampling strategy can be explained more clearly. Our objective was to maximise the number of sampled populations rather than the number of individuals within populations, because previous studies have shown that* Erigeron canadensis *exhibits relatively low genetic variation within populations owing to its predominantly selfing mating system (Rosche et al. 2019; Sheng et al. 2022). By sampling a single representative individual from a much larger number of populations, we substantially increased the geographic coverage of the study and, consequently, our ability to detect large-scale evolutionary differentiation across the species' range. We have now clarified this rationale in the revised manuscript (LINES: 432-436).*

L382 –This study took place in the introduced range? Say so. (And after the double blind is over, say where the experiment took place and the company where the soil came from)

Response: *The experiment was conducted in the native range of* Erigeron canadensis*, in Butte, Montana, USA. As the double-blind review process has now ended, we have included the location of the glasshouse experiment in the Methods. We have also specified that the soil mixture was purchased from Wagner Nursery (Butte, USA) (LINES: 475-481).*

Reviewer #3 (Remarks to the Author):

Comment: The authors have addressed all my suggestions and concerns in their response and updated manuscript, and I happy to recommend the manuscript be accepted for publication.

Response: *We thank the reviewer for the positive evaluation.*

References

Rosche, C. *et al.* Climate outweighs native vs. nonnative range-effects for genetics and common garden performance of a cosmopolitan weed. *Ecol. Monogr.* **89**, e01386 (2019).

Sheng, M. *et al.* Acquisition and evolution of enhanced mutualism—an underappreciated mechanism for invasive success? *ISME J.* **16**, 2467–2478 (2022).
